# Synthesis of Bis(1,2,3-Triazole) Functionalized Quinoline-2,4-Diones †

**DOI:** 10.3390/molecules23092310

**Published:** 2018-09-10

**Authors:** David Milićević, Roman Kimmel, Martin Gazvoda, Damijana Urankar, Stanislav Kafka, Janez Košmrlj

**Affiliations:** 1 Department of Chemistry, Faculty of Technology, Tomas Bata University in Zlin, 760 01 Zlin, Czech Republic; david.milicevic@gmail.com (D.M.); r.kimmel@centrum.cz (R.K.); 2 Faculty of Chemistry and Chemical Technology, University of Ljubljana, SI-1000 Ljubljana, Slovenia; martin.gazvoda@fkkt.uni-lj.si (M.G.); damijana.urankar@fkkt.uni-lj.si (D.U.)

**Keywords:** click chemistry, azido group, quinoline-2,4(1*H*,3*H*)-diones, propargyl group, bis(1,2,3‑triazole)

## Abstract

Derivatives of 3-(1*H*-1,2,3-triazol-1-yl)quinoline-2,4(1*H*,3*H*)-dione unsubstituted on quinolone nitrogen atom, which are available by the previously described four step synthesis starting from aniline, were exploited as intermediates in obtaining the title compounds. The procedure involves the introduction of propargyl group onto the quinolone nitrogen atom of mentioned intermediates by the reaction of them with propargyl bromide in *N*,*N*-dimethylformamide (DMF) in presence of a potassium carbonate and the subsequent formation of a second triazole ring by copper catalyzed cyclisation reaction with azido compounds. The products were characterized by ^1^H, ^13^C and ^15^N NMR spectroscopy. The corresponding resonances were assigned on the basis of the standard 1D and gradient selected 2D NMR experiments (^1^H–^1^H gs-COSY, ^1^H–^13^C gs-HSQC, ^1^H–^13^C gs-HMBC) with ^1^H–^15^N gs-HMBC as a practical tool to determine ^15^N NMR chemical shifts at the natural abundance level of ^15^N isotope.

## 1. Introduction

**Figure 1:**
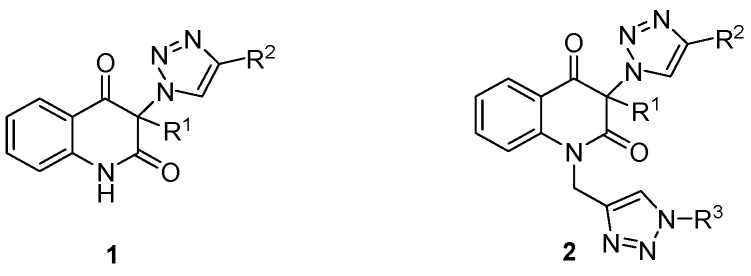
A general structure of 1,2,3-triazole quinoline-2,4-diones **1** (**left**) and the bis(1,2,3-triazole) counterparts **2** (**right**).

The 1,4-disubstituted-1,2,3-triazole heterocyclic motif has become an exceedingly popular structure finding applications in a broad range of areas including materials, biomaterials, metallopharmaceuticals, supramolecular chemistry, chemical sensing and catalysis, to name just a few [1]. In coordination and organometallic chemistry, for example, it became an important ligand scaffold, not only because of simplicity and reliability in its preparation, but also due to a variety of coordination modes offering [2,3,4,5,6]. Owing to the discovery of copper(I)-catalyzed 1,3-cycloaddition of terminal alkynes with organic azides, the CuAAC click reaction, the preparation of 1,4-disubstituted-1,2,3-triazole is facilitated in mild and modular fashion [7,8]. Although this “click triazole” has become a part of a broad range of molecules, its association with quinoline-2,4-diones remains largely underdeveloped. Apart from our recent publication on 3-(1*H*-1,2,3-triazol-1-yl)quinoline-2,4(1*H*,3*H*)-dione derivatives (**1**, Figure 1) [9], to the best of our knowledge, other 1,2,3-triazole functionalized quinoline-2,4-diones are unprecedented.

As part of our endeavor in quinoline-2,4-dione chemistry [10] as well as functional click triazoles [11,12] and their applications [13], we became interested in the synthesis of bis(1,2,3-triazole) functionalized quinoline-2,4-diones **2** that may potentially serve as functional scaffolds in coordination chemistry, molecular sensing and biochemistry. It is noteworthy that many compounds with the quinoline-2,4-dione structure were isolated from fungi, bacteria and plants, possessing broad range of interesting biological activities in vitro and in vivo [10]. Herein we report an approach to quinoline-2,4-diones unsymmetrically substituted with two click triazoles, an extensive ^1^H, ^13^C, and ^15^N NMR spectral analyses, and a preliminary investigation of their chelating properties towards arene-ruthenium.

## 2. Results and Discussion

**Scheme 1:**
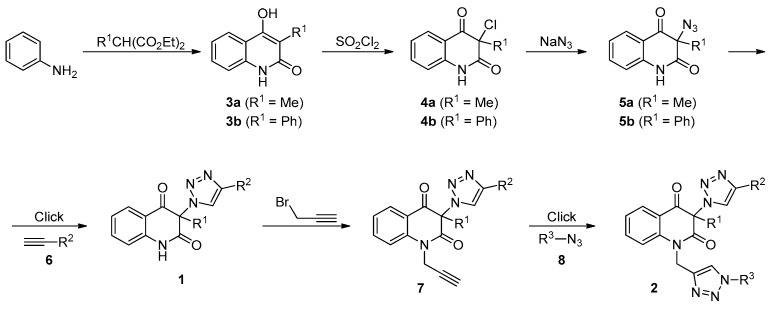
Preparation of bis(1,2,3-triazole) functionalized quinoline-2,4-diones **2**.

We reasoned that the desired bis(1,2,3-triazole) functionalized quinoline-2,4-diones **2** could be obtained via previously described 3-(1*H*-1,2,3-triazol-1-yl)quinoline-2,4(1*H*,3*H*)-dione derivatives **1** as synthetic intermediates (Scheme 1). The latter were prepared in a four-step reaction sequence starting from aniline, which upon treatment with diethyl 2-methylmalonate and diethyl 2-phenylmalonate initially afforded the corresponding 4-hydroxyquinolin-2(1*H*)-ones **3a** and **3b** [14]. Chlorination with sulfuryl chloride into 3-methyl- and 3-phenyl-3-chloroquinolin-2,4(1*H*,3*H*)-diones **4a** [15] and **4b** [16], followed by the nucleophilic displacement of the chlorine atoms with sodium azide, gave 3-methyl- and 3-phenyl- substituted 3-azidoquinoline-2,4(1*H*,3*H*)-diones **5a** and **5b** [16]. Then we began with copper-catalyzed azide-alkyne cycloaddition reaction (CuAAC).

Although a large variety of reaction conditions have been developed for the CuAAC reaction [17,18], our previous work in this field has shown that for 3-azidoquinoline-2,4(1*H*,3*H*)-diones a combination of copper(II) sulfate pentahydrate and elemental copper (CuSO_4_/Cu^0^) in dimethyl sulfoxide (DMSO) provided results that were superior to other combinations. Adopting those previous results in this work some additional optimizations of the reaction conditions were carried out with **5a** and phenylacetylene (**6a**) as the model substrates. Screening through the reaction solvents indicated that *N*,*N*-dimethylformamide (DMF) is even more efficient than DMSO, providing the desired target compound **1a** in shorter reaction times. The influence of the amount of granular copper to the course of the reaction between **5a** and equimolar amount of **6a** in DMF was also briefly investigated. While keeping the loading of CuSO_4_·5H_2_O constant at 10 mol % relative to **5a**, the amount of the elemental copper was varied from 380 mol % to 100 mol %. The results are summarized in Table 1.

Based on the above, in a general procedure, a mixture of 3-azidoquinoline-2,4(1*H*,3*H*)-dione (**5**, 1.0 mmol), a slight excess of terminal alkyne **6** (1.05 mmol), CuSO_4_⋅5H_2_O (0.12 mmol), and granular copper (2.0 mmol) in DMF (2.3 mL) was stirred at room temperature, in the presence of air. In addition to phenylacetylene (**6a**), propargyl alcohol (**6b**) was selected as the acetylene partner. The reactions were completed within 30 min. As indicated in Table 2, the products **1** were obtained in excellent yields. By using a more standard CuSO_4_∙5H_2_O/l-ascorbic acid catalyst in CH_2_Cl_2_/water biphasic system, the cycloaddition between **5a** and **6a** required substantially longer reaction time (48 h) to achieve a similar yield of the product **1a** as compared to the above CuSO_4_/Cu^0^/DMF conditions (Entries 1 and 2).

**Scheme 2:**
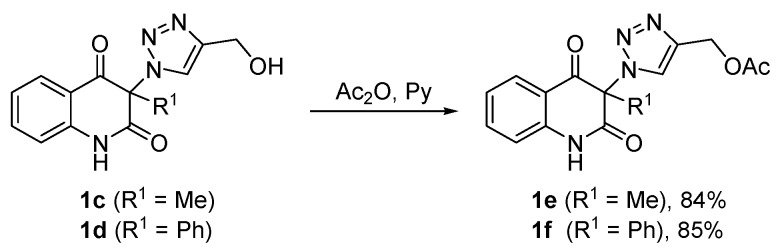
Preparation of compounds **1e** and **1f**.

Prior to the introduction of propargyl group at the N1 nitrogen atom of the quinoline-2,4(1*H*,3*H*)-dione ring in **1**, the primary hydroxyl groups at **1c** and **1d** were protected by acetylation by using acetic anhydride in pyridine as shown in Scheme 2. The corresponding acetates **1e** and **1f** were obtained in 84–85% yields.

Alkylation of compounds **1a**,**b**,**e**,**f** with propargyl group was carried out by using 1.5 equivalent of propargyl bromide (**6c**) and 3 equivalents of potassium carbonate in DMF. These reactions proceeded smoothly within 45 min at room temperature. The yields are given in Table 3.

Although *N*-alkylation of the lactam group usually takes place preferentially in quinoline-2,4(1*H*,3*H*)-diones [10], the competitive *O*-alkylation has been documented in similar systems [19]. The N1 position of thus introduced propargyl group in **7** was confirmed by 2D NMR spectroscopy in particular by the presence of the long-range correlations between the propargyl methylene protons and carbon atoms C-8a and C-2 in the ^1^H-^13^C HMBC spectra (in **7a**,**c**,**d**) as well as N1 nitrogen atom in the ^1^H–^15^N *gs*-HMBC spectrum (in **7a**).

As the last step of the reaction sequence shown in Scheme 1, monotriazoles **7** were submitted to a second cycloaddition with selected azides **8** to give the expected bis-triazoles **2**. Benzyl azide (**8a**), azidobenzene (**8b**) and tetrazolo[1,5-*a*]pyridine (**8c**) were selected as the reaction partners. Whereas benzyl azide (**8a**) and azidobenzene (**8b**) readily reacted into the desired products **2a**,**b**,**d**,**e**,**g**,**h**,**j**,**k** at room temperature, tetrazolo[1,5-*a*]pyridine (**8c**), a synthetic equivalent for 2-azidopyridine (**8c’**), required harsher reaction conditions (Table 4). This can be explained by the tetrazolyl form in which compound **8c** exists predominantly at room temperature [11]. As the proportion of the azido isomer increases at elevated temperature, the reactions with **8c** were conducted at 100 °C, to afford compounds **2c**,**f**,**i**,**l** in good yields.

In this case too, some standard click catalyst/solvent combinations were briefly evaluated. The cycloaddition between acetylene **7c** and benzyl azide (**8a**) with CuSO_4_∙5H_2_O/Na-ascorbate (or l-ascorbic acid) pair in CH_2_Cl_2_/water and *t*-BuOH/water solvent systems required prolonged reaction times, providing lower yields of the product **2d** as compared to the CuSO_4_/Cu^0^/DMF conditions (compare Entries 4–7). In the case of *t*-BuOH/water the presence of water in the reaction mixture turned the reactants and products into a gummy material that stuck to the reaction vessel and the magnetic stirring bar, impeding the reaction from going to completion, as already noticed for click reactions with highly hydrophobic reagents [20].

**Scheme 3:**
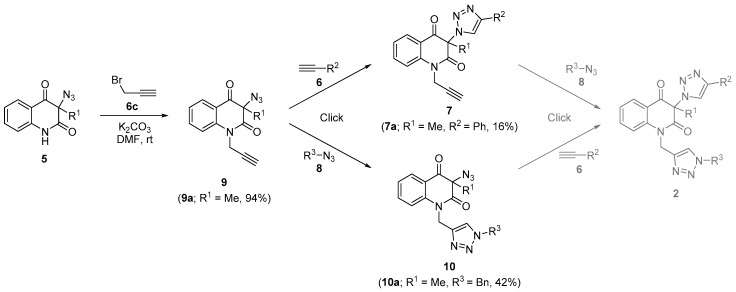
An alternative approach to bis(1,2,3-triazole) functionalized quinoline-2,4-diones **2** through a “propargylation-click-click” reaction sequence.

In principle, the “click-propargylation-click” reaction sequence at 3-azidoquinoline-2,4-diones **5** could be altered, providing the target bis-(1,2,3-triazole) functionalized products **2** via bifunctional azidoethynyl quinoline-2,4-dione intermediate **9** as shown in Scheme 3. This would allow orthogonal sequential synthetic strategies for accessing bis(1,2,3-triazole) functionalized materials [21]. We briefly explored this possibility by treating 3-azido-1-propargylquinoline-2,4-dione derivative **9a** with phenylacetylene (**6a**) or benzyl azide (**8a**) under the above mentioned CuSO_4_/Cu^0^/DMF conditions. The corresponding monotriazoles **7a** (16%) and **10a** (42%), respectively, were obtained in moderate yields.

**Figure 2:**
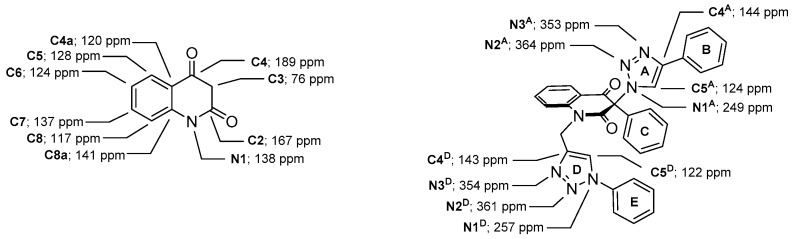
Selected ring and atom numbering along with the chemical shift data (mean values rounded up to whole numbers are provided).

The compounds **2a**–**l** were characterized by ^1^H, ^13^C and, with the exception of **2a**,**g**,**h**, also by ^15^N NMR spectroscopy. The corresponding resonances were assigned on the basis of gradient-selected 2D NMR experiments including ^1^H–^1^H *gs*-COSY, ^1^H–^13^C *gs*-HSQC, ^1^H–^13^C *gs*-HMBC and ^1^H–^15^N *gs*-HMBC. For the atom numbering scheme, see Figure 2. Some characteristic spectral features are discussed below.

The ^13^C and ^15^N chemical shifts for triazole rings **A** and **D** (Table 5 and Table 6) are in a good agreement with those reported previously [11].

**Scheme 4:**
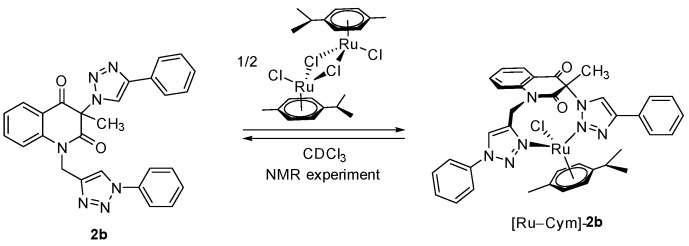
Reaction of **2b** with [RuCl(μ-Cl)(η^6^-*p*-cymene)]_2_ with tentatively proposed structure of the [Ru–Cym]-**2b** complex.

**Figure 3:**
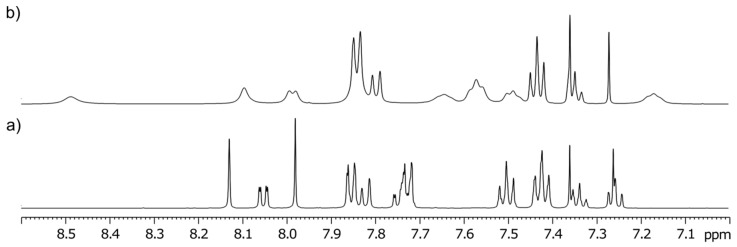
Aromatic region of ^1^H NMR spectra of: (**a**) **2b** in CDCl_3_, and (**b**) a mixture of **2b** (42 mM) and [RuCl(μ-Cl)(η^6^-*p*-cymene)]_2_ (21 mM) in CDCl_3_ immediately after dissolution.

**Figure 4:**
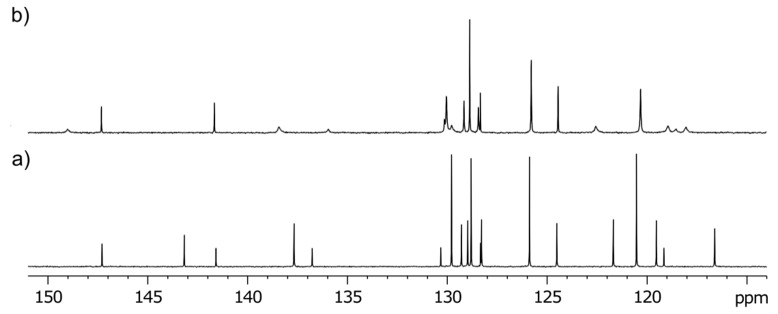
Aromatic region of ^13^C NMR spectra of: (**a**) **2b** in CDCl_3_, and (**b**) a mixture of **2b** (42 mM) and [RuCl(μ-Cl)(η^6^-*p*-cymene)]_2_ (21 mM) in CDCl_3_.

To preliminarily assess the applicability of bis-triazole compounds **2** as ligands, we decided to examine their coordination abilities to arene-ruthenium. NMR experiment was designed in which compound **2b** and equimolar amount of ruthenium (0.5 equiv of [RuCl(μ-Cl)(η^6^-*p*-cymene)]_2_) were mixed in CDCl_3_ in NMR tube at room temperature. CDCl_3_ was selected as the reaction solvent in place of the coordinative DMSO-*d*_6_ to avoid possible interference with the metal center (Scheme 4). The reaction mixture was monitored by time dependent ^1^H NMR spectroscopy indicating an instant change in the resonances for **2b** and *p*-cymene ligands upon mixing to form a new set of resonances that remained unchanged over several days. As shown in Figure 3 and Figure 4, both proton and carbon NMR resonances were severely broadened suggesting the presence of a dynamic process in the solution, presumably an equilibrium with the starting ligand, which can result from a relatively weak ligand-to-metal interaction. Unfortunately, broad NMR resonances prevented an unambiguous structure determination of the product [Ru–Cym]-**2b** through the 2D NMR techniques due to overlap as well as lack of several indicative crosspeaks in the spectra, especially in ^1^H–^15^N *gs*-HMBC. Nevertheless, the analysis of the available NMR data tentatively suggested the coordination of both 1,2,3-triazole rings to the Ru–Cym unit as indicated in Scheme 4. Although the coordination properties of the 1,2,3-triazole nitrogen atom N2 are weak, some of us have previously shown that such chelates can be greatly stabilized through an assistance of auxiliary ligand [22].

Attempts to unambiguously determine the structure of [Ru–Cym]-**2b** by variable temperature NMR techniques, as well as to grow crystals suitable for X-ray, failed. All of the above also applies to compounds **2g** and **2h** that were also preliminarily tested in [Ru–Cym] coordination.

## 3. Materials and Methods

### 3.1. General Experimental Methods

The reagents and solvents were used as obtained from the commercial sources. Compounds **3a** [14], **3b** [14], and **5b** [15], as well as benzyl azide (**8a**) [23], azidobenzene (**8b**) [11], and tetrazolo[1,5-*a*]pyridine (**8c**) [24] were prepared as described in the literature. Column chromatography was carried out on Fluka Silica gel 60 (particle size 0.063–0.2 mm, activity acc. Brockmann and Schodder 2–3). Melting points were determined on the microscope hot stage, Kofler, PolyTherm, manufacturer Helmut Hund GmbH, Wetzlar and are uncorrected. TLC was carried out on pre-coated TLC sheets ALUGRAM^®^ SIL G/UV_254_ for TLC, MACHEREY-NAGEL. NMR spectra were recorded with a Bruker Avance III 500 MHz NMR instrument operating at 500 MHz (^1^H), 126 MHz (^13^C) and 51 MHz (^15^N) at 300 K. Proton spectra were referenced to TMS as internal standard, in some cases to the residual signal of DMSO-*d*_5_ (at *δ* 2.50 ppm) or CHCl_3_ (at *δ* 7.26 ppm). Carbon chemical shifts were determined relative to the ^13^C signal of DMSO-*d*_6_ (39.52 ppm) or CDCl_3_ (77.16 ppm). ^15^N chemical shifts were extracted from ^1^H–^15^N *gs*-HMBC spectra (with 20 Hz digital resolution in the indirect dimension and the parameters adjusted for a long-range ^1^H–^15^N coupling constant of 5 Hz) determined with respect to external nitromethane and are corrected to external ammonia by addition of 380.5 ppm. Nitrogen chemical shifts are reported to one decimal place as measured of the spectrum, however, the data should not be considered to be more accurate than ±0.5 ppm because of the digital resolution limits of the experiment. Chemical shifts are given on the *δ* scale (ppm). Coupling constants (*J*) are given in Hz. Multiplicities are indicated as follows: s (singlet), d (doublet), t (triplet), q (quartet), m (multiplet) or br (broadened). Infrared spectra were recorded on FT-IR spectrometer Alpha (Bruker Optik GmbH Ettlingen, Ettlingen, Germany) using samples in potassium bromide disks and only the strongest/structurally most important peaks are listed. Electron impact mass spectra (EI) were recorded on a Shimadzu QP–2010 instrument at 70 eV. HRMS spectra were recorded with Agilent 6224 Accurate Mass TOF LC/MS system with electrospray ionization (ESI). Elemental analyses (C, H, N) were performed with FlashEA1112 Automatic Elemental Analyser (Thermo Fisher Scientific Inc., Waltham, MA, USA).

### 3.2. General Procedure for the Synthesis of 3-Chloroquinoline-2,4(1H,3H)-Diones 4 (Scheme 1)

The 3-Chloroquinoline-2,4(1*H*,3*H*)-diones **4a** [15] and **4b** [16], were prepared from 4‑hydroxyquinolin-2(1*H*)-ones **3a** [14] and **3b** [14], respectively, according to the procedures described in the literature.

**3-Chloro-3-methylquinoline-2,4(1*H*,3*H*)-dione (4a).** Compound **4a** (19.71 g, 94.0 mmol, 94%) was prepared from **3a** (17.52 g, 100 mmol). Yellow crystals, m.p. 178–181 °C (benzene), m.p. [15] 172 °C (acetic acid—water); *R*_f_ = 0.52 (30% ethyl acetate in chloroform); ^1^H NMR (500 MHz, CDCl_3_) *δ* 1.99 (s, 3H), 7.06 (d, 1H, *J* = 8.0 Hz), 7.22 (dd, 1H, *J* = 7.6, 7.6 Hz), 7.59–7.66 (m, 1H), 8.02 (d, 1H, *J* = 7.7 Hz), 9.41 (s, 1H); ^13^C NMR (126 MHz, CDCl_3_) *δ* 21.2, 62.8, 116.7, 118.1, 124.5, 129.1, 136.8, 139.6, 169.2, 188.4; IR (cm^−1^): *ν* 3203, 3072, 3004, 2940, 1709, 1674, 1614, 1600, 1486, 1439, 1379, 1239, 770, 440; MS (EI) *m*/*z* (%): 212 (4, [M + 3]^+^), 211 (33, [M (^37^Cl)]^+^), 210 (17, [M + 1]^+^), 209 (100, [M (^35^Cl)]^+^), 208 (18), 175 (15), 174 (36), 146 (68), 128 (17), 120 (18), 119 (59), 92 (32), 91 (15); HRMS (ESI+): *m*/*z* calcd for C_10_H_9_ClNO_2_^+^ [M + H]^+^ 210.0316, found 210.0313. Anal. Calcd for C_10_H_8_ClNO_2_ (209.63): C, 57.30; H, 3.85; N, 6.68%. Found: C, 57.18; H, 3.83; N, 6.61%.

**3-Chloro-3-phenylquinoline-2,4(1*H*,3*H*)-dione (4b).** Compound **4b** (26.08 g, 96.0 mmol, 96%) was prepared from **3b** (23.73 g, 100 mmol). Pale yellow needles, m.p. 182–185 °C (benzene), m.p. [16] 178–180 °C (ethanol); *R*_f_ = 0.57 (30% ethyl acetate in chloroform). ^1^H NMR (500 MHz, CDCl_3_) *δ* 7.04 (d, 1H, *J* = 8.0 Hz, H-8), 7.18 (ddd, 1H, *J* = 7.8, 7.4, 0.7 Hz, H-6), 7.33–7.39 (m, 3H, H‑3^C^, H‑4^C^, H‑5^C^), 7.51–7.54 (m, 2H, H-2^C^, H-6^C^), 7.55 (ddd, 1H, *J* = 7.3, 6.5, 1.5 Hz, H-7), 7.97 (dd, 1H, *J* = 7.8, 1.2 Hz, H-5), 9.82 (s, 1H, H-1); ^13^C NMR (126 MHz, CDCl_3_) *δ* 74.9 (C-3), 116.9 (C-8), 118.7 (C-4a), 124.7 (C-6), 127.4 (C-2^C^, C-6^C^), 129.1 (C-5), 129.2 (C-3^C^, C-5^C^), 129.8 (C-4^C^), 134.6 (C-1^C^), 137.0 (C-7), 139.4 (C-8a), 168.8 (C-2), 187.9 (C-4); IR (cm^−1^): *ν* 3201, 3138, 3082, 2992, 2926, 1716, 1680, 1613, 1595, 1485, 1365, 755, 743, 690; MS (EI) *m*/*z* (%): 273 (7, [M (^37^Cl)]^+^), 271 (21, [M (^35^Cl)]^+^), 238 (12), 237 (80), 236 (100), 218 (10), 120 (63), 119 (19), 92 (34), 89 (10), 77 (12), 76 (10), 65 (14), 63 (10); HRMS (ESI+): *m*/*z* calcd for C_15_H_11_ClNO_2_^+^ [M + H]^+^ 272.0473, found 272.0480. Anal. Calcd for C_15_H_10_ClNO_2_ (271.70): C, 66.31; H, 3.71; N, 5.16%. Found: C, 66.07; H, 3.62; N, 5.29%.

### 3.3. General Procedure for the Synthesis of 3-Azidoquinoline-2,4(1H,3H)-Diones 5 (Scheme 1)

To a stirred solution of the 3-chloroquinoline-2,4(1*H*,3*H*)-dione **4** (40 mmol) in DMF (200 mL), sodium azide (3.90 g, 60 mmol) was added in small portions during 10 min. The reaction mixture was stirred in darkness for additional 2 h and then poured into ice-water (1.5 L). The precipitated solid was filtered, washed with water and dried at 50 °C in darkness, which afforded product **5**, pure according to TLC and ^1^H NMR spectrum, which was crystallized from benzene.

**3-Azido-3-methylquinoline-2,4(1*H*,3*H*)-dione (5a).** Compound **5a** (8.47 g, 39.2 mmol, 98%) was prepared from **4a** (8.39 g, 40.0 mmol). Colorless needles, m.p. 158–161 °C (benzene, 87% yield of recrystallization); *R*_f_ = 0.30 (30% ethyl acetate in chloroform). ^1^H NMR (500 MHz, CDCl_3_) *δ* 1.86 (s, 3H, CH_3_), 7.11 (d, 1H, *J* = 8.0 Hz, H-8), 7.22 (dd, 1H, *J* = 7.4, 7.4 Hz, H-6), 7.60–7.67 (m, 1H, H-7), 7.98 (d, 1H, *J* = 7.3 Hz, H-5), 9.86 (s, 1H, H-1); ^13^C NMR (126 MHz, CDCl_3_) *δ* 23.6 (CH_3_), 70.0 (C-3), 116.9 (C-8), 118.0 (C-4a), 124.6 (C-6), 128.6 (C-5), 137.2 (C-7), 140.0 (C-8a), 171.6 (C-2), 191.7 (C-4); IR (cm^−1^): *ν* 3202, 3078, 3005, 2936, 2108, 1708, 1682, 1614, 1598, 1485, 1392, 1284, 1156, 755, 612; MS (EI) *m*/*z* (%): 217 (0.24, [M + 1]^+^), 216 (2, [M]^+^), 147 (15), 120 (11), 119 (100), 92 (35), 91 (11), 64 (12); HRMS (ESI+): *m*/*z* calcd for C_10_H_9_N_4_O_2_^+^ [M + H]^+^ 217.0720, found 217.0724. Anal. Calcd for C_10_H_8_N_4_O_2_ (216.20): C, 55.55; H, 3.73; N, 25.91%. Found: C, 55.44; H, 3.72; N, 25.98%.

**3-Azido-3-phenylquinoline-2,4(1*H*,3*H*)-dione (5b).** Compound **5b** (10.90 g, 39.2 mmol, 98%) was prepared from **4b** (10.87 g, 40.0 mmol). Colorless needles, m.p. 186–189 °C (benzene, 96% yield of recrystallization); m.p. [9] 173–181 °C (benzene); *R*_f_ = 0.33 (38% ethyl acetate in petroleum ether); ^1^H NMR (500 MHz, CDCl_3_) *δ* 6.98 (d, 1H, *J* = 8.1 Hz, H-8), 7.16 (dd, 1H, *J* = 7.6, 7.6 Hz, H-6), 7.38–7.43 (m, 3H, H-3^C^, H-4^C^, H-5^C^), 7.48–7.53 (m, 2H, H-2^C^, H-6^C^), 7.54 (ddd, 1H, *J* = 7.7, 7.7, 1.6 Hz, H-7), 7.93 (dd, 1H, *J* = 7.8, 1.6 Hz, H-5), 9.30 (s, 1H, H-1); ^13^C NMR (126 MHz, CDCl_3_) *δ* 78.0 (C-3), 116.7 (C-8), 119.5 (C-4a), 124.6 (C-6), 127.3 (C-2^C^, C-6^C^), 128.6 (C-5), 129.8 (C-3^C^, C-5^C^), 130.4 (C-4^C^), 132.6 (C-1^C^), 136.9 (C-7), 139.4 (C-8a), 170.2 (C-2), 189.9 (C-4); ^15^N NMR (51 MHz, CDCl_3_) *δ* 133.4 (N-1); IR (cm^−1^): *ν* 3244, 2105, 1718, 1705, 1685, 1611, 1484, 1356, 1256, 877, 773, 744, 702, 611, 525; MS (EI) *m*/*z* (%): 250 (7, [M − N_2_]^+^), 236 (8, [M − N_3_]^+^), 147 (28), 120 (14), 119 (100), 104 (15), 92 (32), 77 (10), 76 (10), 64 (14); HRMS (ESI+): *m*/*z* calcd for C_15_H_11_N_2_O_2_^+^ [M − N_2_ + H]^+^ 251.0815, found 251.0818. HRMS (ESI−): *m*/*z* calcd for C_15_H_9_N_4_O_2_^−^ [M − H]^−^ 277.0731, found 277.0732; calcd for C_15_H_9_N_2_O_2_^−^ [M − N_2_ − H]^−^ 249.0670, found 249.0671. Anal. Calcd for C_15_H_10_N_4_O_2_ (278.27): C, 64.74; H, 3.62; N, 20.13%. Found: C, 64.54; H, 3.56; N, 20.38%.

### 3.4. General Procedure for the Synthesis of 3-(1H-1,2,3-Triazol-1-yl)Quinoline-2,4(1H,3H)-Diones 1a–d by Employing CuSO_4_/Cu^0^/DMF Conditions (Table 2, Entries 1 and 3–5)

A solution of phenylacetylene (**6a**) (1.287 g, 12.6 mmol) or propargyl alcohol (**6b**) (706 mg, 12.6 mmol) in DMF (4 mL) was added dropwise to a vigorously stirred mixture of 3-azidoquinoline-2,4(1*H*,3*H*)-dione **5** (12 mmol), CuSO_4_⋅5H_2_O (300 mg, 1.2 mmol), granular copper (1.5 g, 24 mmol) and DMF (24 mL). The reaction mixture was stirred in darkness for 30 min. Afterward, (NH_4_)_2_CO_3_ (3.5 g, 36 mmol) and water (12 mL) were added to the resulting brown-black suspension and the stirring was continued for 10 min. The reaction mixture was subjected to column chromatography with silica gel (15 g, column diameter of 1 cm) as a stationary phase, and 10% ethanol in chloroform as a mobile phase. Combined fractions containing yellow eluate were washed with saturated aqueous NH_4_Cl (5 × 50 mL), dried (Na_2_SO_4_), and concentrated under reduced pressure to afford pure products **1a**–**d**, which were recrystallized from ethanol for analyses.

**3-Methyl-3-(4-phenyl-1*H*-1,2,3-triazol-1-yl)quinoline-2,4(1*H*,3*H*)-dione (1a).** Colorless solid, m.p. 217–219 °C (ethanol); *R*_f_ = 0.35 (30% ethyl acetate in chloroform); ^1^H NMR (500 MHz, DMSO-*d_6_*) *δ* 2.15 (s, 3H, CH_3_), 7.22–7.27 (m, 2H, H-6, H-8), 7.33–7.39 (m, 1H, H-4^B^), 7.45–7.50 (m, 2H, H-3^B^, H-5^B^), 7.73–7.79 (m, 1H, H-7), 7.83–7.90 (m, 3H, H-5, H-2^B^, H-6^B^), 8.89 (s, 1H, H-5^A^), 11.48 (s, 1H, H-1); ^13^C NMR (126 MHz, DMSO-*d_6_*) *δ* 23.1 (CH_3_), 72.2 (C-3), 117.0 (C-8), 117.4 (C-4a), 122.4 (C-5^A^), 123.5 (C-6), 125.1 (C-2^B^, C-6^B^), 127.7 (C-5), 128.0 (C-4^B^), 129.0 (C-3^B^, C-5^B^), 130.6 (C-1^B^), 137.3 (C-7), 141.6 (C-8a), 145.8 (C-4^A^), 168.5 (C-2), 190.7 (C-4); IR (cm^−1^): *ν* 3137, 2911, 1714, 1683, 1612, 1483, 1430, 1386, 1355, 1238, 1023, 808, 759, 690, 594; MS (EI) *m*/*z* (%): 319 (2, [M + 1]^+^), 318 (8, [M]^+^), 117 (14), 116 (100), 102 (12), 89 (14); HRMS (ESI+): *m*/*z* calcd for C_18_H_15_N_4_O_2_^+^ [M + H]^+^ 319.1190, found 319.1188. Anal. Calcd for C_18_H_14_N_4_O_2_ (318.33): C, 67.91; H, 4.43; N, 17.60%. Found: C, 67.80; H, 4.47; N, 17.89%.

**3-Phenyl-3-(4-phenyl-1*H*-1,2,3-triazol-1-yl)quinoline-2,4(1*H*,3*H*)-dione (1b).** Colorless solid, m.p. 280–283 °C (ethanol); m.p. [9] 274–277 °C (ethanol); *R*_f_ = 0.37 (30% ethyl acetate in chloroform); ^1^H NMR (500 MHz, DMSO-*d_6_*) *δ* 7.12 (d, 1H, *J* = 8.1 Hz, H-8), 7.16–7.20 (m, 1H, H-6), 7.31–7.37 (m, 1H, H-4^B^), 7.40–7.47 (m, 4H, H-3^B^, H-5^B^, H-2^C^, H-6^C^), 7.49–7.55 (m, 3H, H-3^C^, H-4^C^, H-5^C^), 7.62–7.67 (m, 1H, H-7), 7.80–7.84 (m, 2H, H-2^B^, H-6^B^), 7.86 (dd, 1H, *J* = 7.8, 1.4 Hz, H-5), 8.49 (s, 1H, H-5^A^), 11.68 (s, 1H, H-1); ^13^C NMR (126 MHz, DMSO-*d_6_*) *δ* 80.0 (C-3), 116.7 (C-8), 119.2 (C-4a), 123.4 (C-5^A^), 123.5 (C-6), 125.2 (C-2^B^, C-6^B^), 127.6 (C-5), 128.0 (C-4^B^), 128.9 (C-2^C^, C-6^C^), 129.0 (C-3^B^, C-5^B^), 129.6 (C-3^C^, C-5^C^), 129.9 (C-1^C^), 130.5 (C-1^B^), 130.6 (C-4^C^), 137.0 (C-7), 140.5 (C-8a), 145.3 (C-4^A^), 166.8 (C-2), 188.9 (C-4); IR (cm^−1^): *ν* 3275, 3169, 1721, 1690, 1613, 1595, 1486, 1452, 1353, 854, 771, 756, 699, 666, 607; MS (EI) *m*/*z* (%): 381 (2, [M + 1]^+^), 380 (8, [M]^+^), 247 (13), 237 (15), 236 (56), 220 (13), 218 (13), 120 (11), 117 (10), 116 (100), 102 (15), 92 (10), 89 (15), 77 (14); HRMS (ESI+): *m*/*z* calcd for C_23_H_17_N_4_O_2_^+^ [M + H]^+^ 381.1346, found 381.1341. Anal. Calcd for C_23_H_16_N_4_O_2_ (380.40): C, 72.62; H, 4.24; N, 14.73%. Found: C, 72.40; H, 4.23; N, 14.90%.

**3-(4-(Hydroxymethyl)-1*H*-1,2,3-triazol-1-yl)-3-methylquinoline-2,4(1*H*,3*H*)-dione (1c).** Colorless solid, m.p. 188–189 °C (ethanol); *R*_f_ = 0.35 (30% ethyl acetate in chloroform); ^1^H NMR (500 MHz, DMSO-*d*_6_) *δ* 2.08 (s, 3H, CH_3_), 4.55 (d, 2H, *J* = 5.6 Hz, CH_2_), 5.28 (t, 1H, *J* = 5.6 Hz, OH), 7.18–7.25 (m, 2H, H-6, H-8), 7.69–7.76 (m, 1H, H-7), 7.83 (dd, 1H, *J* = 8.1, 1.4 Hz, H-5), 8.26 (s, 1H, H-5^A^), 11.39 (s, 1H, H-1); ^13^C NMR (126 MHz, DMSO-*d*_6_) *δ* 23.1 (CH_3_), 55.0 (CH_2_), 71.9 (C-3), 116.9 (C-8), 117.5 (C-4a), 123.3 (C-6), 123.7 (C-5^A^), 127.6 (C-5), 137.1 (C-7), 141.6 (C-8a), 147.4 (C-4^A^), 168.7 (C-2), 190.8 (C-4); IR (cm^−1^): *ν* 3148, 2992, 2919, 1729, 1682, 1613, 1486, 1378, 1345, 1235, 1189, 1009, 751, 667, 590; MS (EI) *m*/*z* (%): 273 (2, [M + 1]^+^), 272 (13, [M]^+^), 185 (68), 175 (89), 174 (45), 146 (100), 128 (58), 120 (70), 119 (75), 92 (66), 91 (39), 77 (39), 65 (37), 55 (39), 42 (79); HRMS (ESI+): *m*/*z* calcd for C_13_H_13_N_4_O_3_^+^ [M + H]^+^ 273.0982, found 273.0981. Anal. Calcd for C_13_H_12_N_4_O_3_ (272.26): C, 57.35; H, 4.44; N, 20.58%. Found: C, 57.20; H, 4.42; N, 20.83%.

**3-(4-(Hydroxymethyl)-1*H*-1,2,3-triazol-1-yl)-3-phenylquinoline-2,4(1*H*,3*H*)-dione dimethylformamide solvate (1d·DMF).** Colorless solid, m.p. 139–143 °C (ethanol); m.p. [9] 116–135 °C (benzene); *R*_f_ = 0.27 (10% ethanol in chloroform); ^1^H NMR (500 MHz, DMSO-*d*_6_) *δ* 4.53 (d, 2H, *J* = 5.7 Hz, CH_2_), 5.22 (t, 1H, *J* = 5.7 Hz, OH), 7.09 (d, 1H, *J* = 8.1 Hz, H-8), 7.13–7.18 (m, 1H, H-6), 7.36–7.42 (m, 2H, H-2^C^, H-6^C^), 7.47–7.53 (m, 3H, H-3^C^, H-4^C^, H-5^C^), 7.59–7.65 (m, 1H, H-7), 7.77 (s, 1H, H-5^A^), 7.83 (dd, 1H, *J* = 7.8, 1.3 Hz, H-5), 11.60 (s, 1H, H-1); ^13^C NMR (126 MHz, DMSO-*d*_6_) *δ* 55.0 (CH_2_), 79.7 (C-3), 116.7 (C-8), 119.2 (C-4a), 123.4 (C-6), 124.8 (C-5^A^), 127.5 (C-5), 128.8 (C-2^C^, C-6^C^), 129.6 (C-3^C^, C-5 ^C^), 130.2 (C-1^C^), 130.5 (C-4^C^), 136.9 (C-7), 140.6 (C-8a), 146.8 (C-4^A^), 166.8 (C-2), 189.0 (C-4); IR (cm^−1^): *ν* 3392, 3136, 2926, 1724, 1692, 1654, 1613, 1485, 1438, 1353, 857, 769, 752, 665, 603; MS (EI) *m*/*z* (%): 335 (0.8, [M + 1]^+^), 334 (4, [M]^+^), 305 (37), 275 (18), 249 (30), 247 (27), 237 (50), 236 (100), 218 (35), 208 (18), 180 (20), 120 (33), 104 (23), 92 (23), 77 (34); HRMS (ESI+): *m*/*z* calcd for C_18_H_15_N_4_O_3_^+^ [M + H]^+^ 335.1139, found 335.1138. Anal. Calcd for C_21_H_21_N_5_O_4_ (407.42): C, 61.91; H, 5.20; N, 17.19%. Found: C, 61.89; H, 5.24; N, 17.28%.

### 3.5. Synthesis of Compound ***1a*** by Employing CuSO_4_∙5H_2_O/l-Ascorbic Acid/CH_2_Cl_2_/Water Conditions (Table 2, Entry 2)

To a solution of azide **5a** (162 mg, 0.75 mmol) and phenylacetylene (**6a**) (153 mg, 1.5 mmol) in dichloromethane (8 mL) a solution of l-ascorbic acid (106 mg, 0.60 mmol) in water (4 mL), and a solution of CuSO_4_∙5H_2_O (15 mg, 0.06 mmol) in water (4mL) were added. The two-phase reaction mixture was stirred in darkness at room temperature for 48 h. The reaction mixture was extracted with chloroform (5 × 30 mL). The combined organic layers were dried (Na_2_SO_4_), filtered, and evaporated to dryness. The residue was dissolved in chloroform (5 mL) and subjected to silica gel (30 g) column chromatography using 38% ethyl acetate in hexane as eluent, affording compound **1a** (199 mg, 63 mmol 83%).

### 3.6. General Procedure for the Synthesis of (1-(2,4-Dioxo-1,2,3,4-Tetrahydroquinolin-3-yl)-1H-1,2,3-Triazol-4-yl)Methyl Acetates 1e,f (Scheme 2)

Acetic anhydride (12 mL, 12.9 g, 126 mmol) was added to a light yellow solution of compound **1c** or **1d** (6 mmol) in pyridine (18 mL) under stirring during 2 min. The reaction mixture was stirred for 1 h, followed by evaporation of volatiles under reduced pressure. The remaining pyridine was removed by co-distillation with ethanol (6 × 40 mL). The residue was triturated with water (300 mL) to form a white precipitate which was collected by filtration on a sintered glass filter with suction, washed with water to neutral and dried to give acetates **1e** or **1f**. The crude product was recrystallized from the solvent indicated below.

**(1-(3-Methyl-2,4-dioxo-1,2,3,4-tetrahydroquinolin-3-yl)-1*H*-1,2,3-triazol-4-yl)methyl acetate (1e).** Compound **1e** (1.58 g, 5.04 mmol, 84%) was prepared from **1c** (1.63 g, 6.0 mmol). Pale yellow solid, m.p. 145–148 °C (ethyl acetate); *R*_f_ = 0.33 (5% ethanol in chloroform); ^1^H NMR (500 MHz, DMSO-*d*_6_) *δ* 2.06 (s, 3H, COCH_3_), 2.09 (s, 3H, C-3–CH_3_), 5.16 (s, 2H, CH_2_), 7.19–7.26 (m, 2H, H-6, H-8), 7.70–7.77 (m, 1H, H-7), 7.83 (dd, 1H, *J* = 8.0, 1.4 Hz, H-5), 8.45 (s, 1H, H-5^A^), 11.40 (s, 1H, H-1); ^13^C NMR (126 MHz, DMSO-*d*_6_) *δ* 20.6 (COCH_3_), 23.2 (C-3–CH_3_), 57.1 (CH_2_), 72.4 (C-3), 116.9 (C-8), 117.5 (C-4a), 123.3 (C-6), 125.8 (C-5^A^), 127.6 (C-5), 137.1 (C-7), 141.4 (C-4^A^), 141.6 (C-8a), 168.6 (C-2), 170.1 (COCH_3_), 190.7 (C-4); ^15^N NMR (51 MHz, DMSO-d_6_) *δ* 133.5 (N-1), 248.7 (N-1^A^), 354.1 (N-3^A^), 362.9 (N-2^A^); IR (cm^−1^): *ν* 3467, 3249, 3148, 2920, 1722, 1685, 1613, 1485, 1439, 1384, 1355, 1239, 1028, 759, 666; MS (EI) *m*/*z* (%): 315 (2, [M + 1]^+^), 314 (11, [M]^+^), 244 (22), 201 (22), 175 (71), 174 (31), 146 (43), 128 (26), 120 (25), 119 (27), 92 (24), 55 (20), 43 (100), 42 (26); HRMS (ESI+): *m*/*z* calcd for C_15_H_15_N_4_O_4_^+^ [M + H]^+^ 315.1088, found 315.1087. Anal. Calcd for C_15_H_14_N_4_O_4_ (314.30): C, 57.32; H, 4.49; N, 17.83%. Found: C, 57.32; H, 4.59; N, 17.58%.

**(1-(2,4-Dioxo-3-phenyl-1,2,3,4-tetrahydroquinolin-3-yl)-1*H*-1,2,3-triazol-4-yl)methyl acetate (1f).** Compound **1f** (1.92 g, 5.1 mmol, 85%) was prepared from **1d** (2.01 g, 6.0 mmol). Colorless crystals, m.p. 130–134 °C (ethanol, 80% yield of recrystallization); *R*_f_ = 0.40 (5% ethanol in chloroform); ^1^H NMR (500 MHz, DMSO-*d*_6_) *δ* 2.04 (s, 3H, CH_3_), 5.13 (s, 2H, CH_2_), 7.09 (d, 1H, *J* = 8.1 Hz, H-8), 7.13–7.18 (m, 1H, H-6), 7.35–7.42 (m, 2H, H-2^C^, H-6^C^), 7.46–7.54 (m, 3H, H-3^C^, H-4^C^, H-5^C^), 7.59–7.65 (m, 1H, H-7), 7.83 (dd, 1H, *J* = 7.8, 1.3 Hz), 8.07 (s, 1H, H-5^A^), 11.62 (s, 1H, H-1); ^13^C NMR (126 MHz, DMSO-*d*_6_) *δ* 20.7 (CH_3_), 57.1 (CH_2_), 79.9 (C-3), 116.7 (C-8), 119.3 (C-4a), 123.4 (C-6), 127.0 (C-5^A^), 127.5 (C-5), 128.8 (C-2^C^, C-6^C^), 129.6 (C-3^C^, C-5^C^), 130.0 (C-1^C^), 130.6 (C-4^C^), 136.9 (C-7), 140.5 (C-8a), 140.8 (C-4^A^), 166.8 (C-2), 170.1 (COCH_3_), 188.8 (C-4); IR (cm–1): *ν* 3501, 3155, 2920, 1722, 1707, 1686, 1614, 1594, 1484, 1358, 1252, 1229, 1063, 857, 759; MS (EI) *m*/*z* (%): 377 (1, [M + 1]+), 376 (6, [M]+), 306 (16), 289 (18), 288 (54), 263 (15), 237 (50), 236 (100), 218 (34), 180 (14), 141 (14), 120 (24), 92 (14), 77 (19), 43 (16); HRMS (ESI+): *m*/*z* calcd for C_20_H_17_N_4_O_4_^+^ [M + H]^+^ 377.1244, found 377.1241.

### 3.7. General Procedure for the Synthesis of 3-(1H-1,2,3-Triazol-1-yl)-1-(prop-2-yn-1-yl)Quinoline-2,4(1H,3H)-Diones 7 (Table 3)

The mixture of the appropriate compound **1a**,**b**,**e**,**f** (8.00 mmol), potassium carbonate (3.32 g, 24 mmol), and DMF (40 mL) was stirred for 40 min. During this time, the original yellow color of the suspension changed to orange. Afterwards, under continued stirring, an 80% solution of propargyl bromide (**6c**) in toluene (1.78 g, 12 mmol) diluted with DMF (20 mL) was added dropwise during 1 min and stirring was continued for 90 min. Then the mixture was reduced *in vacuo* and then toluene (50 mL) was added and the whole evaporated *in vacuo* at 50 °C. This was repeated seven times in order to remove traces of DMF. The residual light brown solid was suspended in chloroform (100 mL) and the suspension was acidified with 0.5 m HCl, whereas carbon dioxide was evolved owing to decomposition of unreacted potassium carbonate. The formed emulsion was diluted with water, organic phase was separated and aqueous phase was extracted with chloroform (5 × 40 mL). The organic phases were combined, dried (Na_2_SO_4_), filtered and taken down *in vacuo*. The residual solid TLC pure product was crystallized from a suitable solvent. The yields of prepared compounds **7** are given in Table 3.

**3-Methyl-3-(4-phenyl-1*H*-1,2,3-triazol-1-yl)-1-(prop-2-yn-1-yl)quinoline-2,4(1*H*,3*H*)-dione (7a).** Colorless crystals, m.p. 187–189 °C (benzene); *R*_f_ = 0.63 (30% ethyl acetate in chloroform); ^1^H NMR (500 MHz, DMSO-*d*_6_) *δ* 2.16 (s, 3H, CH_3_), 3.39 (dd, 1H, *J* = 2.3, 2.3 Hz, C≡CH), 4.90 (dd, 1H, *J* = 18.1, 2.3 Hz, N-1–CH*α*), 4.97 (dd, 1H, *J* = 18.1, 2.3 Hz, N-1–CH*β*), 7.34–7.42 (m, 2H, H-6, H-4^B^), 7.48 (dd, 2H, *J* = 7.7, 7.7 Hz, H-3^B^, H-5^B^), 7.61 (d, 1H, *J* = 8.4 Hz, H-8), 7.84–7.89 (m, 2H, H-2^B^, H-6^B^), 7.89–7.95 (m, 1H, H-7), 8.00 (dd, *J* = 7.7, 1.5 Hz, H-5), 8.89 (s, 1H, H-5^A^); ^13^C NMR (126 MHz, DMSO-*d*_6_) *δ* 23.3 (CH_3_), 32.7 (N-1–CH_2_), 72.6 (C-3), 75.4 (C≡*C*H), 78.2 (*C*≡CH), 116.7 (C-8), 119.0 (C-4a), 122.5 (C-5^A^), 124.2 (C-6), 125.1 (C-2^B^, C-6^B^), 128.1 (C-4^B^), 128.2 (C-5), 129.0 (C-3^B^, C-5^B^), 130.5 (C-1^B^), 137.3 (C-7), 140.8 (C-8a), 145.9 (C-4^A^), 167.7 (C-2), 189.7 (C-4); IR (cm^−1^): *ν* 3261, 3173, 2122, 1713, 1678, 1601, 1469, 1427, 1381, 1368, 1353, 1306, 1189, 769, 754; MS (EI) *m*/*z* (%): 357 (2, [M + 1]^+^), 356 (8, [M]^+^), 259 (10), 128 (11), 117 (16), 116 (100), 102 (17), 90 (11), 89 (16), 77 (10), 76 (10); HRMS (ESI+): *m*/*z* calcd for C_21_H_17_N_4_O_2_^+^ [M + H]^+^ 357.1346, found 357.1342. Anal. Calcd for C_21_H_16_N_4_O_2_ (356.38): C, 70.77; H, 4.53; N, 15.72%. Found: C, 70.81; H, 4.58; N, 15.82%.

**3-Phenyl-3-(4-phenyl-1*H*-1,2,3-triazol-1-yl)-1-(prop-2-yn-1-yl)quinoline-2,4(1*H*,3*H*)-dione (7b).** Colorless crystals, m.p. 232–234 °C (ethanol); *R*_f_ = 0.69 (30% ethyl acetate in chloroform); ^1^H NMR (500 MHz, CDCl_3_) *δ* 2.34 (dd, 1H, *J* = 2.4, 2.4 Hz), 4.51 (dd, 1H, *J* = 17.8, 2.3 Hz), 5.37 (dd, 1H, *J* = 17.8, 2.3 Hz), 7.23 (dd, 1H, *J* = 7.6, 7.6 Hz), 7.26 (s, 1H), 7.27–7.31 (m, 1H), 7.32–7.40 (m, 3H), 7.43–7.51 (m, 3H), 7.51–7.56 (m, 2H), 7.62–7.69 (m, 1H), 7.73–7.81 (m, 2H), 8.05 (dd, 1H, *J* = 7.7, 1.4 Hz); ^13^C NMR (126 MHz, CDCl_3_) *δ* 33.6, 73.6, 76.9, 79.6, 115.8, 121.0, 122.3, 124.6, 126.0, 128.1, 128.8, 129.0, 129.2, 130.0, 130.1, 130.7, 131.3, 136.9, 140.6, 146.0, 165.8, 187.5; IR (cm^−1^): *ν* 3197, 2983, 2118, 1716, 1680, 1603, 1468, 1448, 1304, 1039, 870, 760, 752, 694; MS (EI) *m*/*z* (%): 419 (13, [M + 1]^+^), 418 (75, [M]^+^), 390 (43), 287 (22), 286 (31), 285 (89), 276 (25), 275 (100), 274 (28), 259 (70), 248 (46), 235 (53), 145 (52), 116 (95), 44 (99); HRMS (ESI+): *m*/*z* calcd for C_26_H_19_N_4_O_2_^+^ [M + H]^+^ 419.1503, found 419.1502. Anal. Calcd for C_26_H_18_N_4_O_2_: C, 74.63; H, 4.34; N, 13.39%. Found: C, 74.45; H, 4.40; N, 13.43%.

**(1-(3-Methyl-2,4-dioxo-1-(prop-2-yn-1-yl)-1,2,3,4-tetrahydroquinolin-3-yl)-1*H*-1,2,3-triazol-4-yl)methyl acetate (7c).** Colorless crystals, m.p. 159–161 °C (ethyl acetate); *R*_f_ = 0.29 (30% ethyl acetate in chloroform); ^1^H NMR (500 MHz, DMSO-*d*_6_) *δ* 2.06 (s, 3H, COCH_3_), 2.10 (s, 3H, C-3–CH_3_), 3.37 (dd, 1H, *J* = 2.4, 2.4 Hz, C≡CH), 4.84 (dd, 1H, *J* = 18.1, 2.4 Hz, N-1–CH*α*), 4.95 (dd, 1H, *J* = 18.1, 2.4 Hz, N-1–CH*β*), 5.17 (s, 2H, OCH_2_), 7.37 (dd, 1H, *J* = 7.5, 7.5 Hz, H-6), 7.58 (d, 1H, *J* = 8.4 Hz, H-8), 7.87–7.93 (m, 1H, H-7), 7.96 (dd, 1H, *J* = 7.7, 1.5 Hz, H-5), 8.46 (s, 1H, H-5^A^); ^13^C NMR (126 MHz, DMSO-*d*_6_) *δ* 20.6 (CO*C*H_3_), 23.4 (C-3–CH_3_), 32.6 (N-1–CH_2_), 57.1 (OCH_2_), 72.8 (C-3), 75.3 (C≡*C*H), 78.2 (*C*≡CH), 116.6 (C-8), 119.2 (C-4a), 124.0 (C-6), 126.0 (C-5^A^), 128.0 (C-5), 137.1 (C-7), 140.7 (C-8a), 141.5 (C-4^A^), 167.8 (C-2), 170.1 (*C*OCH_3_), 189.63 (C-4); ^15^N NMR (51 MHz, DMSO-*d*_6_) *δ* 134.4 (N1), 247.9 (N-1^A^), 354.0 (N-3^A^), 363.4 (N-2^A^); IR (cm^−1^): *ν* 3256, 3152, 2122, 1721, 1687, 1604, 1471, 1383, 1306, 1246, 1194, 1053, 1008, 756; MS (EI) *m*/*z* (%): 353 (3, [M + 1]^+^), 352 (12, [M]^+^), 213 (69), 212 (34), 184 (19), 156 (32), 146 (17), 130 (19), 129 (21), 128 (22), 77 (17), 57 (16), 55 (23), 43 (100), 42 (17); HRMS (ESI+): *m*/*z* calcd for C_18_H_17_N_4_O_4_^+^ ([M+H]^+^): 353.1244, found 353.1246. Anal. Calcd for C_18_H_16_N_4_O_4_ (352.34): C, 61.36; H, 4.58; N, 15.90%. Found: C, 61.27; H, 4.64; N, 15.87%.

**(1-(2,4-Dioxo-3-phenyl-1-(prop-2-yn-1-yl)-1,2,3,4-tetrahydroquinolin-3-yl)-1*H*-1,2,3-triazol-4-yl)methyl acetate (7d).** Colorless crystals, m.p. 210–214 °C; *R*_f_ = 0.66 (5% ethanol in chloroform); ^1^H NMR (500 MHz, DMSO-*d*_6_) *δ* 2.05 (s, 3H, COCH_3_), 3.41 (dd, 1H, *J* = 2.4, 2.3 Hz, C≡CH), 4.80 (dd, 1H, *J* = 18.0, 2.3 Hz, N-1–CH*α*), 5.09–5.20 (m, 3H, N-1–CH*β*, OCH_2_), 7.24–7.32 (m, 3H, H-6, H-2^C^, H-6^C^), 7.41–7.51 (m, 4H, H-8, H-3^C^, H-4^C^, H-5^C^), 7.73–7.79 (m, 1H, H-7), 7.92 (dd, 1H, *J* = 7.7, 1.5 Hz, H-5), 8.15 (s, 1H, H-5^A^); ^13^C NMR (126 MHz, DMSO-*d*_6_) *δ* 20.6 (CO*C*H_3_), 33.1 (N-1–CH_2_), 57.1 (OCH_2_), 75.5 (C≡*C*H), 77.9 (*C*≡CH), 80.0 (C-3), 116.3 (C-8), 120.9 (C-4a), 124.2 (C-6), 127.1 (C-5^A^), 127.8 (C-5), 128.6 (C-2^C^, C-6^C^), 129.5 (C-3^C^, C-5^C^), 129.9 (C-1^C^), 130.7 (C-4^C^), 136.7 (C-7), 140.0 (C-8a), 140.9 (C-4^A^), 165.8 (C-2), 170.1 (*C*OCH_3_), 187.7 (C-4); IR (cm^−1^): *ν* 3227, 3152, 2116, 1736, 1715, 1683, 1602, 1467, 1379, 1303, 1251, 1036, 764, 747, 694; MS (EI) *m*/*z* (%): 415 (2, [M + 1]^+^), 414 (7, [M]^+^), 313 (26), 275 (72), 274 (63), 246 (28), 235 (31), 218 (29), 217 (30), 156 (26), 130 (29), 105 (22), 104 (29), 103 (22), 43 (100); HRMS (ESI+): *m*/*z* calcd for C_23_H_19_N_4_O_4_^+^ [M + H]^+^ 415.1401, found 415.1403. Anal. Calcd for C_23_H_18_N_4_O_4_: C, 66.66; H, 4.38; N, 13.52%. Found: C, 66.45; H, 4.39; N, 13.35%.

### 3.8. General Procedure for the Synthesis of Bis-Triazoles 2a,b,d,e,g,h,j,k by Employing CuSO_4_/Cu^0^/DMF Conditions (Table 4, Entries 1, 2, 4, 8, 10, 11, 13 and 14)

A solution of azidobenzene (**8b**, 197 mg, 1.65 mmol) or (azidomethyl)benzene (**8a**, 220 mg, 1.65 mmol) in DMF (4 mL) was added to a vigorously stirred mixture of the appropriate *N*‑propargylquinoline-2,4(1*H*,3*H*)-dione **7** (1.5 mmol), CuSO_4_∙5H_2_O (38 mg, 0.15 mmol) and granular copper (191 mg, 3.05 mmol) in DMF (5 mL). The reaction mixture was stirred in darkness at room temperature for the time given in Table 4. The color of the mixture became brown-black. Then, (NH_4_)_2_CO_3_ (432 mg, 4.5 mmol) and water (2 mL) were added to the reaction mixture and the stirring was continued for 10 min. The reaction mixture was poured into a narrow (1 cm in diameter) column of silica gel (15 g). The organic portion was eluted with 10% ethanol in chloroform (approximately 150 mL). The yellow eluate was washed with saturated aqueous NH_4_Cl (50 mL), dried over anhydrous sodium sulfate, filtered, and the solvent was removed by rotary evaporation *in vacuo*. The TLC pure product thus prepared, with the exception of compounds **2d**,**e**,**k**, was crystallized from suitable solvent. The yields of prepared compounds **2** are given in Table 4.

**1-((1-Benzyl-1*H*-1,2,3-triazol-4-yl)methyl)-3-methyl-3-(4-phenyl-1*H*-1,2,3-triazol-1-yl)quinoline-2,4(1*H*,3*H*)-dione (2a).** Colorless crystals, m.p. 202–204 °C (ethanol); *R*_f_ = 0.40 (30% ethyl acetate in chloroform); ^1^H NMR (500 MHz, DMSO-*d*_6_) *δ* 2.18 (s, 3H, CH_3_), 5.24 (d, 1H, *J* = 16.2 Hz, N-1–CH*α*), 5.49 (d, 1H, *J* = 16.2 Hz, N-1–CH*β*), 5.58 (s, 2H, N-1^D^–CH_2_), 7.24–7.29 (m, 2H, H-2^E^, H-6^E^), 7.29–7.40 (m, 5H, H-6, H-4^B^, H-3^E^, H-4^E^, H-5^E^), 7.49 (dd, 2H, *J* = 7.7, 7.7 Hz, H-3^B^, H-5^B^), 7.67 (d, 1H, *J* = 8.5 Hz, H-8), 7.79–7.90 (m, 3H, H-7, H-2^B^, H-6^B^), 7.96 (dd, 1H, *J* = 7.7, 1.4 Hz, H-5), 8.16 (s, 1H, H-5^D^), 8.87 (s, 1H, H-5^A^); ^13^C NMR (126 MHz, DMSO-*d*_6_) *δ* 23.4 (CH_3_), 38.7 (N-1–*C*H_2_), 52.8 (N-1^D^–*C*H_2_), 72.8 (C-3), 116.7 (C-8), 119.1 (C-4a), 122.5 (C-5^A^), 123.8 (C-5^D^), 123.9 (C-6), 125.1 (C-2^B^, C-6^B^), 127.9 (C-2^E^, C-6^E^), 128.0 (C-4^B^), 128.1 (C-5), 128.1 (C-4^E^), 128.7 (C-3^E^, C-5^E^), 129.1 (C-3^B^, C-5^B^), 130.6 (C-1^B^), 136.0 (C-1^E^), 137.2 (C-7), 141.5 (C-8a), 142.2 (C-4^D^), 145.9 (C-4^A^), 168.2 (C-2), 190.0 (C-4); IR (cm^−1^): *ν* 3137, 3128, 1711, 1673, 1600, 1471, 1387, 1051, 768, 761, 718, 694; MS (EI) *m*/*z* (%): 490 (2, [M + 1]^+^), 489 (6, [M]^+^), 289 (13), 145 (17), 144 (16), 117 (11), 116 (44), 91 (100), 90 (10), 89 (12); HRMS (ESI+): *m*/*z* calcd for C_28_H_24_N_7_O_2_^+^ [M + H]^+^ 490.1986, found 490.1981. Anal. Calcd for C_28_H_23_N_7_O_2_ (489.53) C, 68.70; H, 4.74; N, 20.03. Found: C, 68.71; H, 4.78; N, 20.36.

**3-Methyl-3-(4-phenyl-1*H*-1,2,3-triazol-1-yl)-1-((1-phenyl-1*H*-1,2,3-triazol-4-yl)methyl)quinoline-2,4(1*H*,3*H*)-dione (2b).** Colorless crystals, m.p. 194–197 °C (benzene); *R*_f_ = 0.48 (30% ethyl acetate in chloroform); ^1^H NMR (500 MHz, DMSO-*d*_6_) *δ* 2.23 (s, 3H, CH_3_), 5.33 (d, 1H, *J* = 16.4 Hz, N-1–CH*α*), 5.62 (d, 1H, *J* = 16.4 Hz, N-1–CH*β*), 7.31–7.39 (m, 2H, H-6, H-4^B^), 7.45–7.52 (m, 3H, H-3^B^, H-5^B^, H-4^E^), 7.55–7.62 (m, 2H, H-3^E^, H-5^E^), 7.70 (d, 1H, *J* = 8.5 Hz, H-8), 7.82–7.91 (m, 5H, H-7, H‑2^B^, H-6^B^, H-2^E^, H-6^E^), 7.98 (dd, 1H, *J* = 7.7, 1.5 Hz, H-5), 8.75 (s, 1H, H-5^D^), 8.87 (s, 1H, H-5^A^); ^13^C NMR (126 MHz, DMSO-*d*_6_) *δ* 23.4 (CH_3_), 38.7 (N-1–*C*H_2_), 73.0 (C-3), 116.8 (C-8), 119.2 (C-4a), 120.2 (C-2^E^, C-6^E^), 121.8 (C-5^D^), 122.5 (C-5^A^), 124.0 (C-6), 125.2 (C-2^B^, C-6^B^), 128.1 (C-4^B^), 128.1 (C-5), 128.8 (C-4^E^), 129.1 (C-3^B^, C-5^B^), 129.9 (C-3^E^, C-5^E^), 130.6 (C-1^B^), 136.5 (C-1^E^), 137.3 (C-7), 141.6 (C-8a), 143.3 (C-4^D^), 146.0 (C-4^A^), 168.3 (C-2), 190.0 (C-4); ^15^N NMR (51 MHz, DMSO-*d*_6_) *δ* 136.3 (N1), 248.9 (N-1^A^), 255.7 (N-D-1), 347.1 (N-3^A^), 353.4 (N-3^D^), 358.1 (N-2^D^), 363.2 (N-2^A^); IR (cm^−1^): *ν* 3275, 1721, 1690, 1613, 1485, 1353, 854, 771, 756, 698, 666, 607, 520; MS (EI) *m*/*z* (%): 476 (3, [M + 1]^+^), 475 (8, [M]^+^), 289 (14), 145 (12), 131 (11), 130 (100), 129 (18), 128 (11), 116 (56), 104 (12), 103 (16), 102 (12), 89 (12), 77 (69); HRMS (ESI+): *m*/*z* calcd for C_27_H_22_N_7_O_2_^+^ [M + H]^+^ 476.1829, found 476.1825. Anal. Calcd for C_27_H_21_N_7_O_2_ (475.50): C, 68.20; H, 4.45; N, 20.62%. Found: C, 68.48; H, 4.53; N, 20.60%.

**(1-(1-((1-Benzyl-1*H*-1,2,3-triazol-4-yl)methyl)-3-methyl-2,4-dioxo-1,2,3,4-tetrahydroquinolin-3-yl)-1*H*-1,2,3-triazol-4-yl)methyl acetate (2d).** Colorless powder, m.p. 69–82 °C; *R*_f_ = 0.42 (30% ethyl acetate in chloroform); ^1^H NMR (500 MHz, CDCl_3_), *δ* 2.09 (s, 3H, COCH_3_), 2.12 (s, 3H, C-3–CH_3_), 5.25 (s, 2H, OCH_2_), 5.33 (s, 2H, N-1-CH_2_), 5.45 (d, 1H, *J* = 14.8 Hz, N-1^D^–CH*α*), 5.51 (d, 1H, *J* = 14.8 Hz, N-1^D^–CH*β*), 7.23–7.26 (m, 3H, H-6, H-2^E^, H-6^E^), 7.32–7.38 (m, 3H, H-3^E^, H-4^E^, H-5^E^), 7.55 (s, 1H, H-5^D^), 7.73 (ddd, 1H, *J* = 8.7, 7.1, 1.6 Hz, H-7), 7.78 (s, 1H, H-5^A^), 7.82 (d, 1H, *J* = 8.4 Hz, H-8), 8.02 (dd, 1H, *J* = 7.7, 1.6 Hz, H-5); ^13^C NMR (126 MHz, CDCl_3_) *δ* 21.1 (CO*C*H_3_), 23.5 (C-3–*C*H_3_), 39.5 (N-1–CH_2_), 54.5 (N-1^D^–CH_2_), 57.7 (OCH_2_), 71.6 (C-3), 116.9 (C-8), 119.2 (C-4a), 123.5 (C-5^D^), 124.2 (C-5^A^), 124.6 (C-6), 128.3 (C-2^E^, C-6^E^), 129.0 (C-4^E^), 129.3 (C-3^E^, C-5^E^), 129.3 (C-5), 134.4 (C-1^E^), 137.8 (C-7), 141.7 (C-8a), 142.3 (C-4^A^), 142.9 (C-4^D^), 168.2 (C-2), 171.1 (*C*OCH_3_), 189.4 (C-4); ^15^N NMR (51 MHz, CDCl_3_) *δ* 138.7 (N-1), 248.4 (N-1^A^), 250.4 (N-1^D^), 350.0 (N-3^D^), 355.2 (N-3^A^), 361.6 (N-2^A^), 362.6 (N-2^D^); IR (cm^−1^): *ν* 3143, 2930, 1739, 1717, 1679, 1602, 1470, 1384, 1243, 1186, 1050, 1028, 765, 721, 664; MS (EI) *m*/*z* (%): 486 (0.3, [M + 1]^+^), 485 (1, [M]^+^), 144 (18), 91 (100), 43 (24); HRMS (ESI+): *m*/*z* calcd for C_25_H_24_N_7_O_4_^+^ [M + H]^+^ 486.1884, found 486.1884.

**(1-(3-Methyl-2,4-dioxo-1-((1-phenyl-1*H*-1,2,3-triazol-4-yl)methyl)-1,2,3,4-tetrahydroquinolin-3-yl)-1*H*-1,2,3-triazol-4-yl)methyl acetate (2e).** Colorless powder, m.p. 78–97 °C; *R*_f_ = 0.25 (30% ethyl acetate in chloroform); ^1^H NMR (500 MHz, CDCl_3_) *δ* 2.10 (s, 3H, COCH_3_), 2.20 (s, 3H, C-3–CH_3_), 5.27 (s, 2H, OCH_2_), 5.42 (d, 1H, *J* = 15.8 Hz, N-1–CH*α*), 5.52 (d, 1H, *J* = 15.8 Hz, N-1–CH*β*), 7.27–7.30 (m, 1H, H-6), 7.41–7.47 (m, 1H, H-4^E^), 7.49–7.55 (m, 2H, H-3^E^, H-5^E^), 7.69–7.74 (m, 2H, H-2^E^, H-6^E^), 7.76 (ddd, 1H, *J* = 8.1, 7.7, 1.6 Hz, H-7), 7.85 (d, 1H, *J* = 7.3 Hz, H-8), 7.86 (s, 1H, H-5^A^), 8.05 (dd, 1H, *J* = 7.8, 1.5 Hz, H-5), 8.10 (s, 1H, H-5^D^); ^13^C NMR (126 MHz, CDCl_3_) *δ* 21.0 (CO*C*H_3_), 23.4 (C-3–*C*H_3_), 39.5 (N-1-CH_2_), 57.7 (OCH_2_), 71.5 (C-3), 116.8 (C-8), 119.2 (C-4a), 120.6 (C-2^E^, C-6^E^), 121.7 (C-5^D^), 124.1 (C-5^A^), 124.7 (C-6), 129.1 (C-4^E^), 129.4 (C-5), 129.9 (C-3^E^, C-5^E^), 136.9 (C-1^E^), 137.8 (C-7), 141.7 (C-8a), 142.3 (C-4^A^), 143.2 (C-4^D^), 168.3 (C-2), 171.1 (*C*OCH_3_), 189.4 (C-4); ^15^N NMR (51 MHz, CDCl_3_) *δ* 138.7 (N-1), 248.8 (N-1^A^), 256.3 (N-1^D^), 351.9 (N-3^D^), 355.5 (N-3^A^); IR (cm^−1^): *ν* 3145, 2926, 1740, 1717, 1681, 1601, 1470, 1384, 1242, 1184, 1046, 761, 691, 664; MS (EI) *m*/*z* (%): 472 (0.9, [M + 1]^+^), 471 (3, [M]^+^), 303 (20), 302 (17), 131 (13), 130 (100), 129 (14), 77 (44), 43 (25); HRMS (ESI+): *m*/*z* calcd for C_24_H_22_N_7_O_4_^+^ [M + H]^+^ 472.1728, found 472.1726.

**1-((1-Benzyl-1*H*-1,2,3-triazol-4-yl)methyl)-3-phenyl-3-(4-phenyl-1*H*-1,2,3-triazol-1-yl)quinoline-2,4(1*H*,3H)-dione (2g).** Colorless crystals, m.p. 142–145 °C (ethanol); *R*_f_ = 0.42 (30% ethyl acetate in chloroform); ^1^H NMR (500 MHz, DMSO-*d*_6_) *δ* 5.15 (d, 1H, *J* = 15.8 Hz, N-1–CH*α*), 5.62 (s, 2H, N-1^D^–CH_2_), 5.63 (d, 1H, *J* = 15.8 Hz, N-1–CH*β*), 7.22–7.50 (m, 14H, H-6, H-3^B^, H-4^B^, H-5^B^, H-2^C^, H-3^C^, H-4^C^, H-5^C^, H-6^C^, H-2^E^, H-3^E^, H-4^E^, H-5^E^, H-6^E^), 7.68 (d, 1H, *J* = 7.8 Hz, H-8), 7.73 (ddd, 1H, *J* = 8.5, 7.1, 1.7 Hz, H-7), 7.80–7.83 (m, 2H, H-2^B^, H-6^B^), 7.92 (dd, 1H, *J* = 7.7, 1.5 Hz, H-5), 8.24 (s, 1H, H-5^D^), 8.51 (s, 1H, H-5^A^); ^13^C NMR (126 MHz, DMSO-*d*_6_) *δ* 39.2 (N-1–CH_2_), 52.8 (N-1^D^–CH_2_), 80.1 (C-3), 116.7 (C-8), 120.9 (C-4a), 123.4 (C-5^A^), 124.0 (C-6), 124.2 (C-5^D^), 125.2 (C-2^B^, C-6^B^), 127.9 (C-5), 128.0 (C-4^C^, C-2^E^, C-6^E^), 128.2 (C-4^B^),128.7 (C-1^C^), 128.8 (C-3^E^, C-5^E^), 129.0 (C-3^B^, C-5^B^), 129.4 (C-2^C^, C-6^C^), 129.8 (C-1^B^), 130.5 (C-3^C^, C-5^C^, C-4^E^), 136.0 (C-1^E^), 136.8 (C-7), 140.8 (C-8a), 141.9 (C-4^D^), 145.4 (C-4^A^), 166.2 (C-2), 188.2 (C-4); IR (cm^−1^): *ν* 3434, 3138, 3062, 1716, 1678, 1601, 1468, 1375, 1307, 1035, 870, 761, 724, 695; MS (EI) *m*/*z* (%): 552 (1, [M + 1]^+^), 551 (3, [M]^+^), 289 (23), 236 (11), 145 (18), 144 (17), 116 (31), 104 (10), 91 (100), 89 (11), 77 (16); HRMS (ESI+): *m*/*z* calcd for C_33_H_26_N_7_O_2_^+^ [M + H]^+^ 552.2142, found 552.2133. Anal. Calcd for C_33_H_25_N_7_O_2_ (551.60): C, 71.86; H, 4.57; N, 17.78%. Found: C, 71.58; H, 4.58; N, 17.73%.

**3-Phenyl-3-(4-phenyl-1*H*-1,2,3-triazol-1-yl)-1-((1-phenyl-1*H*-1,2,3-triazol-4-yl)methyl)quinoline-2,4(1*H*,3*H*)-dione (2h).** Colorless crystals, m.p. 152–157 °C (ethanol); *R*_f_ = 0.54 (30% ethyl acetate in chloroform); ^1^H NMR (500 MHz, DMSO-*d*_6_) *δ* 5.33 (d, 1H, *J* = 16.1 Hz, N-1–CH*α*), 5.71 (d, 1H, *J* = 16.1 Hz, N-1–CH*β*), 7.25–7.29 (m, 1H, H-6), 7.32–7.48 (m, 8H, H-3^B^, H-4^B^, H-5^B^, H-2^C^, H-3^C^, H-4^C^, H-5^C^, H-6^C^), 7.48–7.53 (m, 1H, H-4^E^), 7.58–7.64 (m, 2H, H-3^E^, H-5^E^), 7.70 (d, 1H, *J* = 8.2 Hz, H-8), 7.72–7.77 (m, 1H, H-7), 7.81–7.85 (m, 2H, H-2^B^, H-6^B^), 7.88–7.93 (m, 2H, H-2^E^, H-6^E^), 7.95 (dd, 1H, *J* = 7.7, 1.5 Hz, H-5), 8.54 (s, 1H, H-5^A^), 8.83 (s, 1H, H-5^D^); ^13^C NMR (126 MHz, DMSO-*d*_6_) *δ* 39.0 (N-1–CH_2_), 80.3 (C-3), 116.7 (C-8), 120.2 (C-2^E^, C-6^E^), 120.9 (C-4a), 122.3 (C-5^D^), 123.5 (C-5^A^), 124.1 (C-6), 125.2 (C-2^B^, C-6^B^), 127.9 (C-5), 128.0 (C-4^B^), 128.9 (C-4^E^), 128.9 (C-2^C^, C-6^C^), 129.0 (C-3^B^, C-3^B^), 129.4 (C-3^C^, C-5^C^), 129.9 (C-1^C^), 130.0 (C-3^E^, C-5^E^), 130.5 (C-4^C^), 130.6 (C-1^B^), 136.5 (C-1^E^), 136.8 (C-7), 140.7 (C-8a), 142.9 (C-4^D^), 145.4 (C-4^A^), 166.4 (C-2), 188.2 (C-4); IR (cm^−1^): *ν* 3447, 3142, 3060, 1716, 1679, 1600, 1468, 1449, 1375, 1305, 1040, 871, 758, 693; MS (EI) *m*/*z* (%): 538 (1, [M + 1]^+^), 537 (3, [M]^+^), 366 (14), 262 (10), 236 (17), 145 (29), 131 (10), 130 (100), 129 (19), 128 (11), 118 (10), 116 (38), 104 (14), 103 (17), 102 (13), 90 (12), 89 (15), 77 (71), 51 (12); HRMS (ESI+): *m*/*z* calcd for C_32_H_24_N_7_O_2_^+^ ([M+H]^+^) 538.1986, found 538.1976. Anal. Calcd for Anal. calcd for C_32_H_23_N_7_O_2_ (537.57) C, 71.50; H, 4.31; N, 18.24%. Found: C, 71.22; H, 4.32; N, 17.94%.

**(1-(1-((1-Benzyl-1*H*-1,2,3-triazol-4-yl)methyl)-2,4-dioxo-3-phenyl-1,2,3,4-tetrahydroquinolin-3-yl)-1*H*-1,2,3-triazol-4-yl)methyl acetate (2j).** Colorless powder, m.p. 188–194 °C (ethanol); *R*_f_ = 0.41 (30% ethyl acetate in chloroform); ^1^H NMR (500 MHz, CDCl_3_) *δ* 2.04 (s, 3H, CH_3_), 5.17 (s, 2H, OCH_2_), 5.21 (d, 1H, *J* = 15.6 Hz, N-1–CH*α*), 5.43 (d, 1H, *J* = 14.8 Hz, N-1^D^–CH*α*), 5.51 (d, 1H, *J* = 15.6 Hz, N-1–CH*β*), 5.55 (d, 1H, *J* = 14.8 Hz, N-1^D^–CH*β*), 7.08 (s, 1H, H-5^A^), 7.18 (ddd, 1H, *J* = 7.5, 7.5, 0.8 Hz, H-6), 7.23–7.29 (m, 4H, H-3^C^, H-5^C^, H-2^E^, H-6^E^), 7.29–7.33 (m, 2H, H-2^C^, H-6^C^), 7.34–7.39 (m, 3H, H-3^E^, H-4^E^, H-5^E^), 7.38–7.44 (m, 1H, H-4^C^), 7.58 (s, 1H, H-5^D^), 7.63 (ddd, 1H, *J* = 8.4, 7.4, 1.7 Hz, H-7), 7.75 (d, 1H, *J* = 8.3 Hz, H-8), 7.99 (dd, 1H, *J* = 7.7, 1.7 Hz, H-5); ^13^C NMR (126 MHz, CDCl_3_) *δ* 21.0 (CH_3_), 39.9 (N-1–CH_2_), 54.5 (N-1^D^–CH_2_), 57.6 (OCH_2_), 79.6 (C-3), 116.8 (C-8), 120.9 (C-4a), 123.5 (C-5^D^), 124.6 (C-6), 126.4 (C-5^A^), 128.3 (C-2^E^, C-6^E^), 128.7 (C-2^C^, C-6^C^), 129.0 (C-5), 129.1 (C-4^E^), 129.4 (C-3^E^, C-5^E^), 129.7 (C-1^C^), 130.0 (C-3^C^, C-5^C^), 131.3 (C-4^C^), 134.5 (C-1^E^), 137.2 (C-7), 140.9 (C-4^A^), 141.1 (C-8a), 142.9 (C-4^D^), 166.6 (C-2), 171.0 (*C*OCH_3_), 187.9 (C-4); ^15^N NMR (51 MHz, CDCl_3_) *δ* 140.4 (N-1), 249.8 (N-1^A^), 250.4 (N-1^D^), 350.5 (N-3^D^), 356.9 (N-3^A^), 362.9 (N-2^D^), 365.1 (N-2^A^); IR (cm^−1^): *ν* 3142, 2927, 1740, 1717, 1679, 1602, 1469, 1377, 1244, 768, 749, 714, 697; MS (EI) *m*/*z* (%): 548 (0.1, [M + 1]^+^), 547 (0.3, [M]^+^), 347 (13), 289 (13), 144 (14), 105 (10), 104 (13), 91 (100), 43 (29); HRMS (ESI+): *m*/*z* calcd for C_30_H_26_N_7_O_4_^+^ [M + H]^+^ 548.2041, found 548.2032.

**(1-(2,4-Dioxo-3-phenyl-1-((1-phenyl-1*H*-1,2,3-triazol-4-yl)methyl)-1,2,3,4-tetrahydroquinolin-3-yl)-1*H*-1,2,3-triazol-4-yl)methyl acetate (2k).** Colorless powder, m.p. 93–105 °C; *R*_f_ = 0.42 (30% ethyl acetate in chloroform); ^1^H NMR (500 MHz, CDCl_3_) *δ* 2.05 (s, 3H, CH_3_), 5.19 (s, 2H, OCH_2_), 5.42 (d, 1H, *J* = 15.7 Hz, N-1–CH*α*), 5.55 (d, 1H, *J* = 15.7 Hz, N-1–CH*β*), 7.14 (s, 1H, H-5^A^), 7.20 (ddd, 1H, *J* = 7.6, 7.6, 0.8 Hz, H-6), 7.38–7.49 (m, 6H, H-2^C^, H-3^C^, H-4^C^, H-5^C^, H-6^C^, H-4^E^), 7.49–7.55 (m, 2H, H-3^E^, H-5^E^), 7.66 (ddd, 1H, *J* = 8.5, 7.3, 1.7 Hz, H-7), 7.68–7.72 (m, 2H, H-2^E^, H-6^E^), 7.76 (d, 1H, *J* = 8.4 Hz, H-8), 8.03 (dd, 1H, *J* = 7.8, 1.5 Hz, H-5), 8.05 (s, 1H, H-5^D^); ^13^C NMR (126 MHz, CDCl_3_) *δ* 21.0 (CH_3_), 39.8 (N-1-CH_2)_, 57.6 (OCH_2_), 79.6 (C-3), 116.7 (C-8), 120.7 (C-2^E^, C-6^E^), 120.9 (C-4a), 121.8 (C-5^D^), 124.7 (C-6), 126.4 (C-5^A^), 128.9 (C-2^C^, C-6^C^), 129.1 (C-5), 129.2 (C-4^E^), 129.9 (C-1^C^), 130.0 (C-3^E^, C-5^E^), 130.2 (C-3^C^, C-5^C^), 131.4 (C-4^C^), 136.9 (C-1^E^), 137.4 (C-7), 140.9 (C-4^A^), 140.9 (C-8a), 143.2 (C-4^D^), 166.9 (C-2), 171.0 (*C*OCH_3_), 187.9 (C-4); ^15^N NMR (51 MHz, CDCl_3_) *δ* 140.4 (N-1), 249.9 (N-1^A^), 256.3 (N-1^D^), 352.9 (N-3^D^), 357.2 (N-3^A^); IR (cm^−1^): *ν* 3146, 2962, 1741, 1718, 1681, 1600, 1468, 1376, 1243, 1043, 762, 693, 665, 608; MS (EI) *m*/*z* (%): 534 (0.2, [M + 1]^+^), 533 (0.6, [M]^+^), 366 (12), 365 (11), 262 (12), 131 (11), 130 (100), 129 (19), 128 (12), 104 (14), 103 (16), 99 (18), 77 (62), 44 (17), 43 (52); HRMS (ESI+): *m*/*z* calcd for C_29_H_24_N_7_O_4_^+^ [M + H]^+^ 534.1884, found 534.1882.

### 3.9. General Procedure for the Synthesis of Bis-Triazoles 2c,f,i,l by Employing CuSO_4_/Cu^0^/DMF Conditions (Table 4, Entries 3, 9, 12 and 15)

A mixture of the appropriate *N*-propargylquinoline-2,4(1*H*,3*H*)-dione **7** (1.5 mmol), tetrazolo[1,5-*a*]pyridine (189 mg, 1.58 mmol), CuSO_4_∙5H_2_O (38 mg, 0.15 mmol), granular copper (191 mg, 3.05 mmol) and DMF (9 mL) was heated in darkness to 95–105 °C (oil bath) for the time given in Table 4, whereas the color of the mixture changed from brown-black to dark green. The mixture was then allowed to cool to room temperature. Subsequently, (NH_4_)_2_CO_3_ (432 mg, 4.5 mmol) and water (2 mL) were added and after stirring for 15 min, the mixture was poured into a narrow (1 cm diameter) column of silica gel (15 g). The organic portion was eluted from the column with 10% ethanol in chloroform. The yellow eluate was washed with saturated aqueous NH_4_Cl (50 mL), dried over anhydrous sodium sulfate, filtered, and the solvent was removed by rotary evaporation *in vacuo*. In the cases of **2c**,**i**, the residue, which was TLC pure compound, was crystallized from suitable solvent. In the cases of **2f**,**l**, the residue was purified by chromatography on silica gel column using chloroform as eluent. The yields of prepared compounds **2** are given in Table 4.

**3-Methyl-3-(4-phenyl-1*H*-1,2,3-triazol-1-yl)-1-((1-(pyridin-2-yl)-1*H*-1,2,3-triazol-4-yl)methyl)quinoline-2,4(1*H*,3*H*)-dione (2c).** Colorless crystals, m.p. 188–191 °C (benzene); *R*_f_ = 0.29 (30% ethyl acetate in chloroform); ^1^H NMR (500 MHz, DMSO-*d*_6_) *δ* 2.23 (s, 3H, CH_3_), 5.42 (d, 1H, *J* = 16.5 Hz, N-1–CH*α*), 5.58 (d, 1H, *J* = 16.5 Hz, N-1–CH*β*), 7.28–7.40 (m, 2H, H-6, H-4^B^), 7.43–7.51 (m, 2H, H-3^B^, H-5^B^), 7.51–7.57 (m, 1H, H-5^E^), 7.64 (d, 1H, *J* = 8.4 Hz, H-8), 7.78–7.90 (m, 3H, H-7, H-2^B^, H-6^B^), 7.99 (d, 1H, *J* = 7.5 Hz, H-5), 8.07–8.17 (m, 2H, H-3^E^, H-4^E^), 8.54–8.61 (m, 1H, H-6^E^), 8.82 (s, 1H, H-5^D^), 8.87 (s, 1H, H-5^A^); ^13^C NMR (126 MHz, DMSO-*d*_6_) *δ* 23.4 (CH_3_), 38.7 (N-1–CH_2_), 73.0 (C-3), 113.7 (C-3^E^), 116.6 (C-8), 119.3 (C-4a), 120.6 (C-5^D^), 122.5 (C-5^A^), 123.9 (C-6), 124.5 (C-5^E^), 125.2 (C-2^B^, C-6^B^), 128.1 (C-4^B^), 128.1 (C-5), 129.1 (C-3^B^, C-5^B^), 130.5 (C-1^B^), 137.2 (C-7), 140.3 (C-4^E^), 141.4 (C-8a), 143.2 (C-4^D^), 146.0 (C-4^A^), 148.4 (C-2^E^), 149.0 (C-6^E^), 168.5 (C-2), 189.9 (C-4); ^15^N NMR (51 MHz, DMSO-*d*_6_) *δ* 135.8 (N-1), 248.9 (N-1^A^), 260.5 (N-1^D^), 284.9 (N-1^E^), 347.1 (N-3^A^), 356.9 (N-3^D^), 358.6 (N-2^D^), 363.4 (N-2^A^); IR (cm^−1^): *ν* 3426, 3126, 2972, 1706, 1674, 1601, 1471, 1378, 1310, 1232, 1041, 777, 764; MS (EI) *m*/*z* (%): 477 (2, [M + 1]^+^), 476 (7, [M]^+^), 289 (11), 145 (14), 132 (14), 131 (96), 116 (50), 102 (10), 90 (10), 89 (13), 79 (20), 78 (100), 77 (10), 51 (10); HRMS (ESI+): *m*/*z* calcd for C_26_H_21_N_8_O_2_^+^ [M + H]^+^ 477.1782, found 477.1773. Anal. Calcd for C_26_H_20_N_8_O_2_ (476.48) C, 65.54; H, 4.23; N, 23.52%. Found: C, 65.68; H, 4.21; N, 23.63%.

**(1-(3-Methyl-2,4-dioxo-1-((1-(pyridin-2-yl)-1*H*-1,2,3-triazol-4-yl)methyl)-1,2,3,4-tetrahydroquinolin-3-yl)-1*H*-1,2,3-triazol-4-yl)methyl acetate (2f).** Colorless powder, m.p. 69–82 °C; *R*_f_ = 0.29 (30% ethyl acetate in chloroform); ^1^H NMR (500 MHz, DMSO-*d*_6_) *δ* 2.06 (s, 3H, COCH_3_), 2.18 (s, 3H, C3–CH_3_), 5.17 (d, 1H, *J* = 12.7 Hz, O–CH*α*), 5.20 (d, 1H, *J* = 12.7 Hz, O–CH*β*), 5.41 (d, 1H, *J* = 16.5 Hz, N-1–CH*α*), 5.53 (d, 1H, *J* = 16.5 Hz, N-1–CH*β*), 7.31 (dd, 1H, *J* = 7.4, 7.4 Hz, H-6), 7.54 (dd, 1H, *J* = 8.8, 4.5 Hz, H-5^E^), 7.59 (d, 1H, *J* = 8.5 Hz, H-8), 7.77–7.83 (m, 1H, H-7), 7.96 (dd, 1H, *J* = 7.7 Hz, *J* = 1.6 Hz, H-5), 8.08–8.14 (m, 2H, H-3^E^, H-4^E^), 8.47 (s, 1H, H-5^A^), 8.55–8.59 (m, 1H, H-6^E^), 8.82 (s, 1H, H-5^D^); ^13^C NMR (126 MHz, DMSO-*d*_6_) *δ* 20.6 (CO*C*H_3_), 23.5 (C3–*C*H_3_), 38.7 (N-1–CH_2_), 57.2 (OCH_2_), 73.3 (C-3), 113.7 (C-3^E^), 116.5 (C-8), 119.4 (C-4a), 120.6 (C-5^D^), 123.8 (C-6), 124.4 (C-5^E^), 126.1 (C-5^A^), 127.9 (C-5), 137.0 (C-7), 140.2 (C-4^E^), 141.3 (C-8a), 141.6 (C-4^A^), 143.2 (C-4^D^), 148.3 (C-2^E^), 148.9 (C-6^E^), 168.6 (C-2), 170.1 (*C*OCH_3_), 189.9 (C-4); ^15^N NMR (51 MHz, DMSO-*d*_6_) *δ* 135.3 (N1), 247.6 (N-1^A^), 260.0 (N-1^D^), 284.7 (N-1^E^), 353.4 (N-3^A^), 356.5 (N-3^D^), 361.9 (N-2^D^), 363.7 (N-2^A^); IR (cm^−1^): *ν* 3152, 1741, 1718 1681, 1600, 1471, 1384, 1314, 1242, 1183, 1038, 782, 756, 663; MS (EI) *m*/*z* (%): 473 (0.7, [M + 1]^+^), 472 (2, [M]^+^), 304 (27), 303 (26), 302 (17), 132 (13), 131 (100), 79 (22), 78 (100), 43 (21); HRMS (ESI+): *m*/*z* calcd for C_23_H_21_N_8_O_4_^+^ [M + H]^+^ 473.1680, found 473.1684. Anal. Calcd for C_23_H_20_N_8_O_4_·½H_2_O (472.46): C, 57.38; H, 4.40; N, 23.27%. Found: C, 57.39; H, 4.36; N, 23.47%.

**3-Phenyl-3-(4-phenyl-1*H*-1,2,3-triazol-1-yl)-1-((1-(pyridin-2-yl)-1*H*-1,2,3-triazol-4-yl)methyl)quinoline-2,4(1*H*,3*H*)-dione (2i).** Colorless crystals, m.p. 188–192 °C (benzene); *R*_f_ = 0.50 (30% ethyl acetate in chloroform); ^1^H NMR (500 MHz, DMSO-*d*_6_) *δ* 5.44 (d, 1H, *J* = 16.3 Hz, N-1–CH*α*), 5.67 (d, 1H, *J* = 16.3 Hz, N-1–CH*β*), 7.26 (dd, 1H, *J* = 7.5, 7.5 Hz, H-6), 7.32–7.40 (m, 3H, H-4^B^, H-2^C^, H-6^C^), 7.41–7.52 (m, 5H, H-3^B^, H-5^B^, H-3^C^, H-4^C^, H-5^C^), 7.52–7.61 (m, 2H, H-8, H-5^E^), 7.68–7.75 (m, 1H, H-7), 7.78–7.86 (m, 2H, H-2^B^, H-6^B^), 7.96 (dd, 1H, *J* = 7.7, 1.5 Hz, H-5), 8.09–8.16 (m, 2H, H-3^E^, H-4^E^), 8.58 (s, 1H, H-5^A^), 8.60 (ddd, 1H, *J* = 4.8, 1.3, 1.3 Hz, H-6^E^), 8.81 (s, 1H, H-5^D^); ^13^C NMR (126 MHz, DMSO-*d*_6_) *δ* 39.1 (N-1–*C*H_2_), 80.4 (C-3), 113.7 (C-3^E^), 116.5 (C-8), 120.8 (C-5^D^), 120.9 (C-4a), 123.4 (C-5^A^), 124.0 (C-6), 124.5 (C-5^E^), 125.2 (C-2^B^, C-6^B^), 127.9 (C-5), 128.0 (C-4^B^), 128.9 (C-2^C^, C-6^C^), 129.0 (C-3^B^, C-5^B^), 129.4 (C-3^C^, C-5^C^), 130.0 (C-1^C^), 130.5 (C-4^C^), 130.6 (C-1^B^), 136.8 (C-7), 140.2 (C-4^E^), 140.5 (C-8a), 143.0 (C-4^D^), 145.4 (C-4^A^), 148.3 (C-2^E^), 149.0 (C-6^E^), 166.6 (C-2), 188.1 (C-4); ^15^N NMR (51 MHz, DMSO-*d*_6_) *δ* 137.5 (N1), 248.7 (N-1^A^), 260.4 (N-1^D^), 284.8 (N-1^E^), 347.2 (N-3^A^), 357.7 (N-3^D^), 367.4 (N-2^A^); IR (cm^−1^): *ν* 3418, 2973, 1718, 1679, 1596, 1477, 1467, 1450, 1049, 1031, 773, 766, 757, 701; MS (EI) *m*/*z* (%): 539 (1, [M + 1]^+^), 538 (3, [M]^+^), 236 (11), 145 (16), 132 (14), 131 (100), 116 (32), 91 (11), 89 (11), 79 (15), 78 (85), 77 (13); HRMS (ESI+): *m*/*z* calcd for C_31_H_23_N_8_O_2_^+^ [M + H]^+^ 539.1938, found 539.1932. Anal. calcd for C_31_H_22_N_8_O_2_ (538.19): C, 69.13; H, 4.12; N, 20.81%. Found: C, 68.91; H, 4.17; N, 20.66%.

**(1-(2,4-Dioxo-3-phenyl-1-((1-(pyridin-2-yl)-1*H*-1,2,3-triazol-4-yl)methyl)-1,2,3,4-tetrahydroquinolin-3-yl)-1*H*-1,2,3-triazol-4-yl)methyl acetate (2l).** Colorless powder, m.p. 93–102 °C; *R*_f_ = 0.18 (30% ethyl acetate in chloroform); ^1^H NMR (500 MHz, CDCl_3_) *δ* 2.05 (s, 3H, COCH_3_), 5.19 (s, 2H, OCH_2_), 5.30 (d, 1H, *J* = 15.8 Hz, N-1–CH*α*), 5.71 (d, 1H, *J* = 15.8 Hz, N-1–CH*β*), 7.13 (s, 1H, H-5^A^), 7.19 (dd, 1H, *J* = 7.5, 7.5 Hz, H-6), 7.36 (dd, 1H, *J* = 7.3, 4.9 Hz, H-5^E^), 7.38–7.42 (m, 2H, H-3^C^, H-5^C^), 7.42–7.48 (m, 3H, H-2^C^, H-4^C^, H-6^C^), 7.63 (ddd, 1H, *J* = 8.3, 7.4, 1.6 Hz, H-7), 7.70 (d, 1H, *J* = 8.4 Hz, H-8), 7.88–7.95 (m, 1H, H-4^E^), 8.02 (dd, 1H, *J* = 7.8, 1.5 Hz, H-5), 8.15 (d, 1H, *J* = 8.2 Hz, H-3^E^), 8.47–8.53 (m, 1H, H-6^E^), 8.63 (s, 1H, H-5^D^); ^13^C NMR (126 MHz, CDCl_3_) *δ* 21.0 (CO*C*H_3_), 39.9 (N-1–CH_2_), 57.6 (OCH_2_), 79.7 (C-3), 113.9 (C-3^E^), 116.6 (C-8), 121.0 (C-4a), 121.0 (C-5^D^), 124.0 (C-5^E^), 124.6 (C-6), 126.4 (C-5^A^), 128.9 (C-2^C^, C-6^C^), 129.1 (C-5), 129.7 (C-1^C^), 130.2 (C-3^C^, C-5^C^), 131.3 (C-4^C^), 137.2 (C-7), 139.3 (C-4^E^), 140.9 (C-4^A^), 141.2 (C-8a), 143.0 (C-4^D^), 148.9 (C-6^E^), 149.0 (C-2^E^), 166.6 (C-2), 171.0 (*C*OCH_3_), 187.9 (C-4); ^15^N NMR (51 MHz, CDCl_3_) *δ* 138.9 (N-1), 249.7 (N-1^A^), 261.2 (N-1^D^), 285.1 (N-1^E^), 355.8 (N-3^D^), 357.1 (N-3^A^); IR (cm^−1^): *ν* 3155, 2926, 1741, 1718, 1682, 1599, 1470, 1375, 1313, 1243, 1034, 779, 697, 665; MS (EI) *m*/*z* (%): 535 (0.4, [M + 1]^+^), 534 (0.7, [M]^+^), 132 (14), 131 (100), 79 (19), 78 (93), 44 (11), 43 (31); HRMS (ESI+): *m*/*z* calcd for C_28_H_23_N_8_O_4_^+^ [M + H]^+^ 535.1837, found 535.1846.

### 3.10. Synthesis of Bis-Triazole 2d by Employing CH_2_Cl_2_/Water/CuSO_4_∙5H_2_O/Na-Ascorbate Conditions (Table 4, Entry 5)

To a solution of acetylene **7c** (132 mg, 0.375 mmol) and azide **8a** (52.4 mg, 0.394 mmol) in dichloromethane (6.5 mL) a solution of sodium ascorbate (59.5 mg, 0.3 mmol) in water (5.5 mL), and a solution of CuSO_4_∙5H_2_O (7.5 mg, 0.03 mmol) in water (1 mL) were added. The two-phase liquid reaction mixture was stirred in darkness at room temperature until the compound **7c** reacted completely according to TLC analysis (4 h). The reaction mixture was diluted with water (50 mL) and extracted with chloroform (4 × 30 mL). The combined organic layers were dried (Na_2_SO_4_), filtered, and evaporated to dryness. The residue was dissolved in chloroform (5 mL) and subjected to silica gel (25 g) column chromatography using 67% ethyl acetate in petroleum ether as eluent, affording product **2d** (155 mg, 0.32 mmol, 85%).

### 3.11. Synthesis of Bis-Triazole 2d by Employing t-BuOH/Water/CH_3_CN/CuSO_4_∙5H_2_O/Na-Ascorbate Conditions (Table 4, Entry 6)

To a mixture of acetylene **7c** (264 mg, 0.75 mmol), azide **8a** (105 mg, 0.79 mmol) and *t*-BuOH (3.5 mL) a solution of Na-ascorbate (30 mg, 0.15 mmol) in water (2.5 mL), and a solution of CuSO_4_∙5H_2_O (4 mg, 0.02 mmol) in water (1 mL) were added. The reaction mixture was stirred in darkness at room temperature for 9 h. Then a solution of Na-ascorbate (89 mg, 0.45 mmol) in water (1 mL), and a solution of CuSO_4_∙5H_2_O (11 mg, 0.044 mmol) in water (1 mL) and *t*-BuOH (2 mL) were added. The reaction mixture was stirred for additional 20 h. The resulting sticky sediment that formed in the course of the reaction was dissolved by addition of acetonitrile (3 mL) to the reaction mixture. The reaction mixture was stirred for additional 19 h. Although the azide and acetylene coupling partners were still present in the reaction mixture, as judged by TLC analysis, the reaction was stopped by the addition of water (50 mL) and extracted with chloroform (4 × 30 mL). The combined organic layers were dried (Na_2_SO_4_), filtered, and evaporated to dryness. The residue was dissolved in chloroform and subjected to silica gel (35 g) column chromatography using 67% ethyl acetate in petroleum ether as eluent, affording regenerated starting acetylene **7c** (48 mg, 0.14 mmol, 18%) and product **2d** (295 mg, 0.61 mmol, 81%).

### 3.12. Synthesis of Bis-Triazole 2d by Employing t-BuOH/Water/CuSO_4_∙5H_2_O/l-Ascorbic Acid Conditions (Table 4, Entry 7)

To a mixture of acetylene **7c** (264 mg, 0.75 mmol) and azide **8a** (105 mg, 0.79 mmol) a solution of l-ascorbic acid (13 mg, 0.074 mmol) and CuSO_4_∙5H_2_O (2 mg, 0.008 mmol) in water (3.5 mL), and *t*-BuOH (3.5 mL) were added. The reaction mixture was stirred in darkness at room temperature. After 8.5 h and 22 h of stirring additional portions of l-ascorbic acid/CuSO_4_∙5H_2_O/water/*t*-BuOH (40 mg, 0.23 mmol/6 mg, 0.02 mmol/1 mL/1 mL and 53 mg, 0.3 mmol/7.5 mg, 0.03 mmol/1 mL/1 mL, respectively) were added. Although after stirring for additional 23 h (total reaction time 45 h), TLC analysis indicated the presence of azide and acetylene starting compounds, the heterogeneous reaction mixture (a sticky sediment was formed) was diluted with water and extracted with chloroform (5 × 50 mL). The combined organic layers were dried (Na_2_SO_4_), filtered, and evaporated to dryness. The residue was dissolved in chloroform (5 mL) and subjected to silica gel (35 g) column chromatography using 33% ethyl acetate in petroleum ether as eluent, affording regenerated starting acetylene **7c** (114 mg, 0.32 mmol, 43%) and product **2d** (165 mg, 0.34 mmol 45%).

### 3.13. 3-Azido-3-methyl-1-(prop-2-yn-1-yl)Quinoline-2,4(1H,3H)-Dione (9a) (Scheme 3)

A mixture of the azide **5a** (649 mg, 3.0 mmol) and potassium carbonate (1.24 g, 9 mmol) in DMF (15 mL) was stirred at room temperature in darkness for 40 min. Propargyl bromide (**6c**, 80% solution in toluene, 669 mg, 4.5 mmol) diluted with DMF (7 mL) was added dropwise under stirring during 1 min. The reaction mixture was stirred for 6 h, during which time it turned yellow, diluted with cold water (200 mL) and extracted with chloroform (5 × 50 mL). The combined organic layers were dried (Na_2_SO_4_), filtered, and evaporated to dryness. Trace amounts of DMF were removed by five subsequent co-destilations *in vacuo* at 50 °C with toluene (30 mL). The residual yellow oil was dissolved in chloroform (5 mL) and chromatographed on column silica gel (35 g) using chloroform as eluent, affording product **9a** (717 mg, 2.82 mmol, 94%, dried *in vacuo* to constant weight) as off-white oily material, that was pure by TLC (*R*_f_ = 0.57; chloroform); ^1^H NMR (500 MHz, CDCl_3_) *δ* 1.79 (s, 3H), 2.29 (dd, 1H, *J* = 2.5, 2.5 Hz), 4.67 (dd, 1H, *J* = 17.8, 2.5 Hz), 4.98 (dd, 1H, *J* = 17.8, 2.5 Hz), 7.26 (ddd, 1H, *J* = 7.7, 7.4, 0.8 Hz), 7.35 (d, 1H, *J* = 8.3 Hz), 7.71 (ddd, 1H, *J* = 8.3, 7.4, 1.7 Hz), 8.02 (dd, 1H, *J* = 7.7, 1.7 Hz); ^13^C NMR (126 MHz, CDCl_3_) *δ* 23.6, 32.7, 70.7, 73.4, 77.1, 115.8, 119.6, 124.4, 129.0, 136.9, 140.8, 169.1, 191.1; IR (cm^−1^): *ν* 3241, 2980, 2138, 2107, 1711, 1678, 1603, 1471, 1383, 1366, 1305, 1285, 1260, 1218, 762; HRMS (ESI+): *m*/*z* calcd for C_13_H_11_N_4_O_2_^+^ [M + H]^+^ 255.0877, found 255.0877; calcd for C_13_H_11_N_2_O_2_^+^ [M − N_2_ + H]^+^ 227.0815, found 227.0814.

### 3.14. Synthesis of Triazole ***7a*** from Phenylacetylene (***6a***) and Compound ***9a*** (Scheme 3)

A mixture of compound **9a** (286 mg, 1.13 mmol), phenylacetylene (**6a**) (230 mg, 2.25 mmol), CuSO_4_ 5H_2_O (28 mg, 0.11 mmol) and granular copper (143 mg, 2.25 mmol) in DMF (5 mL) was stirred in darkness at room temperature for 60 min. To the resulting brown-green suspension (NH_4_)_2_CO_3_ (324 mg, 3.38 mmol) and water (3 mL) were added and stirring was continued for 10 min. The resulting mixture was diluted with 10% ethanol in chloroform (10 mL). The organic layer was separated and the aqueous layer was extracted with chloroform (3 × 10 mL). The combined organic layers were passed through a narrow (1 cm in diameter) column of silica gel (13 g) and the column was subsequently washed with 10% ethanol in chloroform (210 mL) using overpressure to the top of the column. The yellow eluate was washed with saturated aqueous NH_4_Cl (1 × 50 mL) and distilled water (1 × 50 mL), dried (Na_2_SO_4_), filtered, and evaporated to dryness. Trace amounts of DMF were removed by five subsequent co-destilations *in vacuo* at 50 °C with toluene (40 mL). The residue was chromatographed on a column of silica gel (30 g) using 38% ethyl acetate in hexane. The resulting white solid (88 mg) was crystallized from benzene affording triazole **7a** (66 mg, 0.19 mmol, 16%), which was identified with the compound **7a** described above.

### 3.15. 3-Azido-1-((1-Benzyl-1H-1,2,3-Triazol-4-yl)Methyl)-3-Methylquinoline-2,4(1H,3H)-Dione (***10a***) (Scheme 3)

A mixture of acetylene **9a** (254 mg, 1.0 mmol), (azidomethyl)benzene (**8a**) (266 mg, 2.0 mmol), CuSO_4_∙5H_2_O (25 mg, 0.1 mmol) and granular copper (127 mg, 2.0 mmol) in DMF (10 mL) was stirred at room temperature for 21 h. Then (NH_4_)_2_CO_3_ (288 mg, 3.0 mmol) and water (3 mL) were added and the stirring was continued for 10 min. The resulting mixture was poured into a narrow (1 cm in diameter) column of silica gel (13 g). The organic portion was eluted with 10% ethanol in chloroform (190 mL). The yellow eluate was washed with saturated aqueous NH_4_Cl (50 mL) and water (50 mL), dried (Na_2_SO_4_), filtered, and evaporated to dryness. Trace amounts of DMF were removed by six subsequent co-destilations *in vacuo* at 50 °C with toluene (30 mL). The residue was dissolved in chloroform (5 mL) and chromatographed on silica gel (35 g) column using gradually 38% and 50% ethyl acetate in petroleum ether as mobile phase, affording product **10a** (164 mg, 0.42 mmol, 42%) as a white solid, m.p. 42–47 °C; *R*_f_ = 0.21 (38% ethyl acetate in petroleum ether); ^1^H NMR (500 MHz, CDCl_3_) δ 1.73 (s, 3H), 5.20 (d, 1H, *J* = 15.6 Hz), 5.31 (d, 1H, *J* = 15.6 Hz), 5.45 (d, 1H, *J* = 14.8 Hz), 5.49 (d, 1H, *J* = 14.8 Hz), 7.16–7.28 (m, 3H), 7.32–7.39 (m, 3H), 7.54 (s, 1H), 7.67 (ddd, 1H, *J* = 8.3, 7.4, 1.6 Hz), 7.77 (d, 1H, *J* = 8.4 Hz), 7.96 (dd, 1H, *J* = 7.7, 1.5 Hz); ^13^C NMR (126 MHz, CDCl_3_) δ 23.6, 39.1, 54.5, 70.6, 116.6, 119.5, 123.5, 124.3, 128.3, 128.8, 129.0, 129.3, 134.3, 137.1, 141.4, 143.0, 169.8, 191.3; IR (cm^−1^): *ν* 3137, 3033, 2980, 2106, 1713, 1676, 1602, 1489, 1469, 1379, 1336, 1279, 1223, 765, 724; HRMS (ESI+): *m*/*z* calcd for C_20_H_18_N_7_O_2_^+^ [M + H]^+^ 388,1516, found 388.1514. Anal. Calcd for C_20_H_17_N_7_O_2_ (387.39): C, 62.01; H, 4.42; N, 25.31%. Found: C, 61.74; H, 4.77; N, 25.15%.

## 4. Conclusions

We have developed a methodology for accessing bis(1,2,3-triazole) functionalized quinoline-2,4-diones in which the triazole heterocycles are present in substituents at positions 1 and 3 of the quinoline scaffold. Preliminary investigation has revealed that these compounds are potential multidentate ligands for arene-ruthenium.

## Reference

## Figures and Tables

**Table 1:**
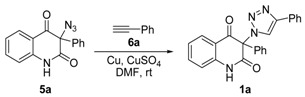
The Effect of Granular Copper to the Conversion of **5a** into **1a** ^a^.

Entry	Cu^0^ (mmol)	Reaction Time (h)	Yield ^b^ (%)
1	3.8	0.75	98
2	3	0.75	91
3	2	1	89
4	1	25	82 ^c^

^a^ Reaction conditions: **5a** (1 mmol), phenylacetylene (1 mmol), and CuSO_4_·5H_2_O (0.1 mmol), DMF (4 mL), rt. The reaction time was determined by thin-layer chromatography (TLC) monitoring of the reaction mixture. ^b^ Refers to the yield of isolated pure product. ^c^ Complete consumption of **5a** was not reached.

**Table 2:**
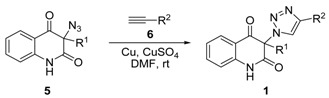
Preparation of compounds **1**.

Entry	Azide 5	R^1^	Acetylene 6	R^2^	Product 1	Yield ^a^
1	**5a**	Me	**6a**	Ph	**1a**	95
2	**5a**	Me	**6a**	Ph	**1a**	83 ^b^
3	**5b**	Ph	**6a**	Ph	**1b**	86
4	**5a**	Me	**6b**	CH_2_OH	**1c**	99
5	**5b**	Ph	**6b**	CH_2_OH	**1d**	98

^a^ Refers to percent yield of pure (by TLC and IR) isolated product. ^b^ Employing CuSO_4_∙5H_2_O/l-ascorbic acid/CH_2_Cl_2_/water conditions, 48 h reaction time.

**Table 3:**
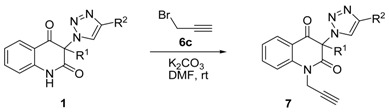
Preparation of compounds **7**.

Entry	1	R^1^	R^2^	6	Yield of 7 (%) ^a^
1	**1a**	Me	Ph	**6c**	**7a**, 96
2	**1b**	Ph	Ph	**6c**	**7b**, 79
3	**1e**	Me	CH_2_OAc	**6c**	**7c**, 81
4	**1f**	Ph	CH_2_OAc	**6c**	**7d**, 63

^a^ Refers to percent yield of pure (by TLC and IR) isolated product.

**Table 4:**
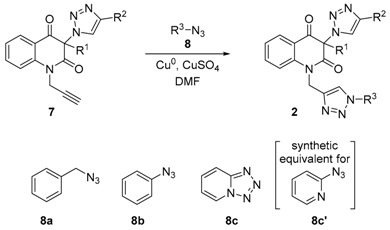
Preparation of compounds **2**.

Entry	2	R^1^	R^2^	R^3^	t (°C)	Time (h)	Yield ^a^ (%)
1	**a**	Me	Ph	Bn	23	1	97
2	**b**	Me	Ph	Ph	23	1	99
3	**c**	Me	Ph	2-Py	100	0.5	93
4	**d**	Me	CH_2_OAc	Bn	23	0.5	96
5	**d**	Me	CH_2_OAc	Bn	23	4	85 ^b^
6	**d**	Me	CH_2_OAc	Bn	23	48	81 ^c^
7	**d**	Me	CH_2_OAc	Bn	23	45	45 ^d^
8	**e**	Me	CH_2_OAc	Ph	23	2	92
9	**f**	Me	CH_2_OAc	2-Py	100	1	85
10	**g**	Ph	Ph	Bn	23	1	92
11	**h**	Ph	Ph	Ph	23	1	94
12	**i**	Ph	Ph	2-Py	100	0.75	57
13	**j**	Ph	CH_2_OAc	Bn	23	2	97
14	**k**	Ph	CH_2_OAc	Ph	23	0.5	93
15	**l**	Ph	CH_2_OAc	2-Py	100	0.5	85

^a^ Refers to percent yield of pure (by TLC and IR) isolated product. ^b^ Employing CH_2_Cl_2_/water/CuSO_4_∙5H_2_O/Na-ascorbate conditions. ^c^ Employing *t*-BuOH/water/CH_3_CN/CuSO_4_∙5H_2_O/Na-ascorbate conditions. ^d^ Employing *t*-BuOH/water/CuSO_4_∙5H_2_O/l-ascorbic acid conditions.

**Table 5:** Selected ^1^H, ^13^C and ^15^N NMR chemical shifts in ppm for compounds **1** and **7**.

	1a	1b	1c	1d	7a	7b	7c	7d
**Quinolone**								
N1	–	–	–	–	–	–	134.4	–
C2	168.5	166.8	168.7	166.8	167.7	165.8	167.8	165.8
C3	72.2	80.0	71.9	79.7	72.6	79.6	72.8	80.0
C4	190.7	188.9	190.8	189.0	189.7	187.5	189.6	187.7
C4a	117.4	119.2	117.5	119.2	119.0	121.0	119.2	120.9
C5	127.7	127.6	127.6	127.5	128.2	129.2	128.0	127.8
C6	123.5	123.5	123.3	123.4	124.2	124.6	124.0	124.2
C7	137.3	137.0	137.1	136.9	137.3	136.9	137.1	136.7
C8	117.0	116.7	116.9	116.7	116.7	115.8	116.6	116.3
C8a	141.6	140.5	141.6	140.6	140.8	140.6	140.7	140.0
								
Ring A								
N1^A^	–	–	–	–	–	–	247.9	–
N2^A^	–	–	–	–	–	–	363.4	–
N3^A^	–	–	–	–	–	–	354.0	–
C4^A^	145.8	145.3	147.4	146.8	145.9	146.0	141.5	140.9
C5^A^	122.4	123.4	123.7	124.8	122.5	122.3	126.0	127.1
H5^A^	8.89	8.49	8.26	7.77	8.89	7.26	8.46	8.15

**Table 6:** Selected ^1^H, ^13^C and ^15^N NMR chemical shifts in ppm for compounds **2**.

	2a	2b	2c	2d	2e	2f	2g	2h	2i	2j	2k	2l
**Quinolone**												
N1	–	136.3	135.8	138.7	138.7	135.3	–	–	137.5	140.4	140.4	138.9
C2	168.2	168.3	168.5	168.2	168.3	168.6	166.2	166.4	166.6	166.6	166.9	166.6
C3	72.8	73.0	73.0	71.6	71.5	73.3	80.1	80.3	80.4	79.6	79.6	79.7
C4	190.0	190.0	189.9	189.4	189.4	189.9	188.2	188.2	188.1	187.9	187.9	187.9
C4a	119.1	119.2	119.3	119.2	119.2	119.4	120.9	120.9	120.9	120.9	120.9	121.0
C5	128.1	128.1	128.1	129.3	129.4	127.9	127.9	127.9	127.9	129.0	129.1	129.1
C6	123.9	124.0	123.9	124.6	124.7	123.8	124.0	124.1	124.0	124.6	124.7	124.6
C7	137.2	137.3	137.2	137.8	137.8	137.0	136.8	136.8	136.8	137.2	137.4	137.2
C8	116.7	116.8	116.6	116.9	116.8	116.5	116.7	116.7	116.5	116.8	116.7	116.6
C8a	141.5	141.6	141.4	141.7	141.7	141.3	140.8	140.7	140.5	141.1	140.9	141.2
												
Ring A												
N1^A^	–	248.9	248.9	248.4	248.8	247.6	–	–	248.7	249.8	249.9	249.7
N2^A^	–	363.2	363.4	361.6	–	363.7	–	–	367.4	365.1	–	–
N3^A^	–	347.1	347.1	355.2	355.5	353.4	–	–	347.2	356.9	357.2	357.1
C4^A^	145.9	146.0	146.0	142.3	142.3	141.6	145.4	145.4	145.4	140.9	140.9	140.9
C5^A^	122.5	122.5	122.5	124.2	124.1	126.1	123.4	123.5	123.4	126.4	126.4	126.4
H5^A^	8.87	8.87	8.87	7.78	7.86	8.47	8.51	8.54	8.58	7.08	7.14	7.13
												
Ring D												
N1^D^	–	255.7	260.5	250.4	256.3	260.0	–	–	260.4	250.4	256.3	261.2
N2^D^	–	358.1	358.6	362.6	–	361.9	–	–	–	362.9	–	–
N3^D^	–	353.4	356.9	350.0	351.9	356.5	–	–	357.7	350.5	352.9	355.8
C4^D^	142.2	143.3	143.2	142.9	143.2	143.2	141.9	142.9	143.0	142.9	143.2	143.0
C5^D^	123.8	121.8	120.6	123.5	121.7	120.6	124.2	122.3	120.8	123.5	121.8	121.0
H5^D^	8.16	8.75	8.82	7.55	8.10	8.82	8.24	8.83	8.81	7.58	8.05	8.63

## Supplementary Materials

The following are available online: ^1^H NMR and ^13^C NMR spectra of all new compounds.
