# Supplementary material for: Synthesis of Bis(1,2,3-Triazole) Functionalized Quinoline-2,4-Diones
Source: Molecules. 2018 Sep 10;23(9):2310. doi: 10.3390/molecules23092310 (PMC6225383; doi:10.3390/molecules23092310)
Supplement: Supplementary file 1 [file molecules-23-02310-s001.pdf]

Supplementary Material

Synthesis of bis(1,2,3-triazole) functionalized quinoline-2,4-diones

David Milićević [1], Roman Kimmel [1], Martin Gazvoda [2], Damijana Urankar [2], Stanislav Kafka [1,*] and Janez Košmrlj [2,*]

[1] Department of Chemistry, Faculty of Technology, Tomas Bata University in Zlin, 760 01 Zlin, Czech Republic
[2] Faculty of Chemistry and Chemical Technology, University of Ljubljana, SI-1000 Ljubljana, Slovenia

Table of Contents

^{1}H and ^{13}C NMR spectra of compound **1a** S2
^{1}H and ^{13}C NMR spectra of compound **1b** S3
^{1}H and ^{13}C NMR spectra of compound **1c** S4
^{1}H and ^{13}C NMR spectra of compound **1d** S5
^{1}H and ^{13}C NMR spectra of compound **1e** S6
^{1}H and ^{13}C NMR spectra of compound **1f** S7
^{1}H and ^{13}C NMR spectra of compound **2a** S8
^{1}H and ^{13}C NMR spectra of compound **2b** S9
^{1}H and ^{13}C NMR spectra of compound **2c** S10
^{1}H and ^{13}C NMR spectra of compound **2d** S11
^{1}H and ^{13}C NMR spectra of compound **2e** S12
^{1}H and ^{13}C NMR spectra of compound **2f** S13
^{1}H and ^{13}C NMR spectra of compound **2g** S14
^{1}H and ^{13}C NMR spectra of compound **2h** S15
^{1}H and ^{13}C NMR spectra of compound **2i** S16
^{1}H and ^{13}C NMR spectra of compound **2j** S17
^{1}H and ^{13}C NMR spectra of compound **2k** S18
^{1}H and ^{13}C NMR spectra of compound **2l** S19
^{1}H and ^{13}C NMR spectra of compound **4a** S20
^{1}H and ^{13}C NMR spectra of compound **4b** S21
^{1}H and ^{13}C NMR spectra of compound **5a** S22
^{1}H and ^{13}C NMR spectra of compound **5b** S23
^{1}H and ^{13}C NMR spectra of compound **7a** S24
^{1}H and ^{13}C NMR spectra of compound **7b** S25
^{1}H and ^{13}C NMR spectra of compound **7c** S26
^{1}H and ^{13}C NMR spectra of compound **7d** S27
^{1}H and ^{13}C NMR spectra of compound **9a** S28
^{1}H and ^{13}C NMR spectra of compound **10a** S29

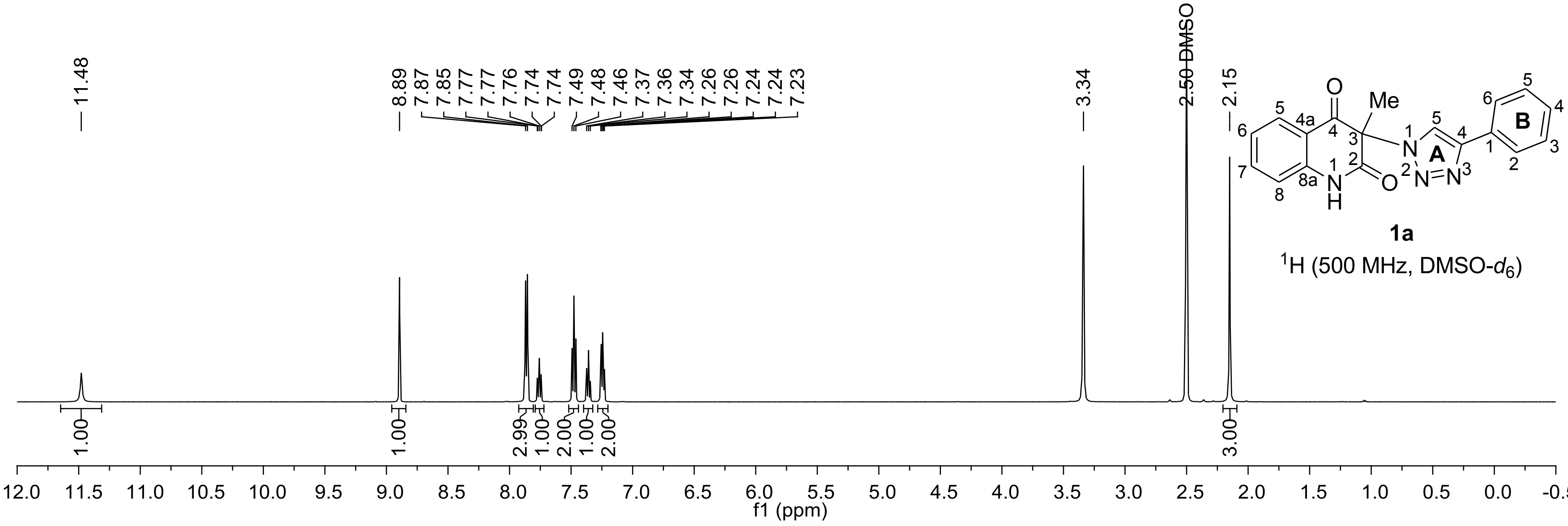
11.48
8.89
7.87
7.85
7.77
7.77
7.76
7.74
7.74
7.49
7.48
7.46
7.37
7.36
7.34
7.26
7.26
7.24
7.24
7.23
3.34
2.50 DMSO
2.15
1a
^{1}H (500 MHz, DMSO-d_6)
1.00
1.00
2.99
1.00
2.00
1.00
2.00
3.00
f1 (ppm)

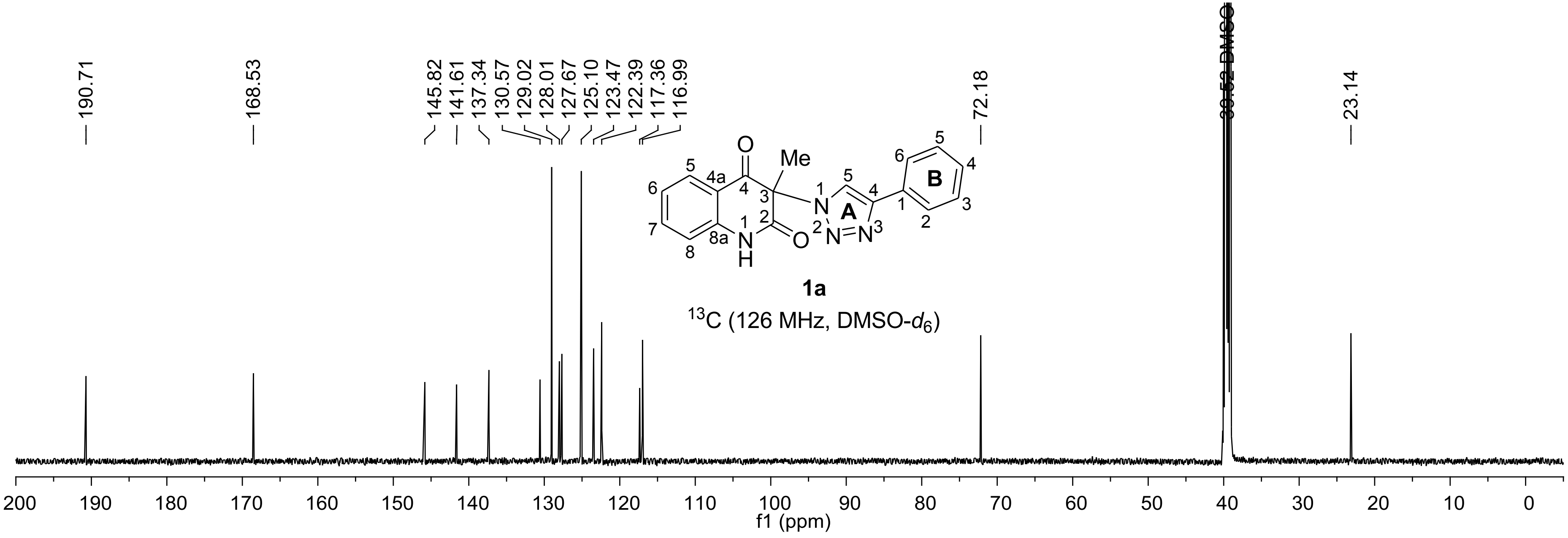
190.71
168.53
145.82
141.61
137.34
130.57
129.02
128.01
127.67
125.10
123.47
122.39
117.36
116.99
72.18
39.52 DMSO
23.14
1a
^{13}C (126 MHz, DMSO-d_6)
f1 (ppm)

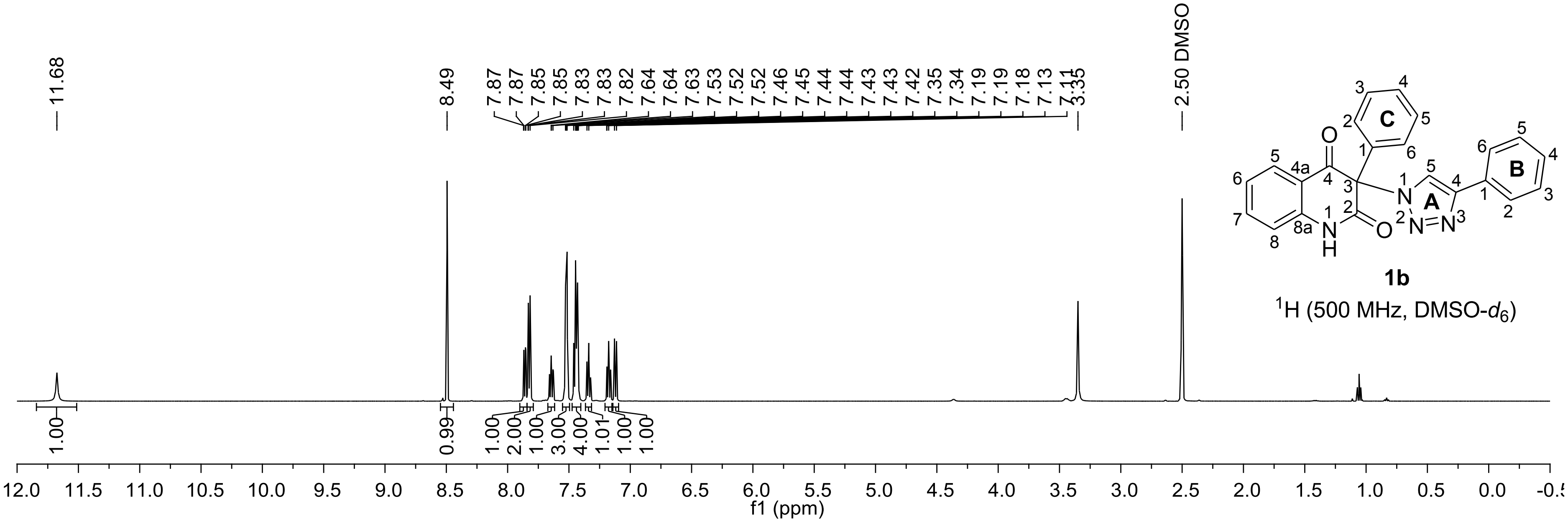
11.68
8.49
7.87 7.87 7.85 7.85 7.83 7.83 7.82 7.64 7.64 7.63 7.53 7.52 7.52 7.46 7.45 7.44 7.44 7.43 7.43 7.42 7.35 7.34 7.19 7.19 7.18 7.13 7.11
3.35
2.50 DMSO
1.00 0.99 1.00 2.00 1.00 3.00 4.00 1.01 1.00 1.00
f1 (ppm)
1b
^{1}H (500 MHz, DMSO-d_6)

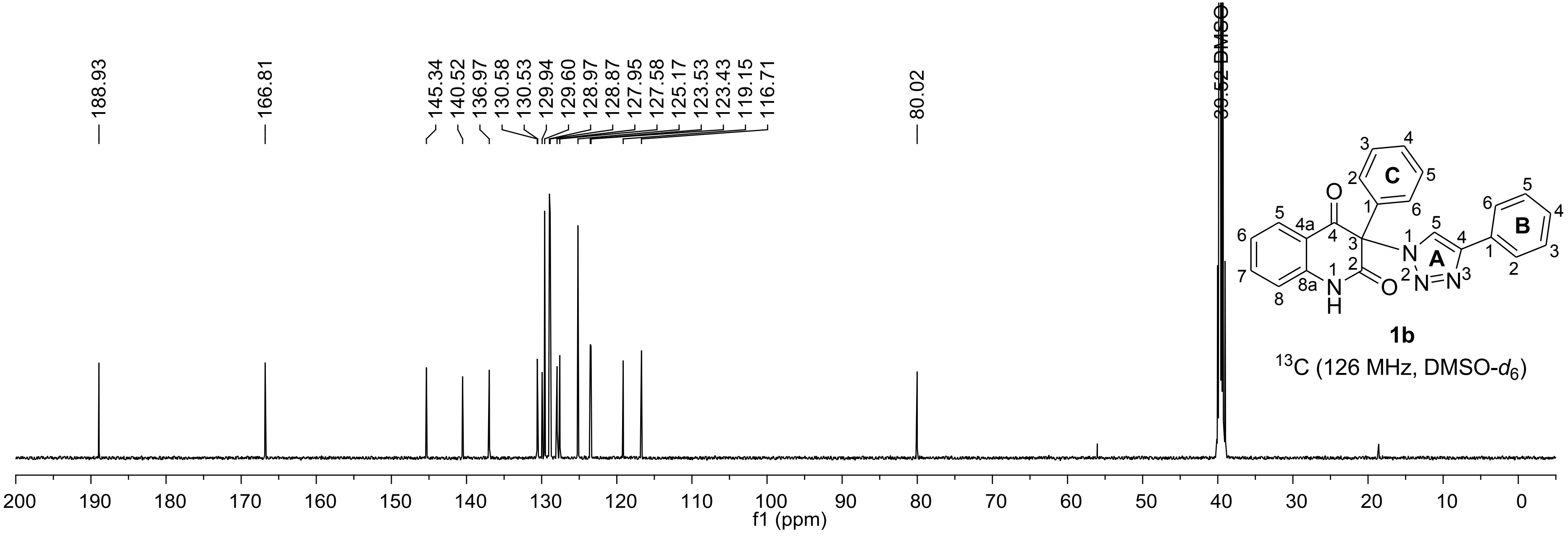
188.93
166.81
145.34 140.52 136.97 130.58 130.53 129.94 129.60 128.97 128.87 127.95 127.58 125.17 123.53 123.43 119.15 116.71
80.02
39.52 DMSO
f1 (ppm)
1b
^{13}C (126 MHz, DMSO-d_6)

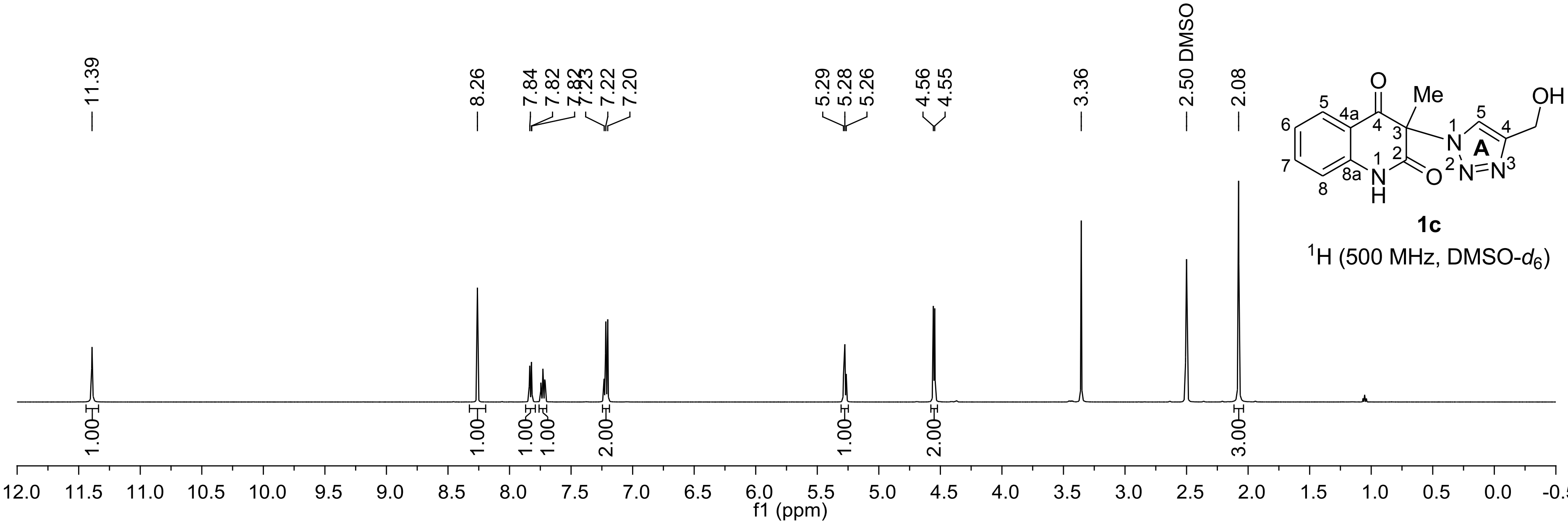
11.39
8.26
7.84
7.82
7.82
7.23
7.22
7.20
5.29
5.28
5.26
4.56
4.55
3.36
2.50 DMSO
2.08
1.00
1.00
1.00
1.00
2.00
1.00
2.00
3.00
12.0 11.5 11.0 10.5 10.0 9.5 9.0 8.5 8.0 7.5 7.0 6.5 6.0 5.5 5.0 4.5 4.0 3.5 3.0 2.5 2.0 1.5 1.0 0.5 0.0
f1 (ppm)
O
Me
OH
5
4a
4
3
1
N
A
2
N=N
3
6
7
8
8a
1
N
H
O
1c
^{1}H (500 MHz, DMSO-d_6)

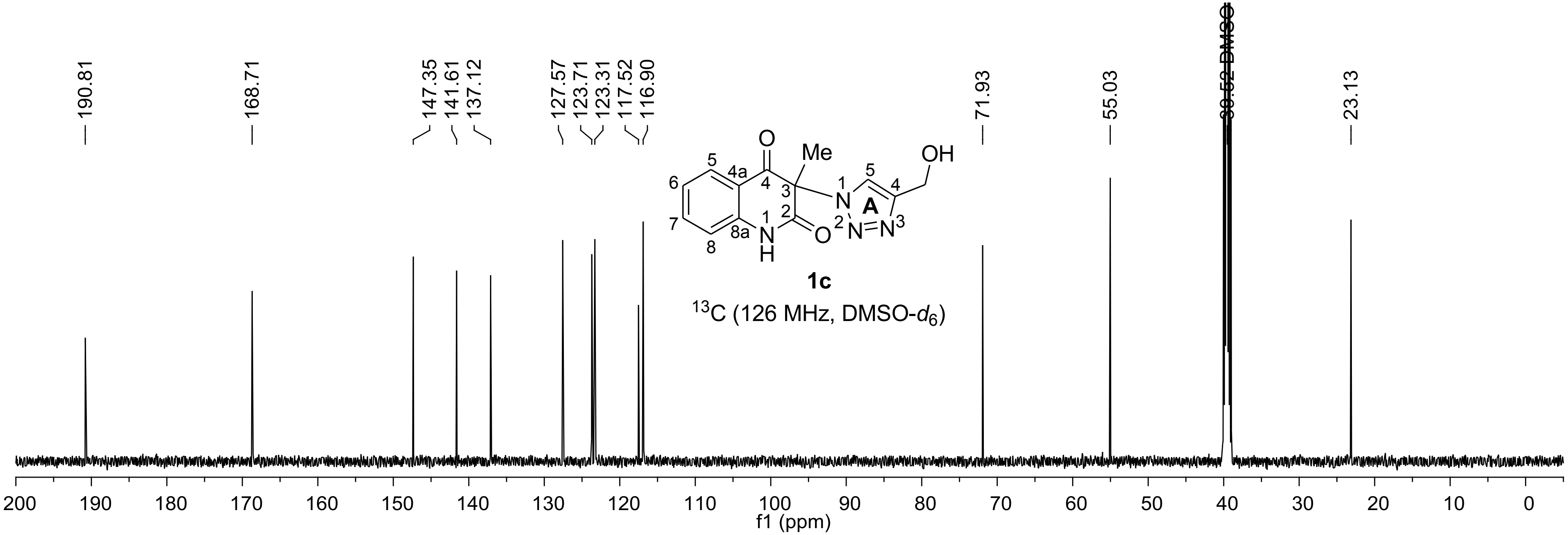
190.81
168.71
147.35
141.61
137.12
127.57
123.71
123.31
117.52
116.90
71.93
55.03
39.52 DMSO
23.13
200 190 180 170 160 150 140 130 120 110 100 90 80 70 60 50 40 30 20 10 0
f1 (ppm)
O
Me
OH
5
4a
4
3
1
N
A
2
N=N
3
6
7
8
8a
1
N
H
O
1c
^{13}C (126 MHz, DMSO-d_6)

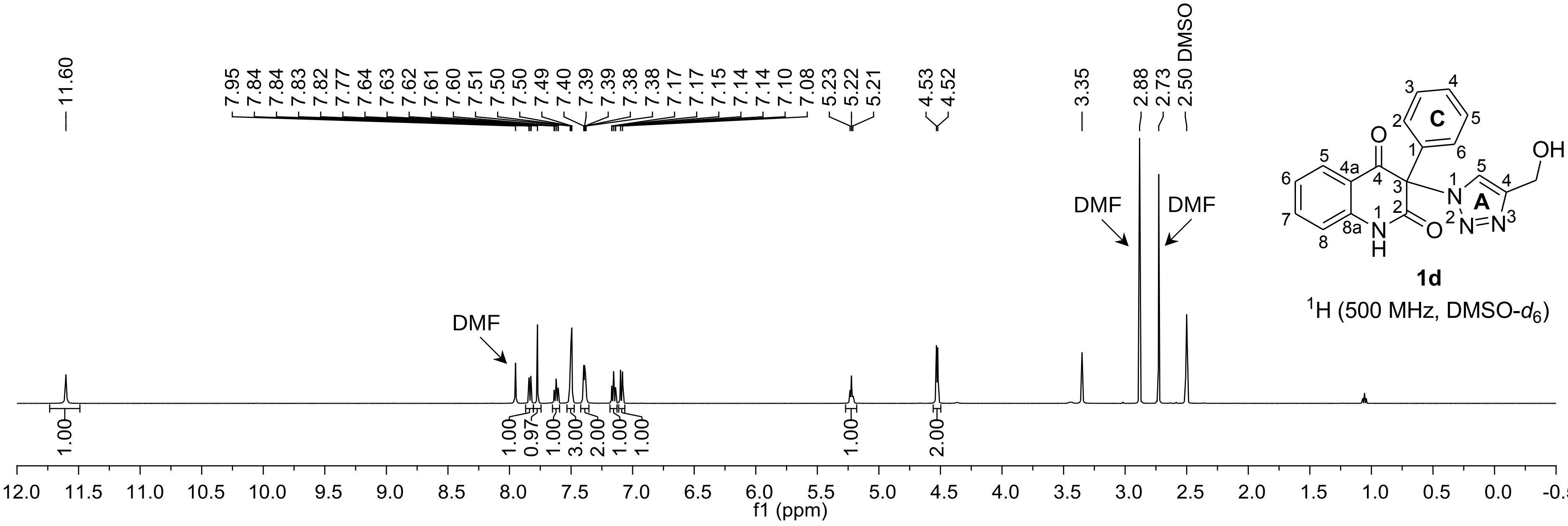
11.60
7.95 7.84 7.84 7.83 7.82 7.77 7.64 7.63 7.62 7.61 7.60 7.51 7.50 7.50 7.49 7.40 7.39 7.39 7.38 7.38 7.17 7.17 7.15 7.14 7.14 7.10 7.08
5.23 5.22 5.21
4.53 4.52
3.35
2.88 2.73 2.50 DMSO
DMF
DMF
DMF
1.00
1.00 0.97 1.00 3.00 2.00 1.00 1.00
1.00
2.00
f1 (ppm)
1d
^{1}H (500 MHz, DMSO-d_6)

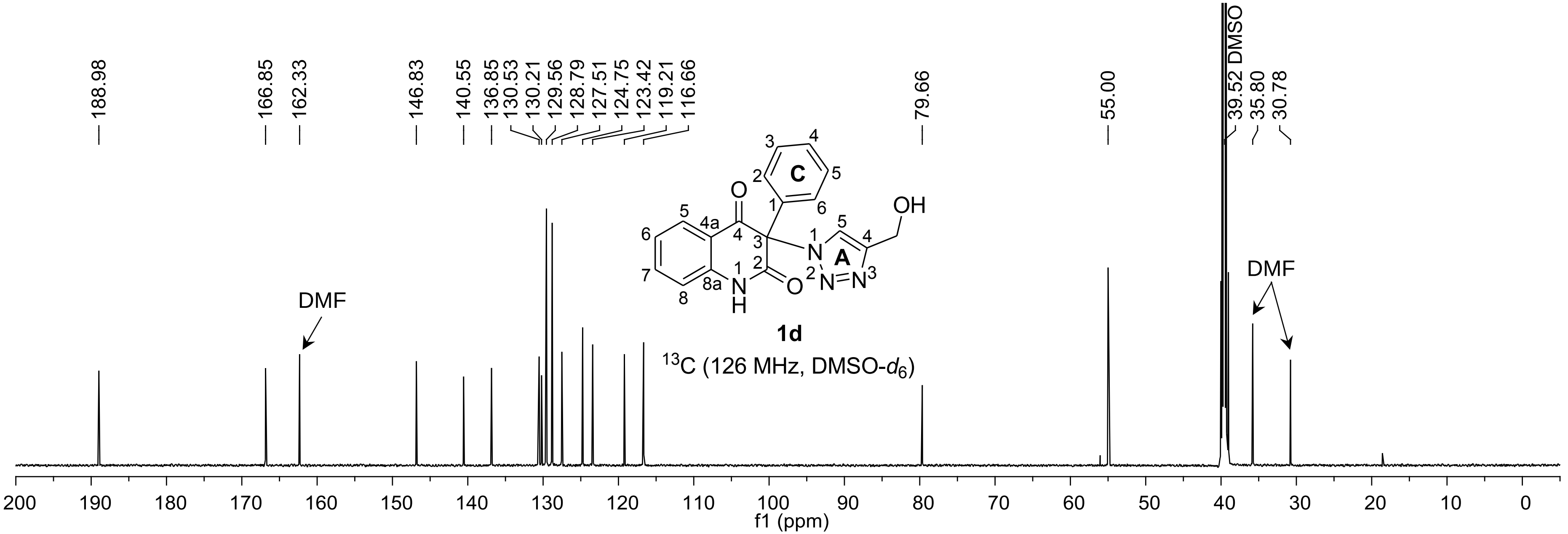
188.98
166.85 162.33
146.83
140.55 136.85 130.53 130.21 129.56 128.79 127.51 124.75 123.42 119.21 116.66
79.66
55.00
39.52 DMSO 35.80 30.78
DMF
DMF
f1 (ppm)
1d
^{13}C (126 MHz, DMSO-d_6)

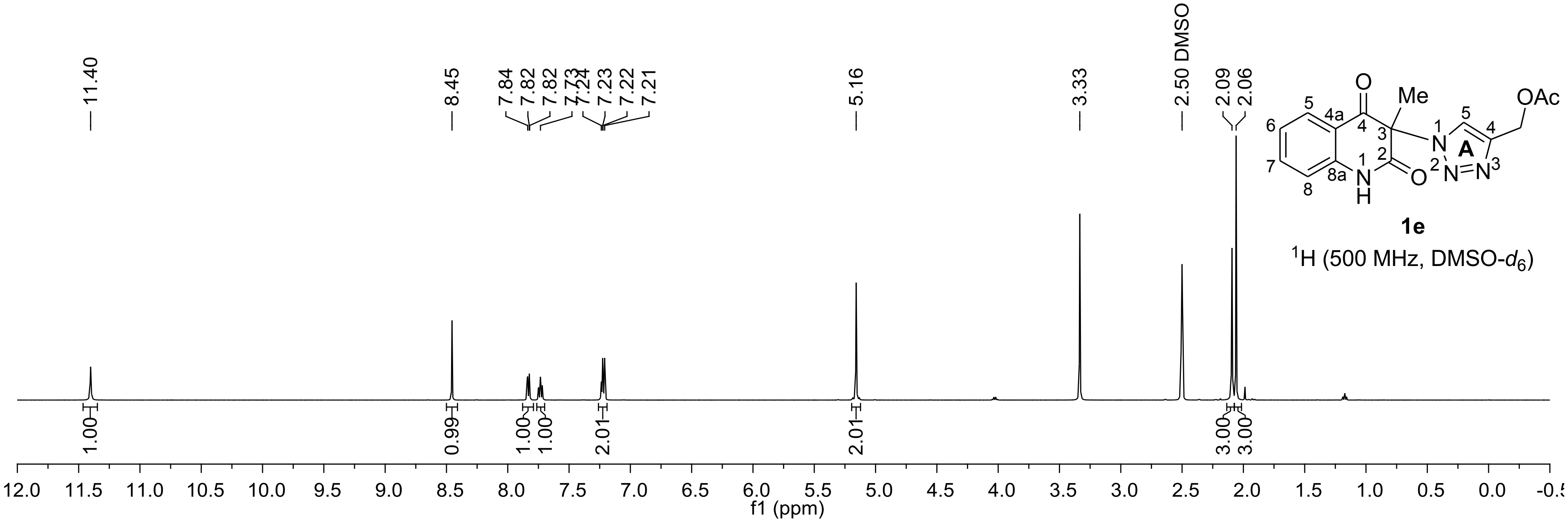
11.40
8.45
7.84
7.82
7.82
7.73
7.24
7.23
7.22
7.21
5.16
3.33
2.50 DMSO
2.09
2.06
1.00
0.99
1.00
1.00
2.01
2.01
3.00
3.00
12.0 11.5 11.0 10.5 10.0 9.5 9.0 8.5 8.0 7.5 7.0 6.5 6.0 5.5 5.0 4.5 4.0 3.5 3.0 2.5 2.0 1.5 1.0 0.5 0.0
f1 (ppm)
O
Me
OAc
5
4a
4
3
1
5
4
A
2
3
6
7
8
8a
N
H
O
1e
1H (500 MHz, DMSO-d6)

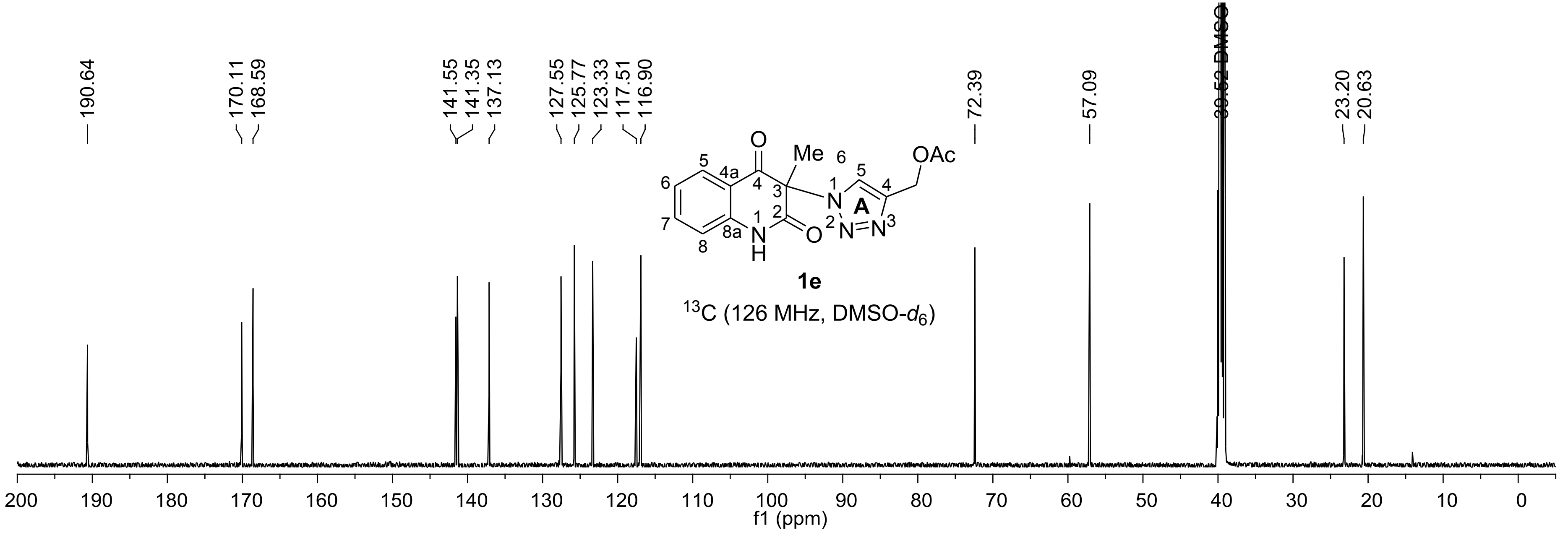
190.64
170.11
168.59
141.55
141.35
137.13
127.55
125.77
123.33
117.51
116.90
72.39
57.09
DMSO
23.20
20.63
O
Me
6
5
OAc
4a
4
3
1
4
A
2
3
6
7
8
8a
N
H
O
1e
13C (126 MHz, DMSO-d6)
200 190 180 170 160 150 140 130 120 110 100 90 80 70 60 50 40 30 20 10 0
f1 (ppm)

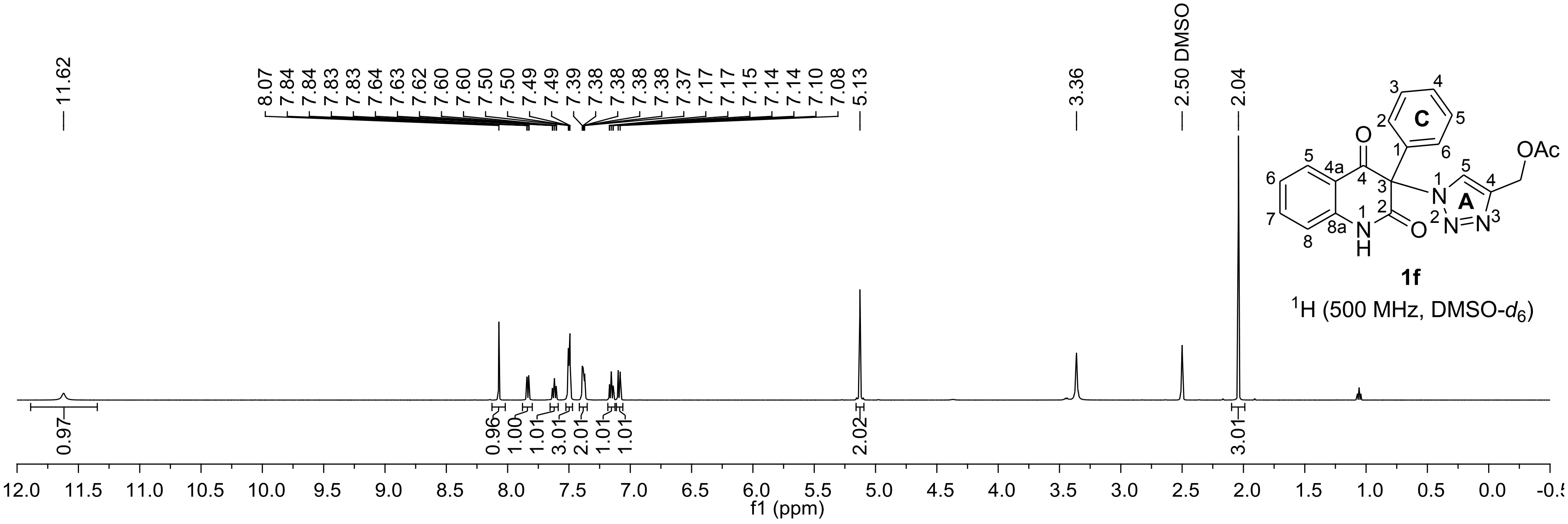
11.62
8.07 7.84 7.84 7.83 7.83 7.64 7.63 7.62 7.60 7.60 7.50 7.50 7.49 7.49 7.39 7.38 7.38 7.38 7.38 7.37 7.17 7.17 7.15 7.14 7.14 7.10 7.08
5.13
3.36
2.50 DMSO
2.04
0.97
0.96 1.00 1.01 3.01 2.01 1.01 1.01
2.02
3.01
OAc
1f
1H (500 MHz, DMSO-d6)
f1 (ppm)

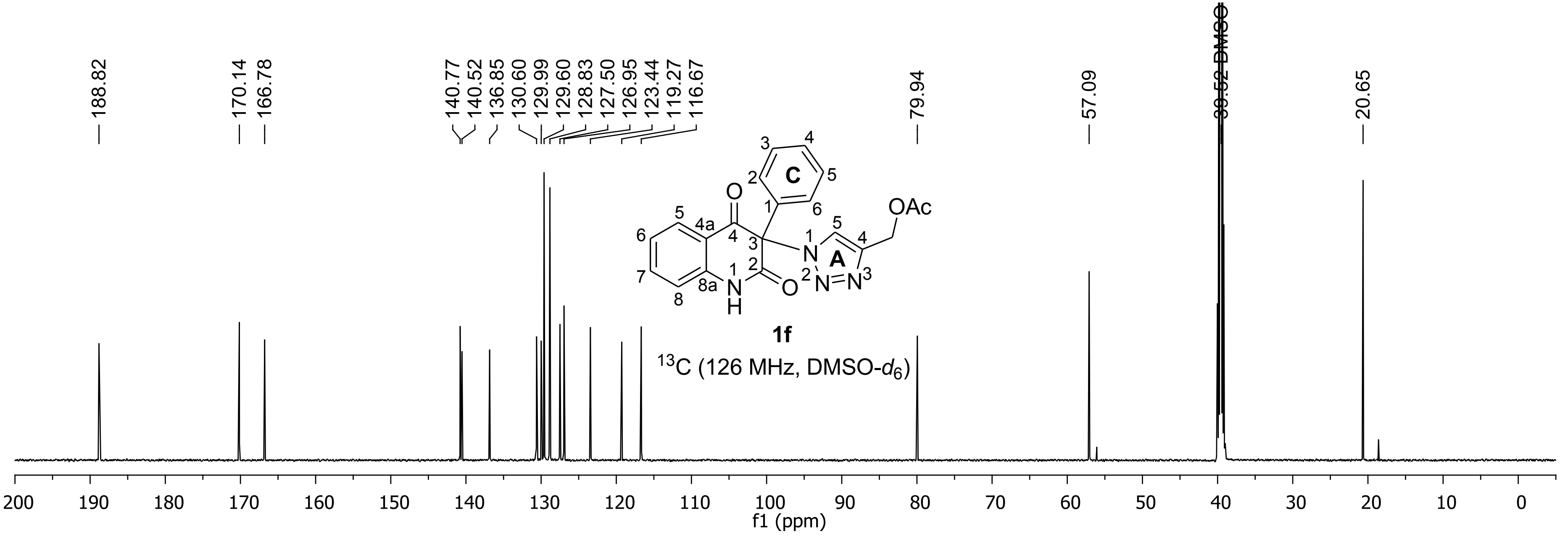
188.82
170.14 166.78
140.77 140.52 136.85 130.60 129.99 129.60 128.83 127.50 126.95 123.44 119.27 116.67
79.94
57.09
39.52 DMSO
20.65
OAc
1f
13C (126 MHz, DMSO-d6)
f1 (ppm)

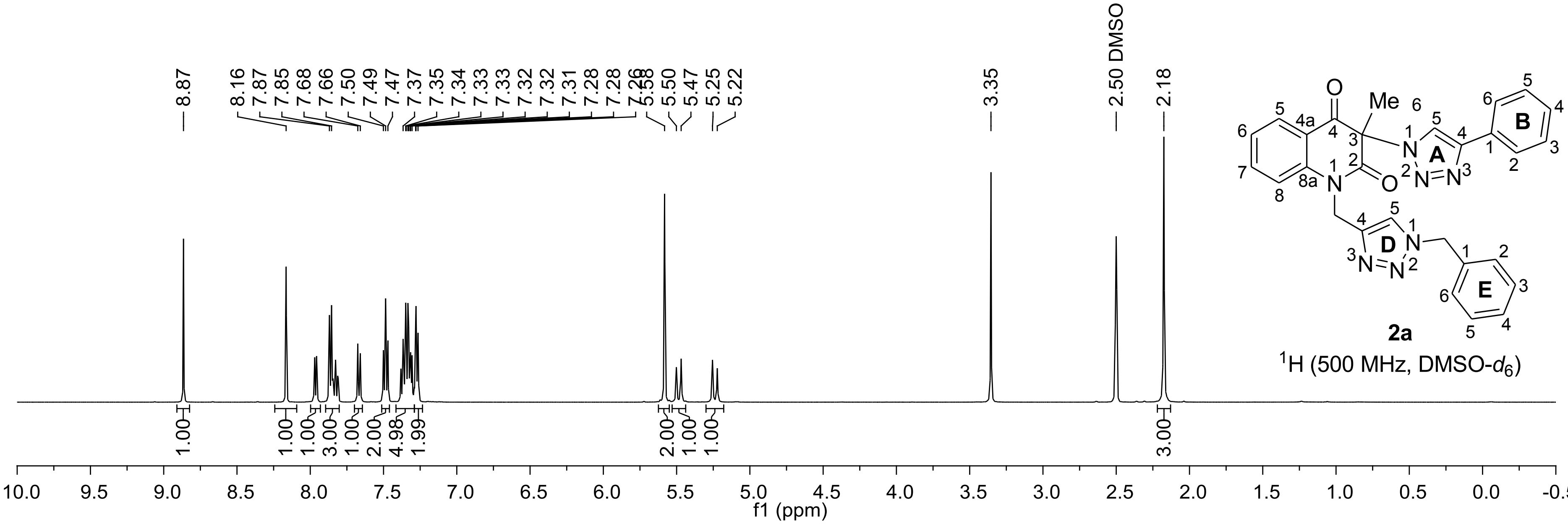
8.87
8.16
7.87
7.85
7.68
7.66
7.50
7.49
7.47
7.37
7.35
7.34
7.33
7.33
7.32
7.32
7.31
7.28
7.28
7.26
5.58
5.50
5.47
5.25
5.22
3.35
2.50 DMSO
2.18
1.00
1.00
1.00
3.00
1.00
2.00
4.98
1.99
2.00
1.00
1.00
3.00
f1 (ppm)
Me
2a
^{1}H (500 MHz, DMSO-d_6)

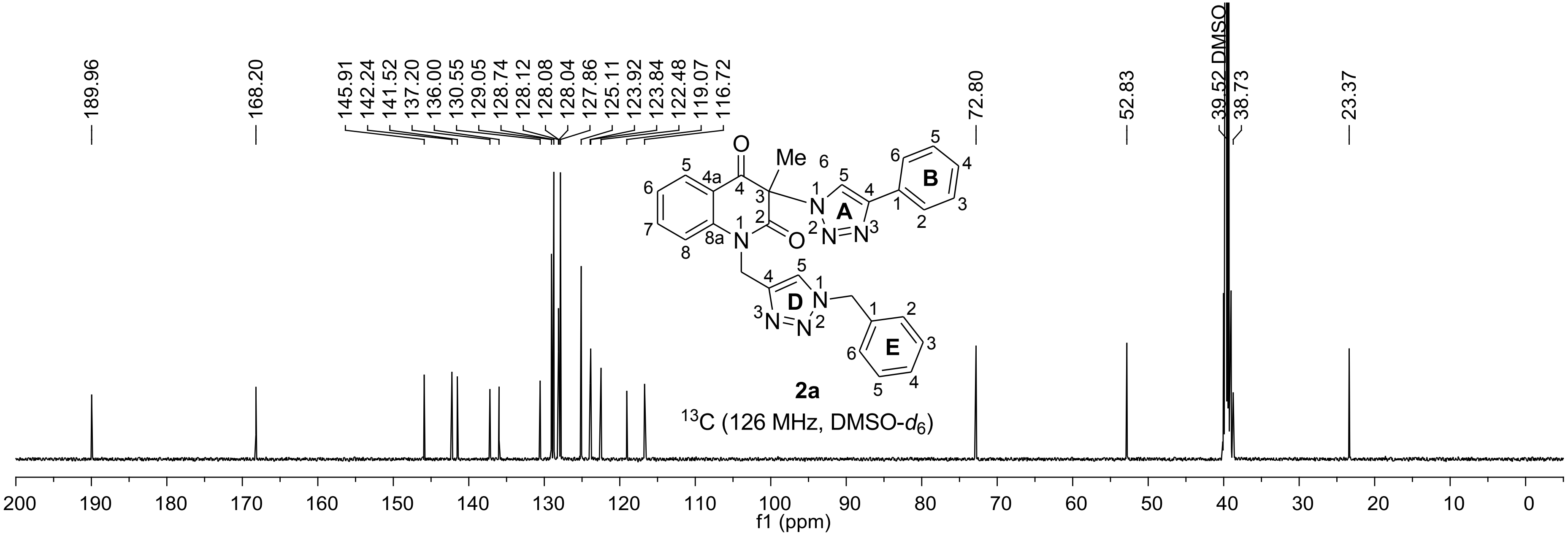
189.96
168.20
145.91
142.24
141.52
137.20
136.00
130.55
129.05
128.74
128.12
128.08
128.04
127.86
125.11
123.92
123.84
122.48
119.07
116.72
72.80
52.83
39.52 DMSO
38.73
23.37
f1 (ppm)
Me
2a
^{13}C (126 MHz, DMSO-d_6)

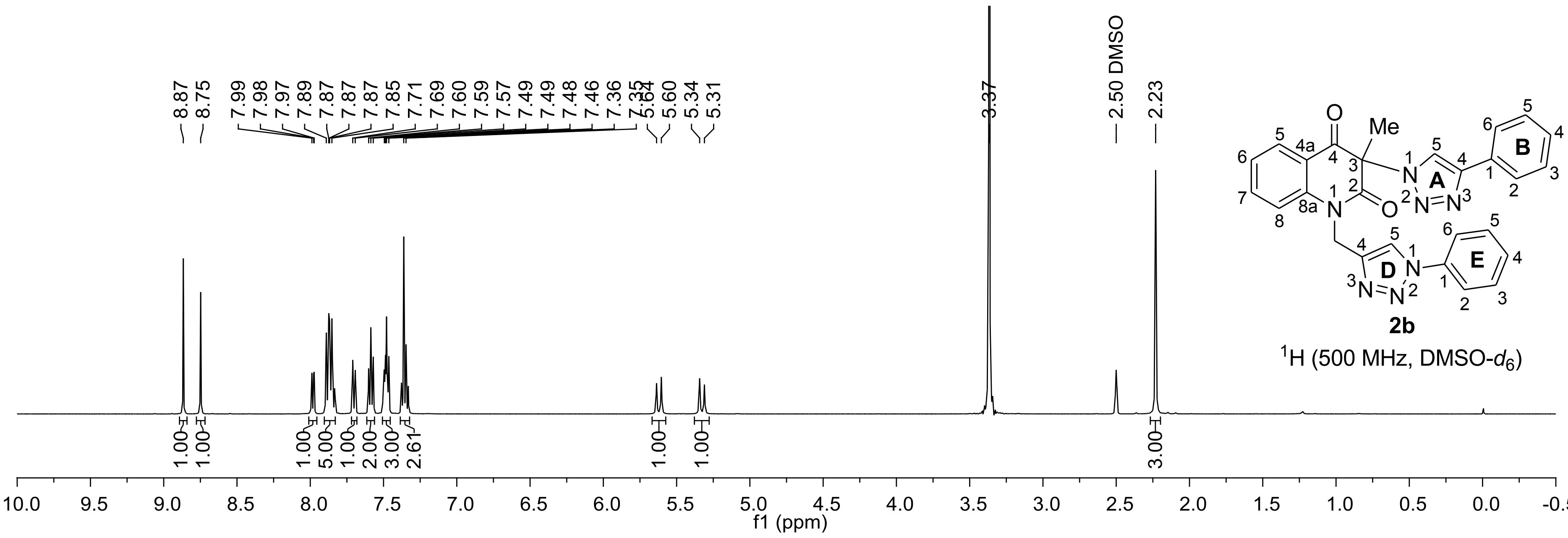
8.87
8.75
7.99
7.98
7.97
7.89
7.87
7.87
7.87
7.85
7.71
7.69
7.60
7.59
7.57
7.49
7.49
7.48
7.46
7.36
5.64
5.60
5.34
5.31
3.37
2.50 DMSO
2.23
1.00
1.00
1.00
5.00
1.00
2.00
3.00
2.61
1.00
1.00
3.00
f1 (ppm)
Me
A
B
D
E
2b
^{1}H (500 MHz, DMSO-d_6)

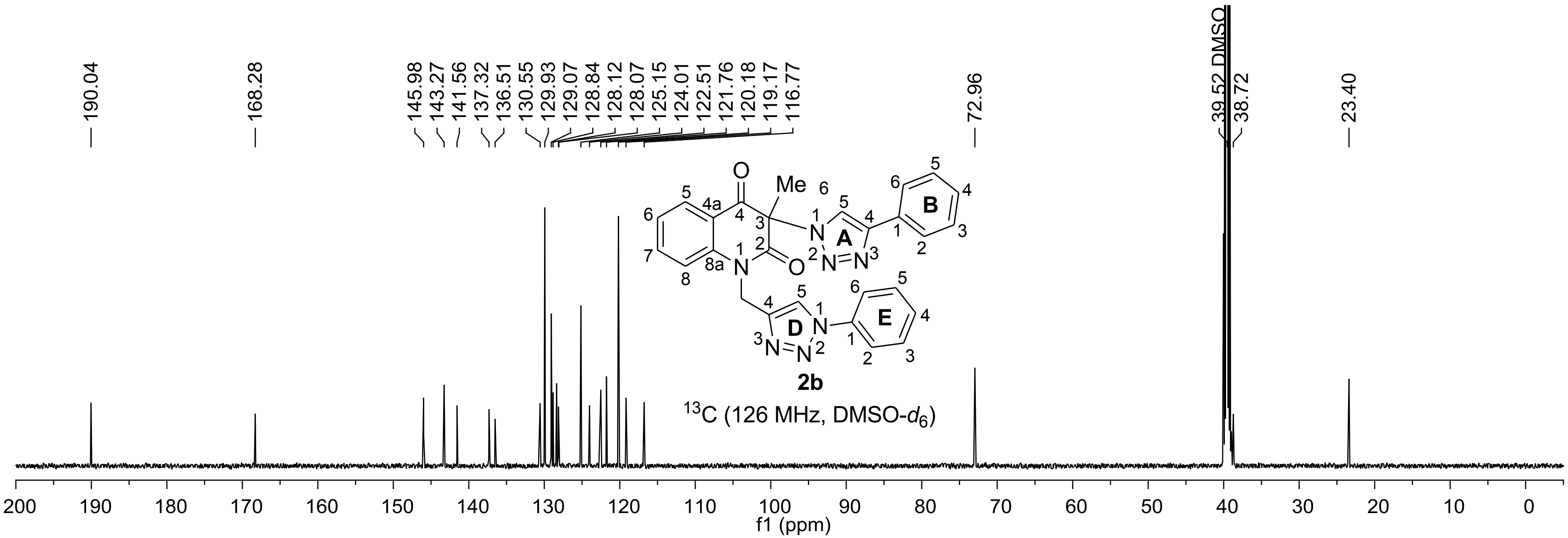
190.04
168.28
145.98
143.27
141.56
137.32
136.51
130.55
129.93
129.07
128.84
128.12
128.07
125.15
124.01
122.51
121.76
120.18
119.17
116.77
72.96
39.52 DMSO
38.72
23.40
f1 (ppm)
Me
A
B
D
E
2b
^{13}C (126 MHz, DMSO-d_6)

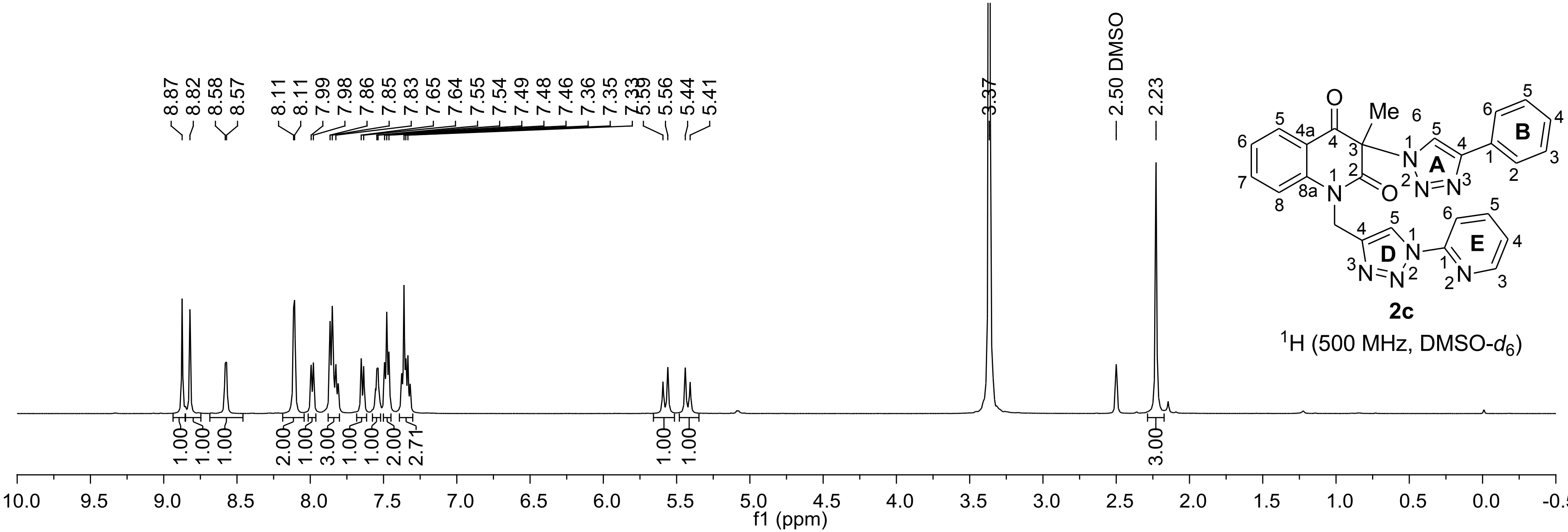
8.87
8.82
8.58
8.57
8.11
8.11
7.99
7.98
7.86
7.85
7.83
7.65
7.64
7.55
7.54
7.49
7.48
7.46
7.36
7.35
7.33
5.59
5.56
5.44
5.41
3.37
2.50 DMSO
2.23
1.00
1.00
1.00
2.00
1.00
3.00
1.00
1.00
2.00
2.71
1.00
1.00
3.00
f1 (ppm)
2c
¹H (500 MHz, DMSO-d6)

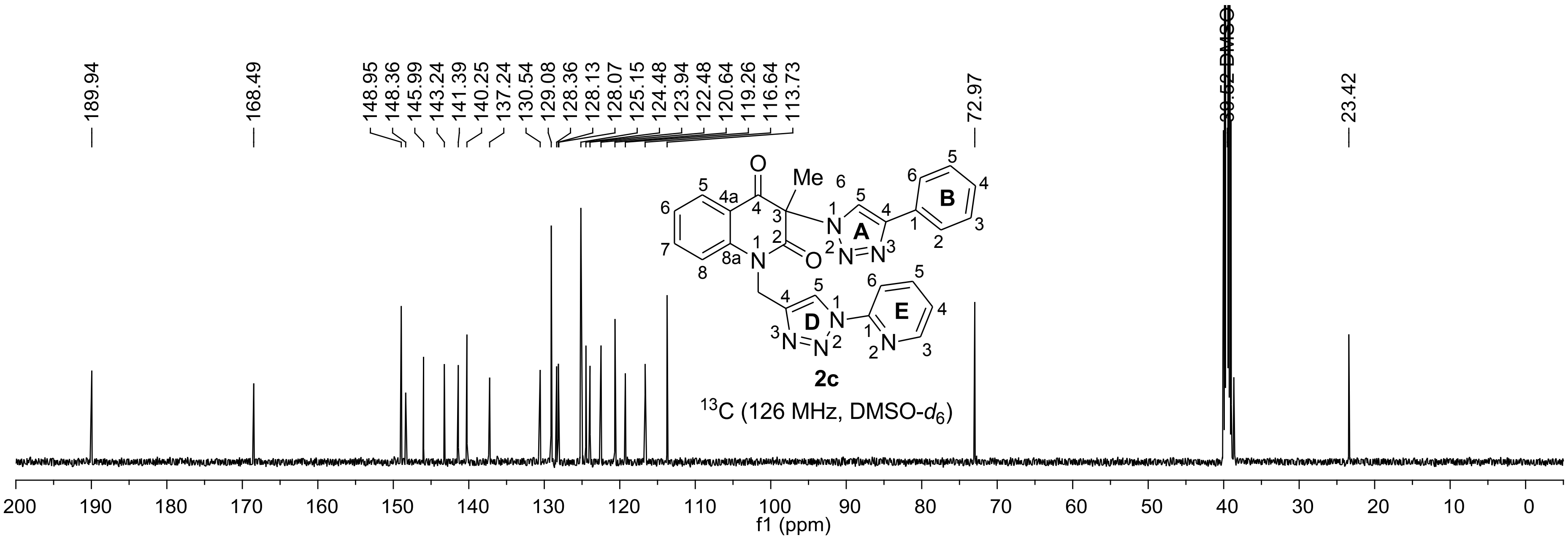
189.94
168.49
148.95
148.36
145.99
143.24
141.39
140.25
137.24
130.54
129.08
128.36
128.13
128.07
125.15
124.48
123.94
122.48
120.64
119.26
116.64
113.73
72.97
39.52 DMSO
23.42
f1 (ppm)
2c
¹³C (126 MHz, DMSO-d6)

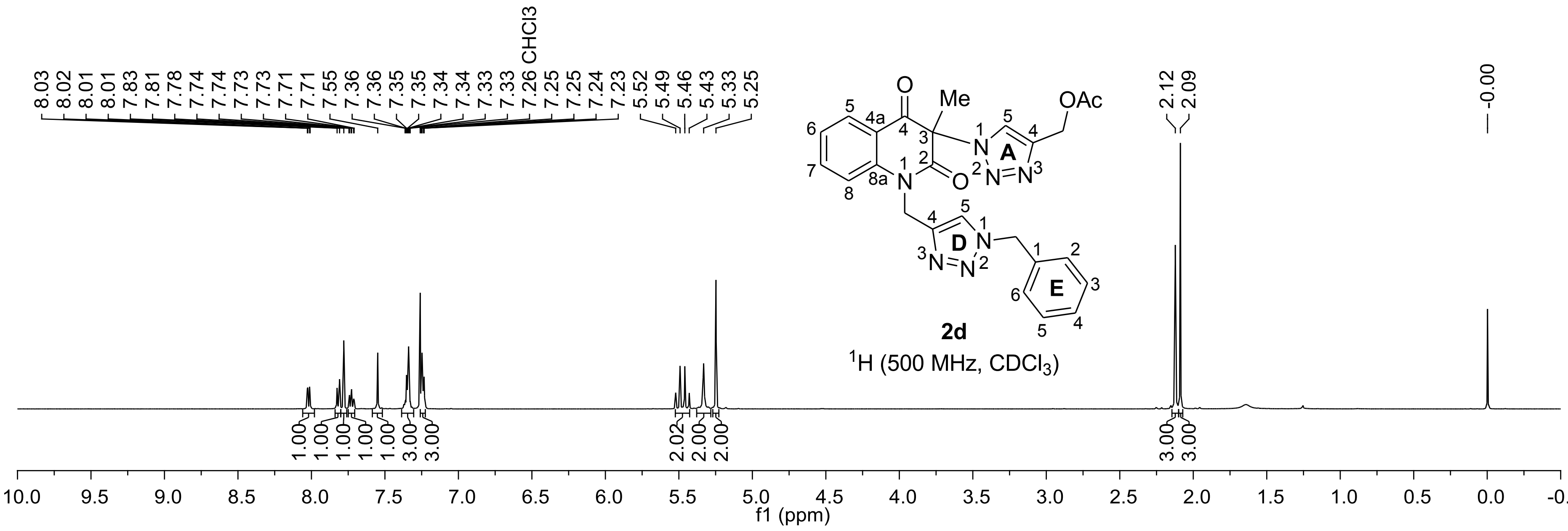
8.03 8.02 8.01 8.01 7.83 7.81 7.78 7.74 7.74 7.73 7.73 7.71 7.71 7.55 7.36 7.36 7.35 7.35 7.34 7.34 7.33 7.33 7.26 CHCl3 7.25 7.25 7.24 7.23 5.52 5.49 5.46 5.43 5.33 5.25
2.12 2.09
-0.00
1.00 1.00 1.00 1.00 1.00 3.00 3.00 2.02 2.00 2.00 3.00 3.00
Me
OAc
A
D
E
2d
^{1}H (500 MHz, $CDCl_3$)
f1 (ppm)

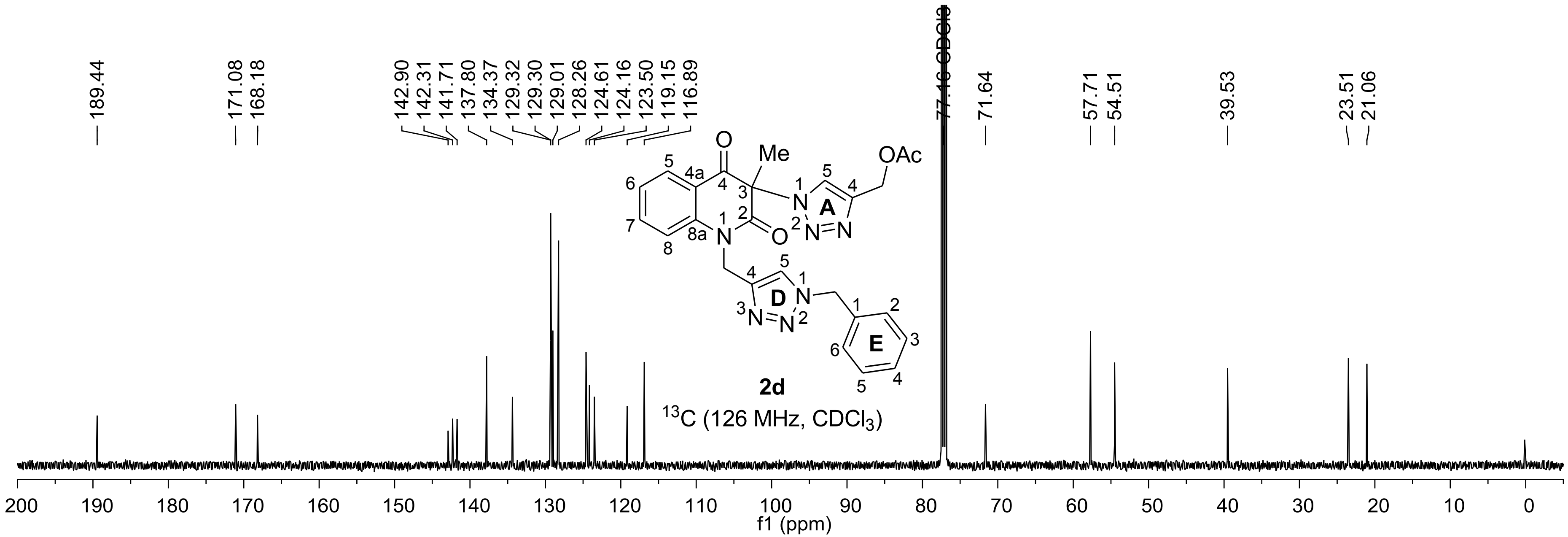
189.44
171.08 168.18
142.90 142.31 141.71 137.80 134.37 129.32 129.30 129.01 128.26 124.61 124.16 123.50 119.15 116.89
77.16 CDCl3
71.64
57.71 54.51
39.53
23.51 21.06
Me
OAc
A
D
E
2d
^{13}C (126 MHz, $CDCl_3$)
f1 (ppm)

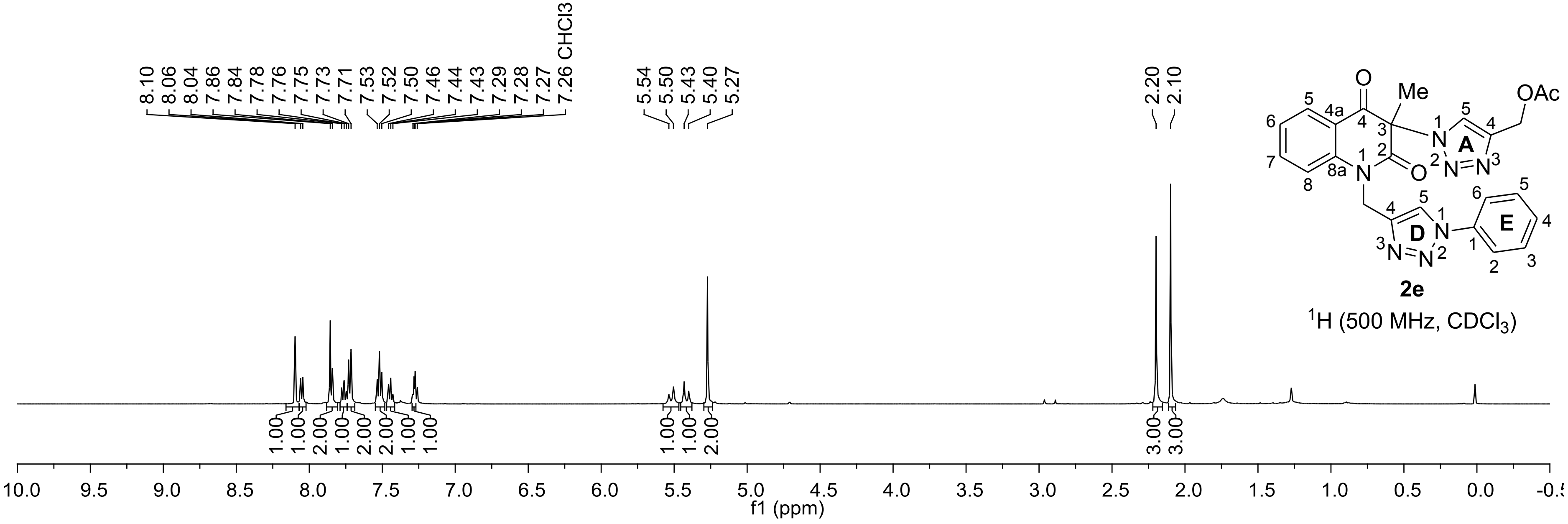
8.10 8.06 8.04 7.86 7.84 7.78 7.76 7.75 7.73 7.71 7.53 7.52 7.50 7.46 7.44 7.43 7.29 7.28 7.27 7.26 CHCl3
5.54 5.50 5.43 5.40 5.27
2.20 2.10
1.00 1.00 2.00 1.00 2.00 2.00 1.00 1.00
1.00 1.00 2.00
3.00 3.00
f1 (ppm)
O
Me
OAc
A
D
E
2e
^{1}H (500 MHz, $CDCl_3$)

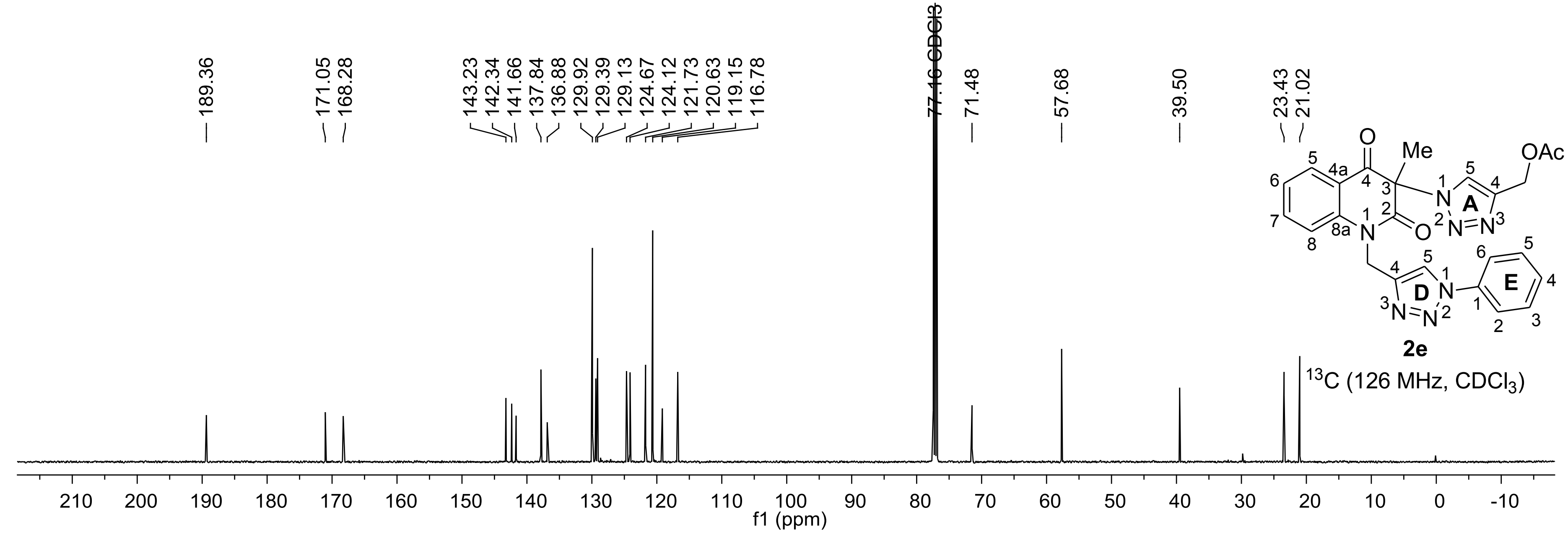
189.36
171.05 168.28
143.23 142.34 141.66 137.84 136.88 129.92 129.39 129.13 124.67 124.12 121.73 120.63 119.15 116.78
77.16 CDCl3
71.48
57.68
39.50
23.43 21.02
f1 (ppm)
O
Me
OAc
A
D
E
2e
^{13}C (126 MHz, $CDCl_3$)

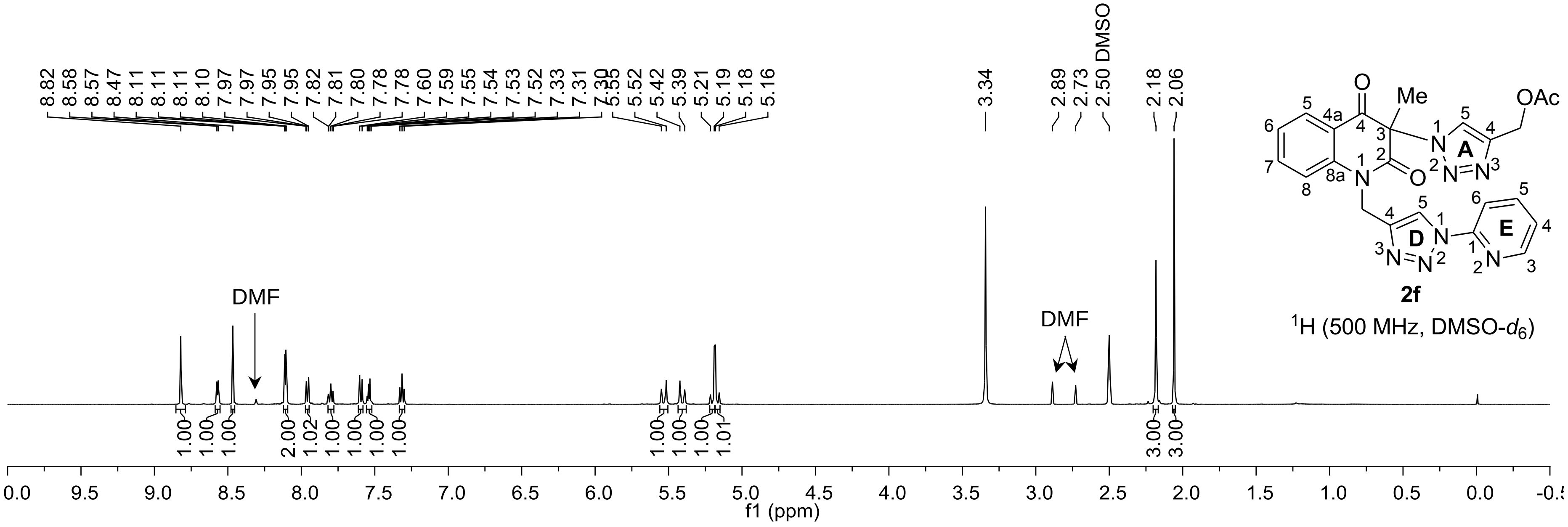
8.82 8.58 8.57 8.47 8.11 8.11 8.10 7.97 7.97 7.95 7.95 7.82 7.81 7.80 7.78 7.78 7.60 7.59 7.55 7.54 7.53 7.52 7.33 7.31 7.30 5.55 5.52 5.42 5.39 5.21 5.19 5.18 5.16
3.34
2.89 2.73 2.50 DMSO
2.18 2.06
DMF
DMF
1.00 1.00 1.00 2.00 1.02 1.00 1.00 1.00 1.00
1.00 1.00 1.00 1.01
3.00 3.00
f1 (ppm)
2f
^{1}H (500 MHz, DMSO-d_6)

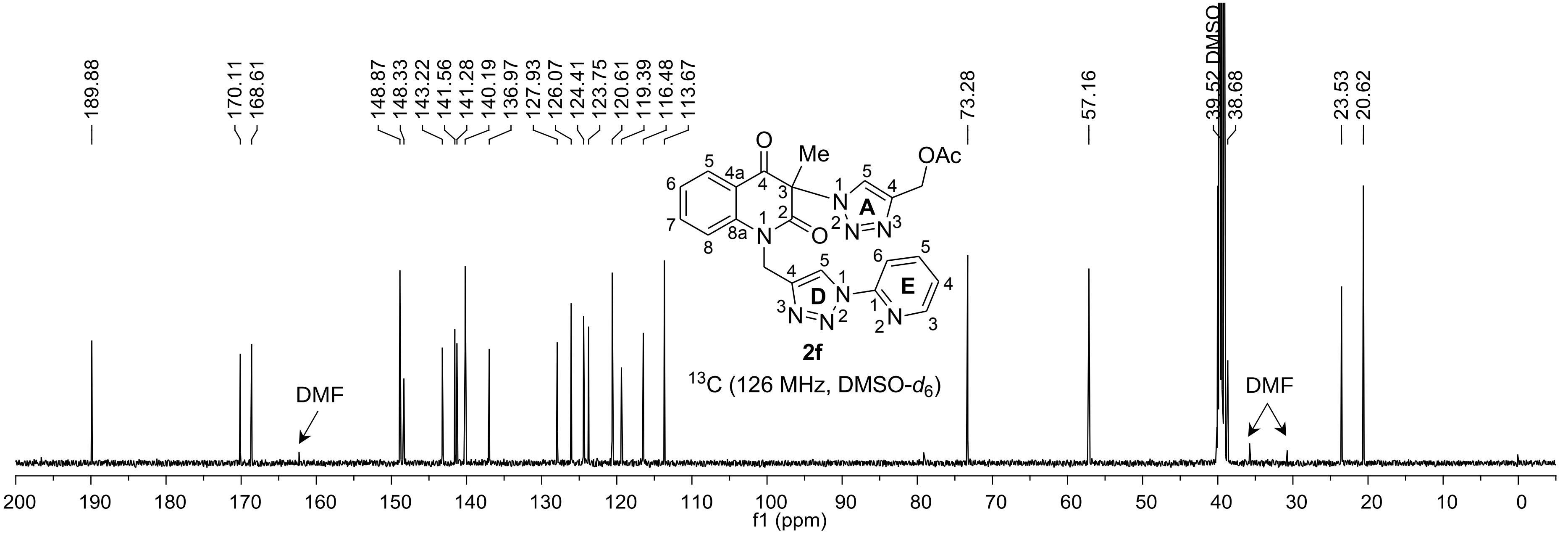
189.88
170.11 168.61
148.87 148.33 143.22 141.56 141.28 140.19 136.97 127.93 126.07 124.41 123.75 120.61 119.39 116.48 113.67
73.28
57.16
39.52 DMSO
38.68
23.53 20.62
DMF
DMF
f1 (ppm)
2f
^{13}C (126 MHz, DMSO-d_6)

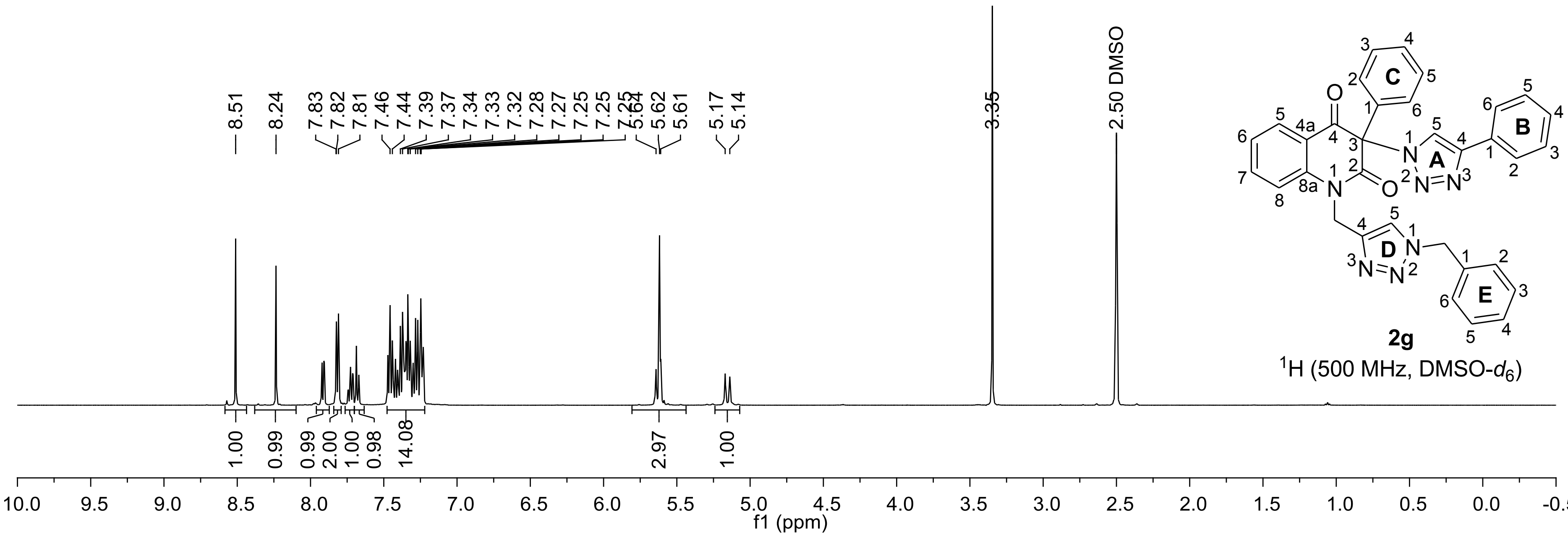
8.51
8.24
7.83
7.82
7.81
7.46
7.44
7.39
7.37
7.34
7.33
7.32
7.28
7.27
7.25
7.25
7.25
5.64
5.62
5.61
5.17
5.14
3.35
2.50 DMSO
1.00
0.99
0.99
2.00
1.00
0.98
14.08
2.97
1.00
f1 (ppm)
2g
^{1}H (500 MHz, DMSO-d_6)

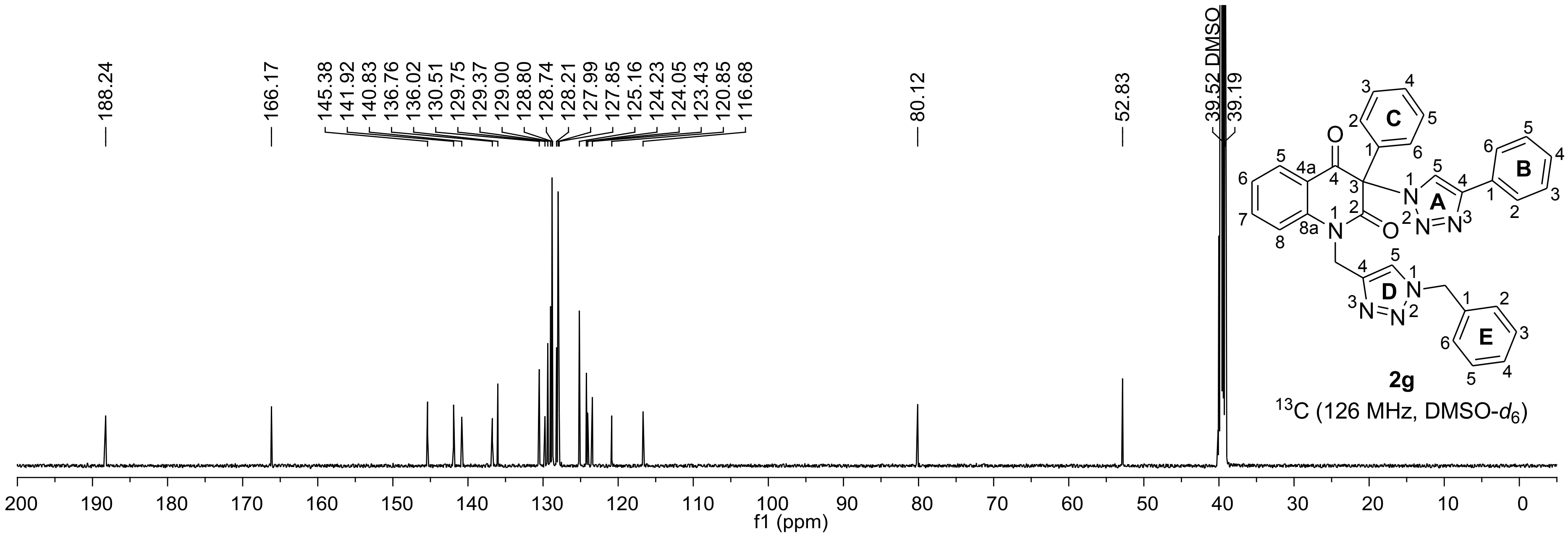
188.24
166.17
145.38
141.92
140.83
136.76
136.02
130.51
129.75
129.37
129.00
128.80
128.74
128.21
127.99
127.85
125.16
124.23
124.05
123.43
120.85
116.68
80.12
52.83
39.52 DMSO
39.19
f1 (ppm)
2g
^{13}C (126 MHz, DMSO-d_6)

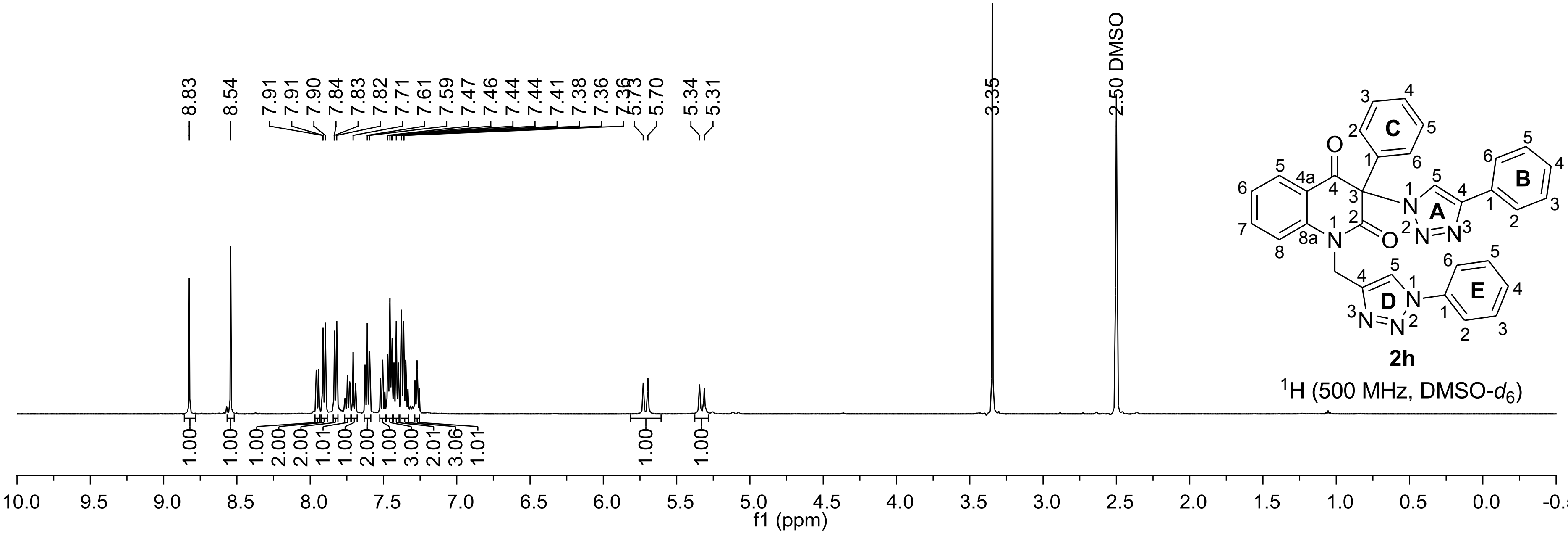
8.83
8.54
7.91
7.91
7.90
7.84
7.83
7.82
7.71
7.61
7.59
7.47
7.46
7.44
7.44
7.41
7.38
7.36
5.73
5.70
5.34
5.31
3.35
2.50 DMSO
1.00
1.00
1.00
2.00
2.00
2.01
1.01
1.00
2.00
1.00
3.00
2.01
3.06
1.01
1.00
1.00
f1 (ppm)
A
B
C
D
E
2h
1H (500 MHz, DMSO-d6)

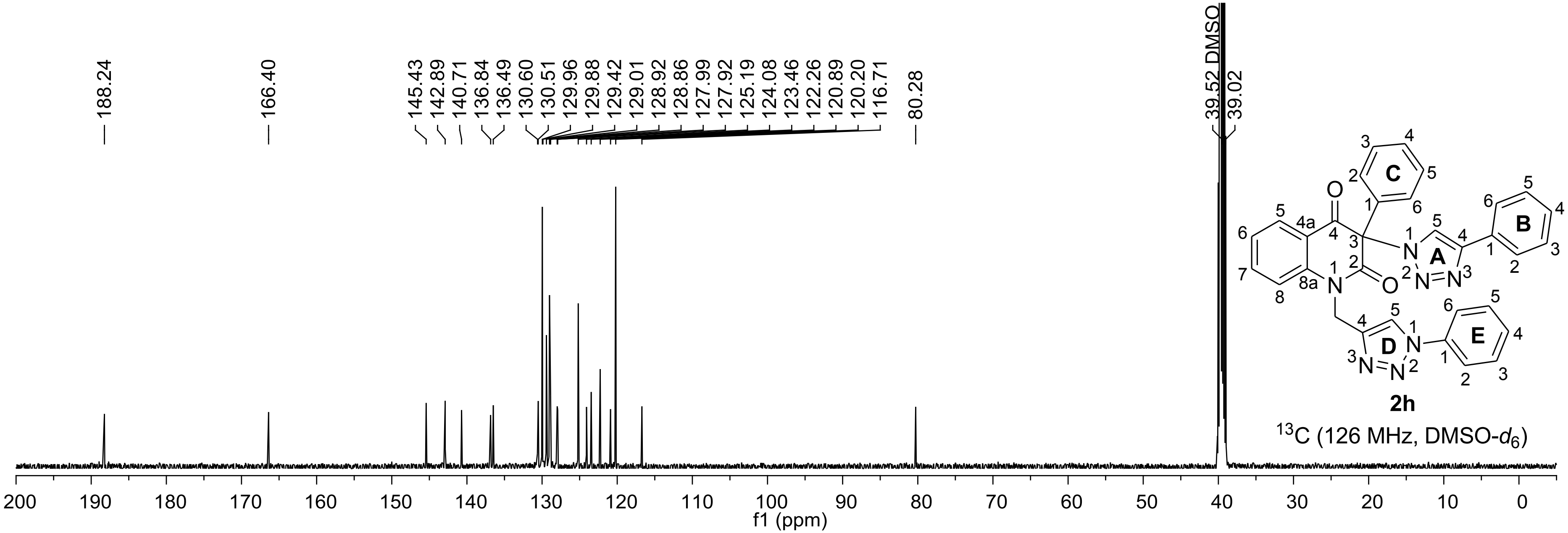
188.24
166.40
145.43
142.89
140.71
136.84
136.49
130.60
130.51
129.96
129.88
129.42
129.01
128.92
128.86
127.99
127.92
125.19
124.08
123.46
122.26
120.89
120.20
116.71
80.28
39.52 DMSO
39.02
f1 (ppm)
A
B
C
D
E
2h
13C (126 MHz, DMSO-d6)

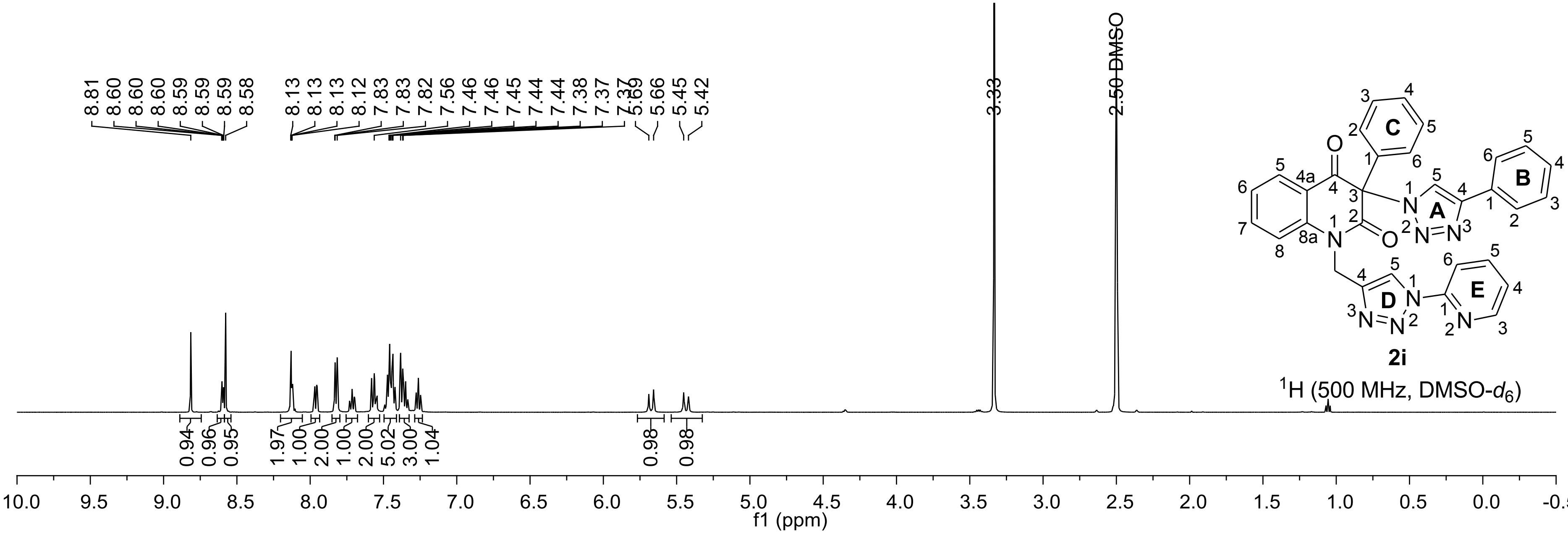
8.81
8.60
8.60
8.60
8.59
8.59
8.59
8.58
8.13
8.13
8.13
8.12
7.83
7.83
7.82
7.56
7.46
7.46
7.45
7.44
7.44
7.38
7.37
7.37
5.69
5.66
5.45
5.42
3.33
2.50 DMSO
0.94
0.96
0.95
1.97
1.00
2.00
1.00
2.00
5.02
3.00
1.04
0.98
0.98
f1 (ppm)
A
B
C
D
E
2i
^{1}H (500 MHz, DMSO-d_6)

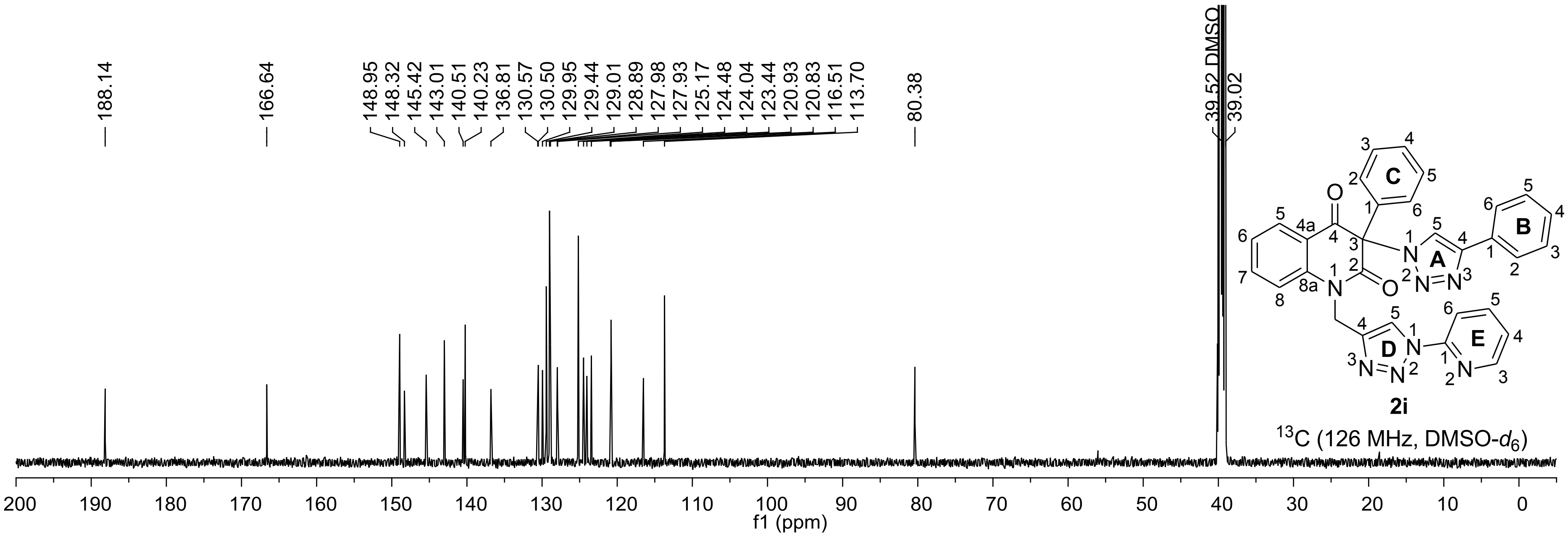
188.14
166.64
148.95
148.32
145.42
143.01
140.51
140.23
136.81
130.57
130.50
129.95
129.44
129.01
128.89
127.98
127.93
125.17
124.48
124.04
123.44
120.93
120.83
116.51
113.70
80.38
39.52 DMSO
39.02
f1 (ppm)
A
B
C
D
E
2i
^{13}C (126 MHz, DMSO-d_6)

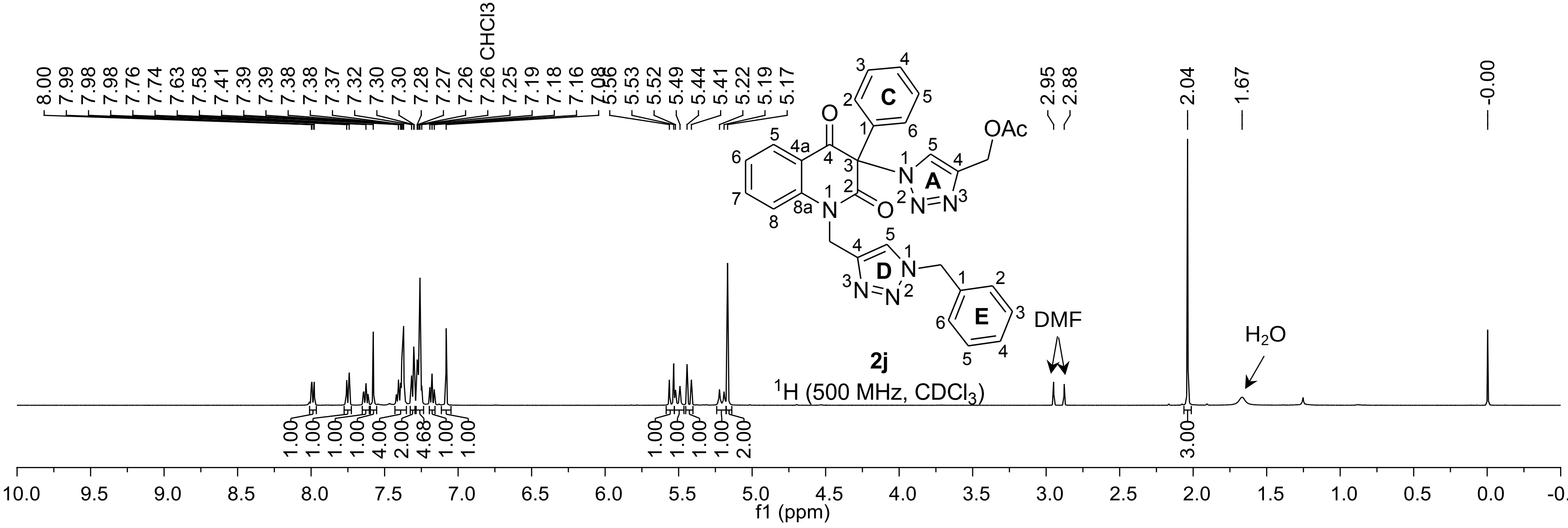
8.00
7.99
7.98
7.98
7.76
7.74
7.63
7.58
7.41
7.39
7.39
7.38
7.38
7.37
7.32
7.30
7.30
7.28
7.27
7.26
7.26 CHCl3
7.25
7.19
7.18
7.16
7.08
5.56
5.53
5.52
5.49
5.44
5.41
5.22
5.19
5.17
2.95
2.88
2.04
1.67
-0.00
OAc
DMF
H_2O
2j
^{1}H (500 MHz, $CDCl_3$)
1.00
1.00
1.00
1.00
4.00
2.00
4.68
1.00
1.00
1.00
1.00
1.00
1.00
2.00
3.00
f1 (ppm)

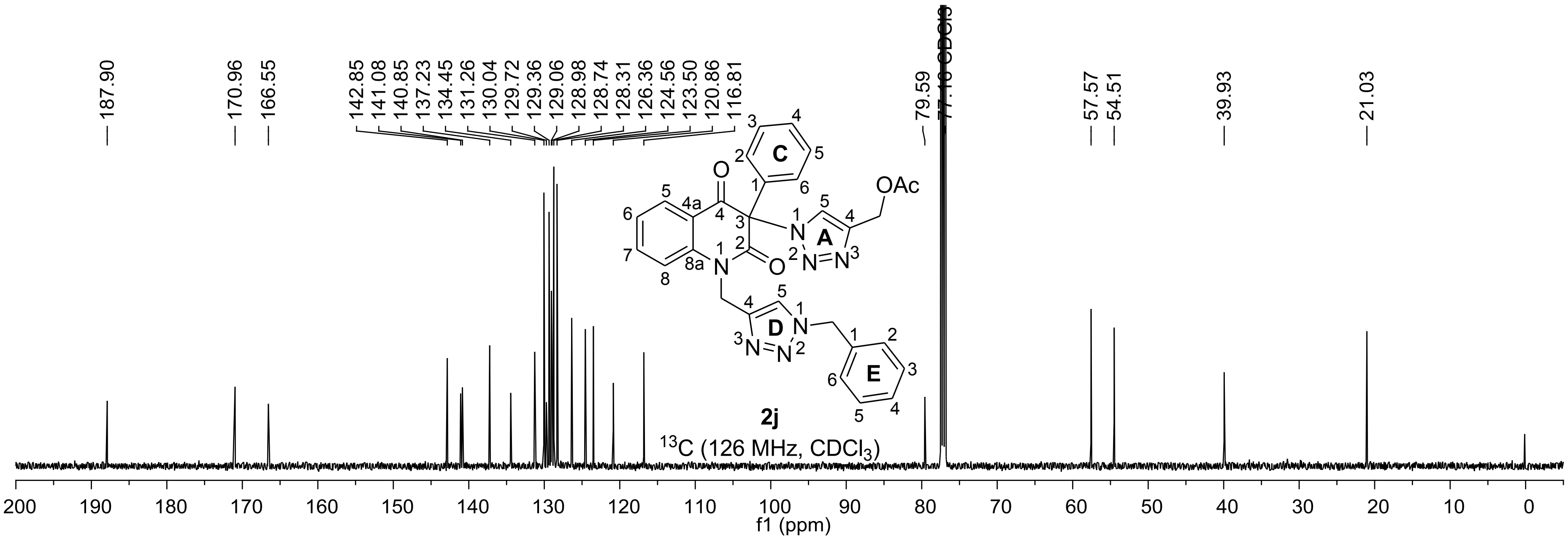
187.90
170.96
166.55
142.85
141.08
140.85
137.23
134.45
131.26
130.04
129.72
129.36
129.06
128.98
128.74
128.31
126.36
124.56
123.50
120.86
116.81
79.59
57.57
54.51
39.93
21.03
OAc
2j
^{13}C (126 MHz, $CDCl_3$)
f1 (ppm)

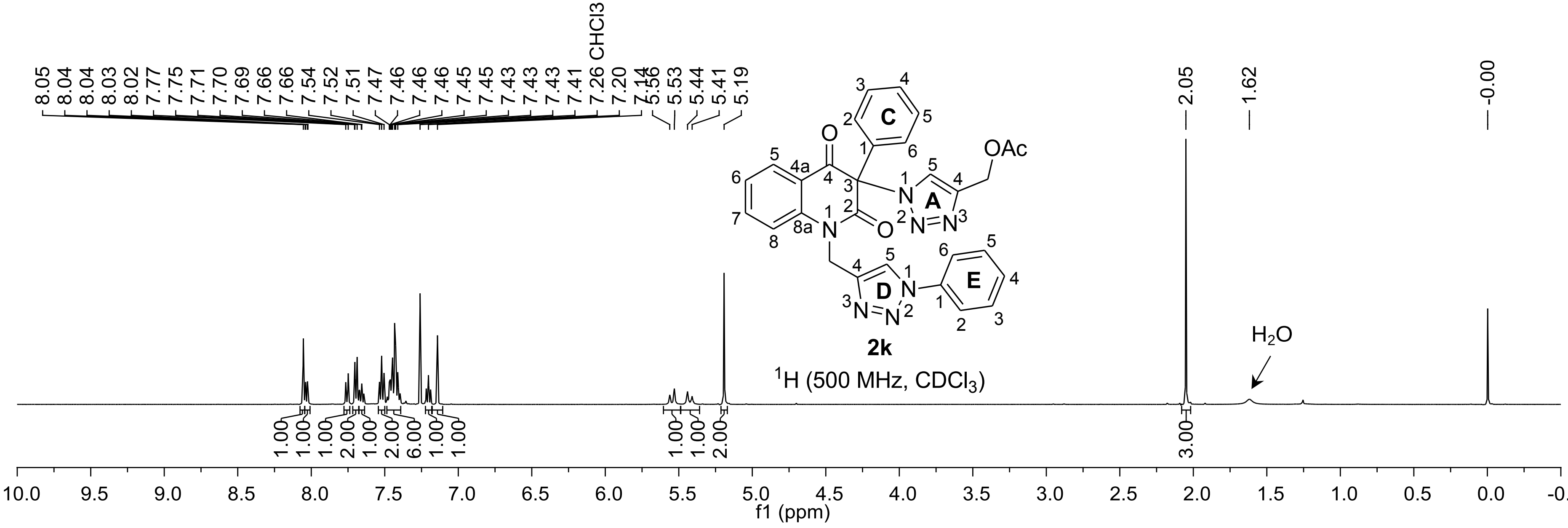
8.05 8.04 8.04 8.03 8.02 7.77 7.75 7.71 7.70 7.69 7.66 7.66 7.54 7.52 7.51 7.47 7.46 7.46 7.46 7.45 7.45 7.43 7.43 7.43 7.41 7.26 CHCl3 7.20 7.14 5.56 5.53 5.44 5.41 5.19
2.05
1.62
-0.00
OAc
2k
^{1}H (500 MHz, CDCl$_3$)
H$_2$O
1.00 1.00 1.00 2.00 1.00 2.00 6.00 1.00 1.00
1.00 1.00 2.00
3.00
f1 (ppm)

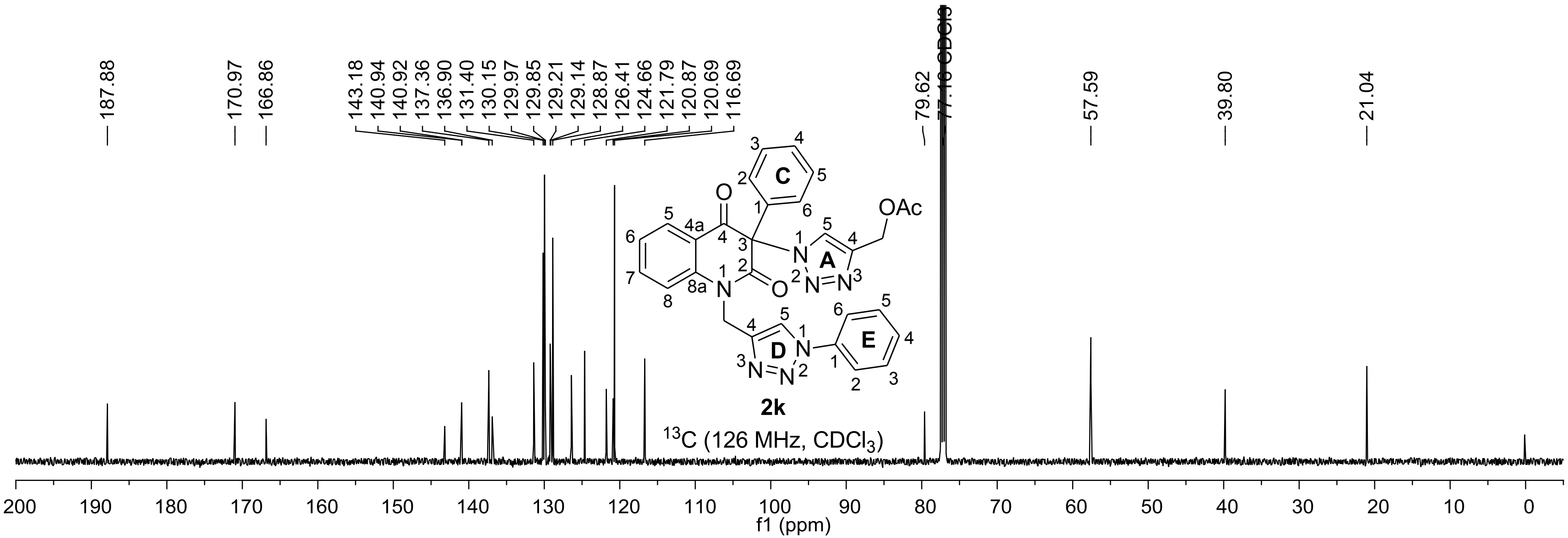
187.88
170.97
166.86
143.18 140.94 140.92 137.36 136.90 131.40 130.15 129.97 129.85 129.21 129.14 128.87 126.41 124.66 121.79 120.87 120.69 116.69
79.62
77.16 CDCl3
57.59
39.80
21.04
OAc
2k
^{13}C (126 MHz, CDCl$_3$)
f1 (ppm)

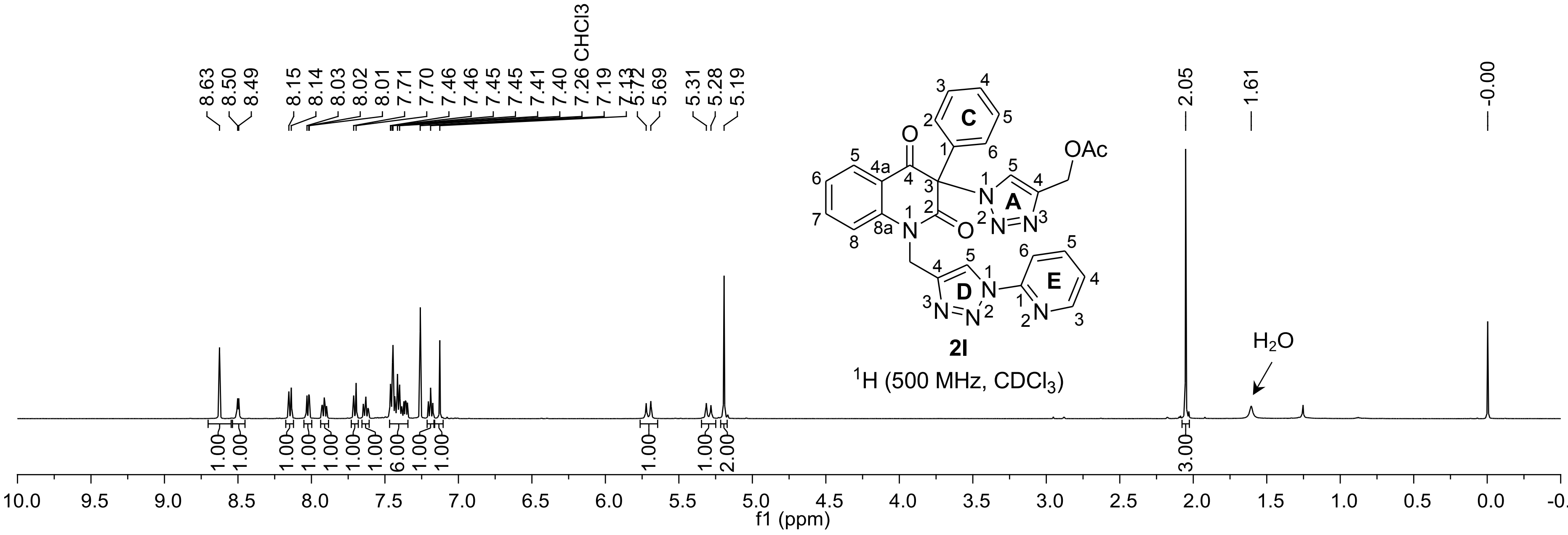
8.63
8.50
8.49
8.15
8.14
8.03
8.02
8.01
7.71
7.70
7.46
7.46
7.45
7.45
7.41
7.40
7.26 CHCl3
7.19
7.13
5.72
5.69
5.31
5.28
5.19
2.05
1.61
-0.00
OAc
2l
H2O
1H (500 MHz, CDCl3)
f1 (ppm)

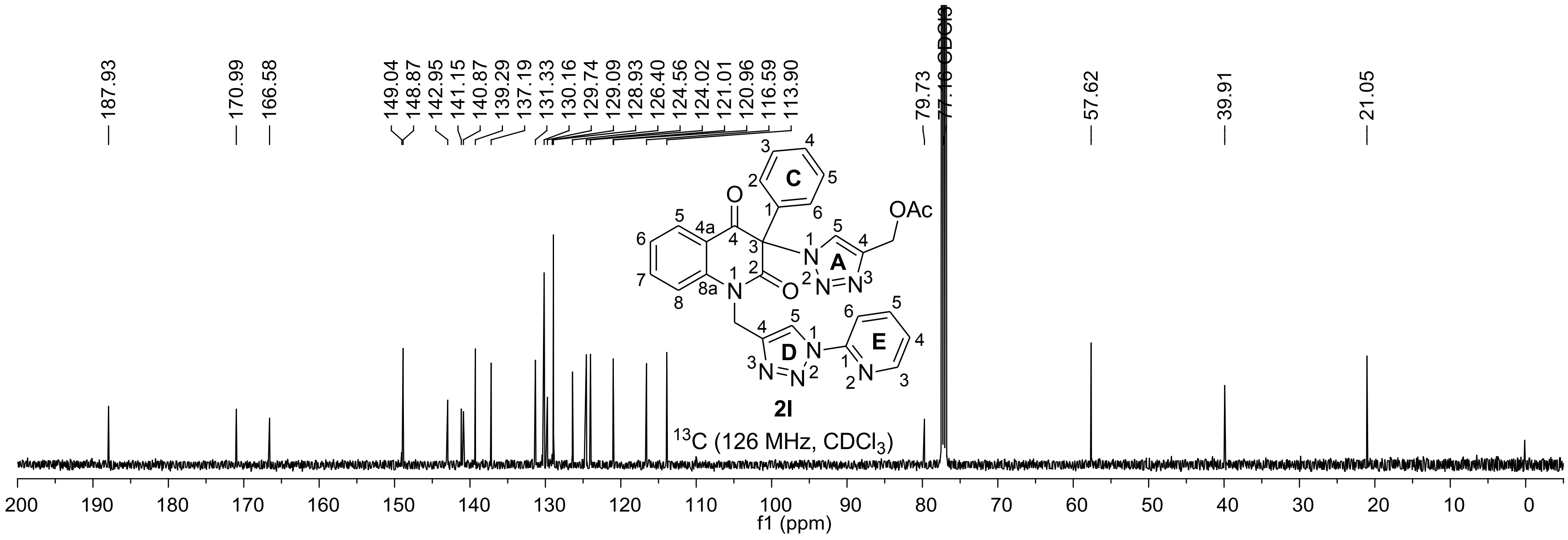
187.93
170.99
166.58
149.04
148.87
142.95
141.15
140.87
139.29
137.19
131.33
130.16
129.74
129.09
128.93
126.40
124.56
124.02
121.01
120.96
116.59
113.90
79.73
77.16 CDCl3
57.62
39.91
21.05
OAc
2l
13C (126 MHz, CDCl3)
f1 (ppm)

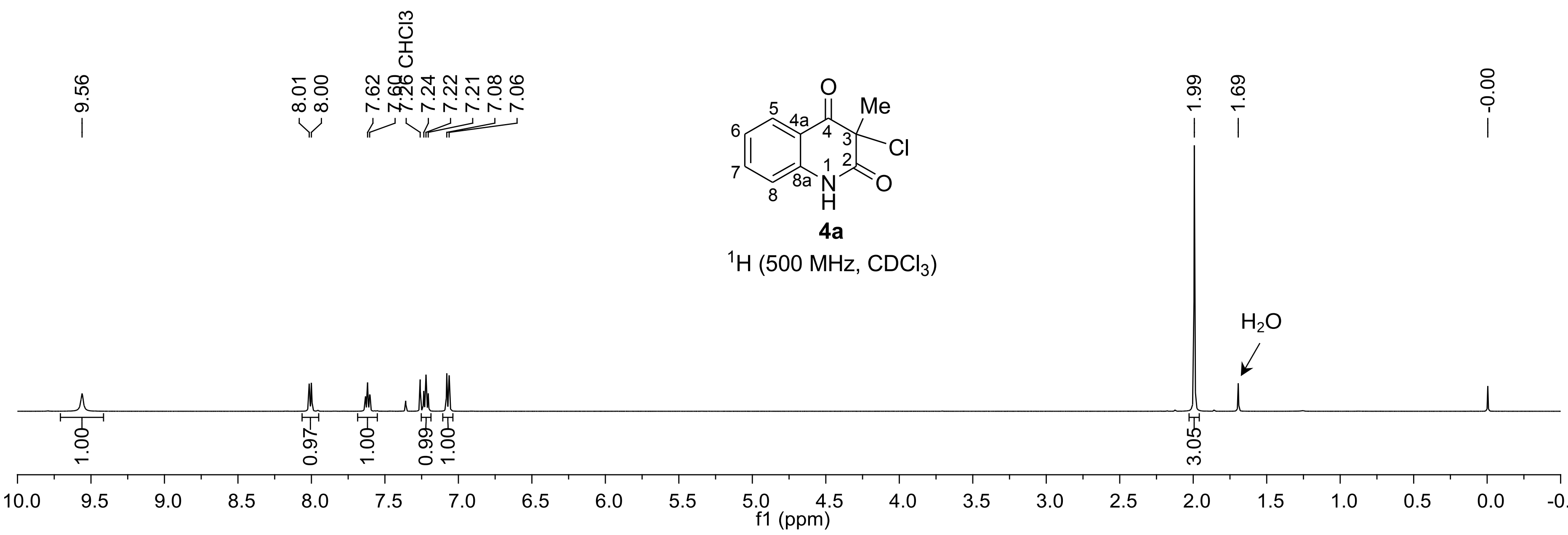
9.56
8.01
8.00
7.62
7.60
7.26 CHCl3
7.24
7.22
7.21
7.08
7.06
1.99
1.69
-0.00
4a
1H (500 MHz, CDCl3)
H2O
1.00
0.97
1.00
0.99
1.00
3.05
f1 (ppm)

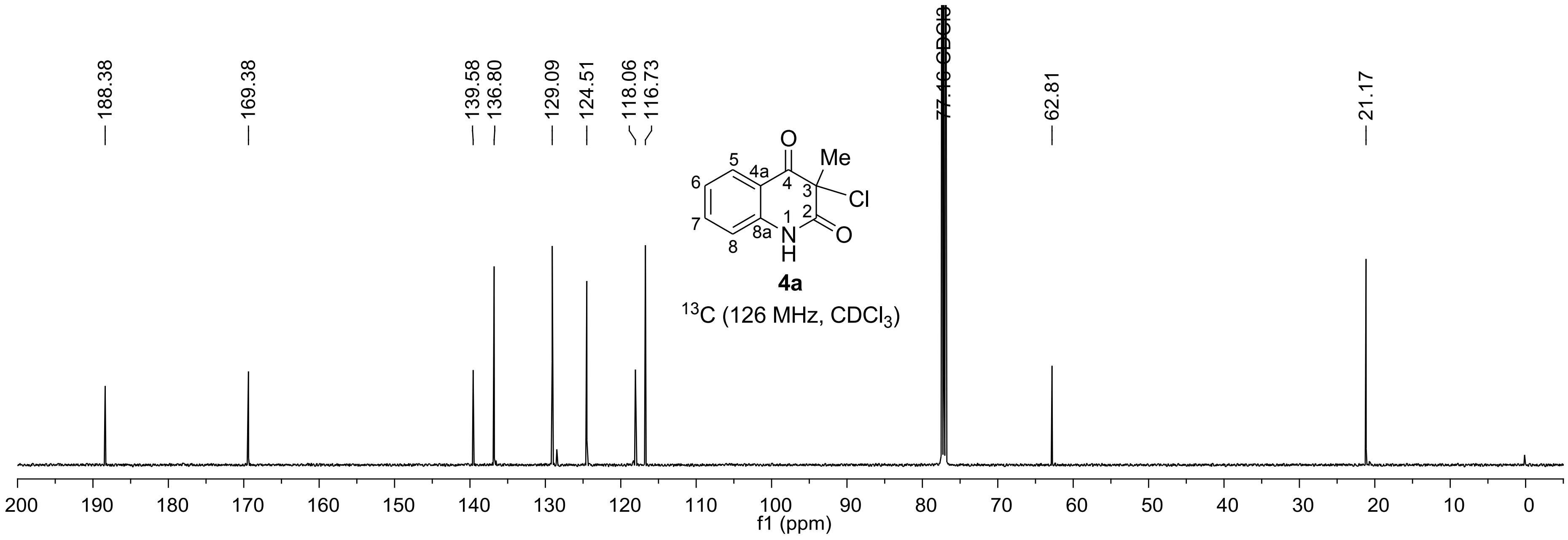
188.38
169.38
139.58
136.80
129.09
124.51
118.06
116.73
77.16 CDCl3
62.81
21.17
4a
13C (126 MHz, CDCl3)
f1 (ppm)

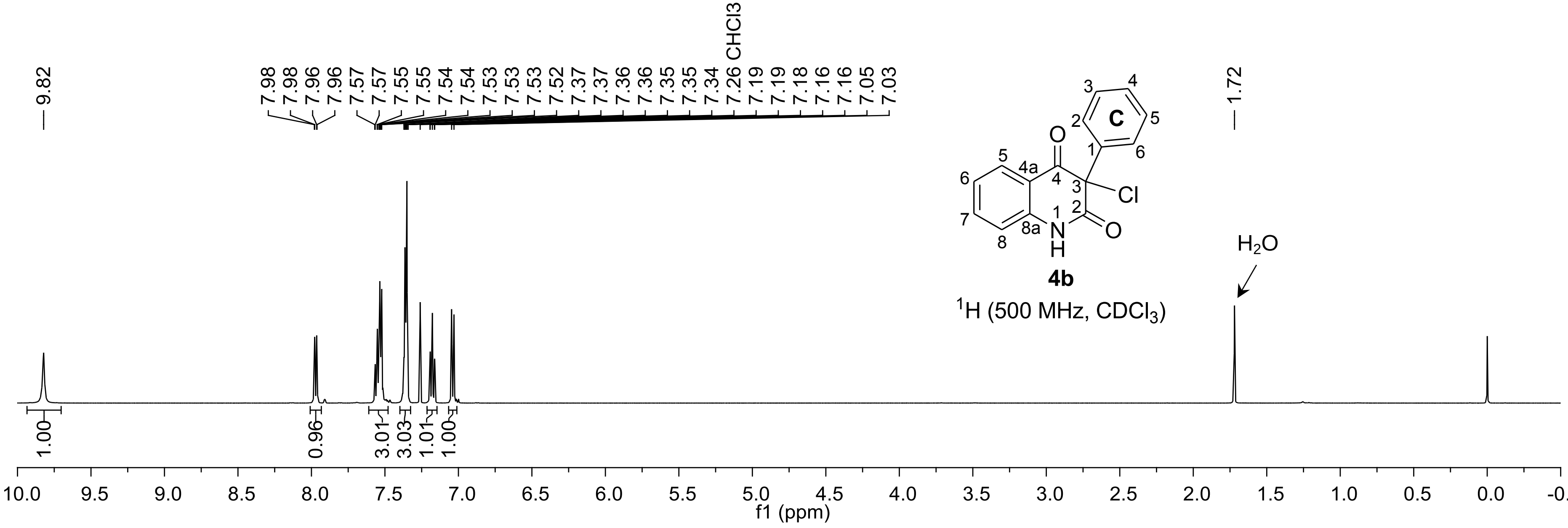
9.82
7.98
7.98
7.96
7.96
7.57
7.57
7.55
7.55
7.54
7.54
7.53
7.53
7.53
7.52
7.37
7.37
7.36
7.36
7.35
7.35
7.34
7.26 CHCl3
7.19
7.19
7.18
7.16
7.16
7.05
7.03
1.72
4b
^{1}H (500 MHz, CDCl$_3$)
H$_2$O
1.00
0.96
3.01
3.03
1.01
1.00
f1 (ppm)

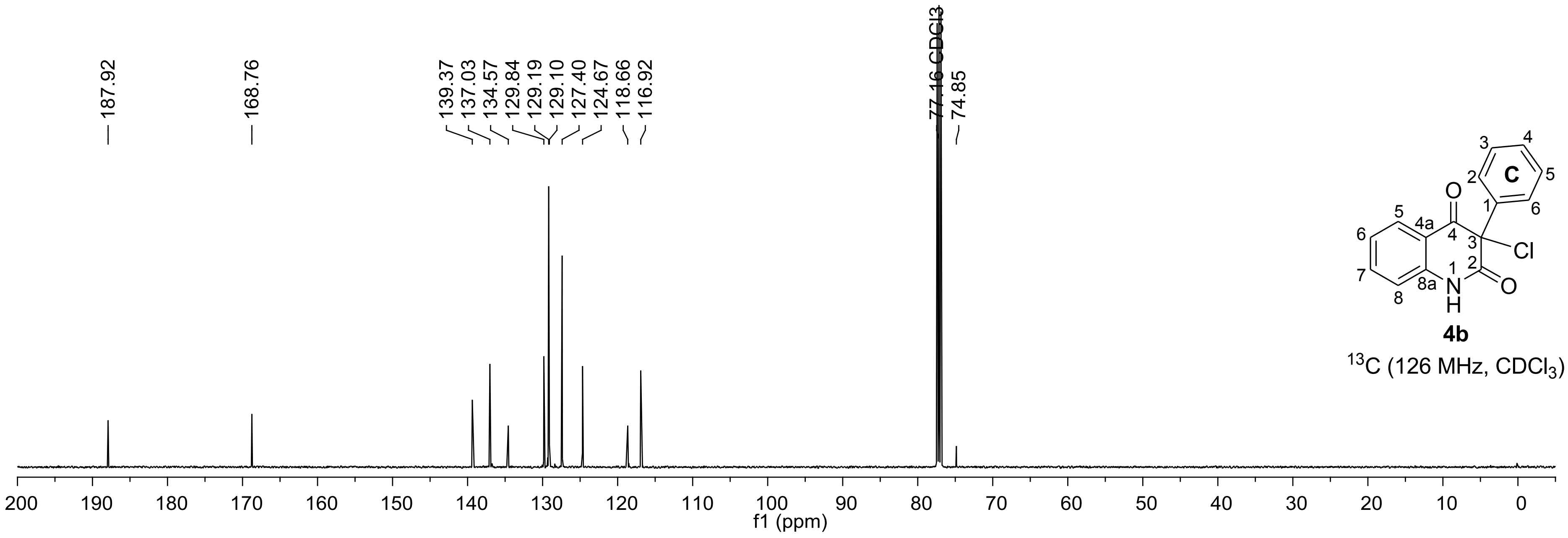
187.92
168.76
139.37
137.03
134.57
129.84
129.19
129.10
127.40
124.67
118.66
116.92
77.16 CDCl3
74.85
4b
^{13}C (126 MHz, CDCl$_3$)
f1 (ppm)

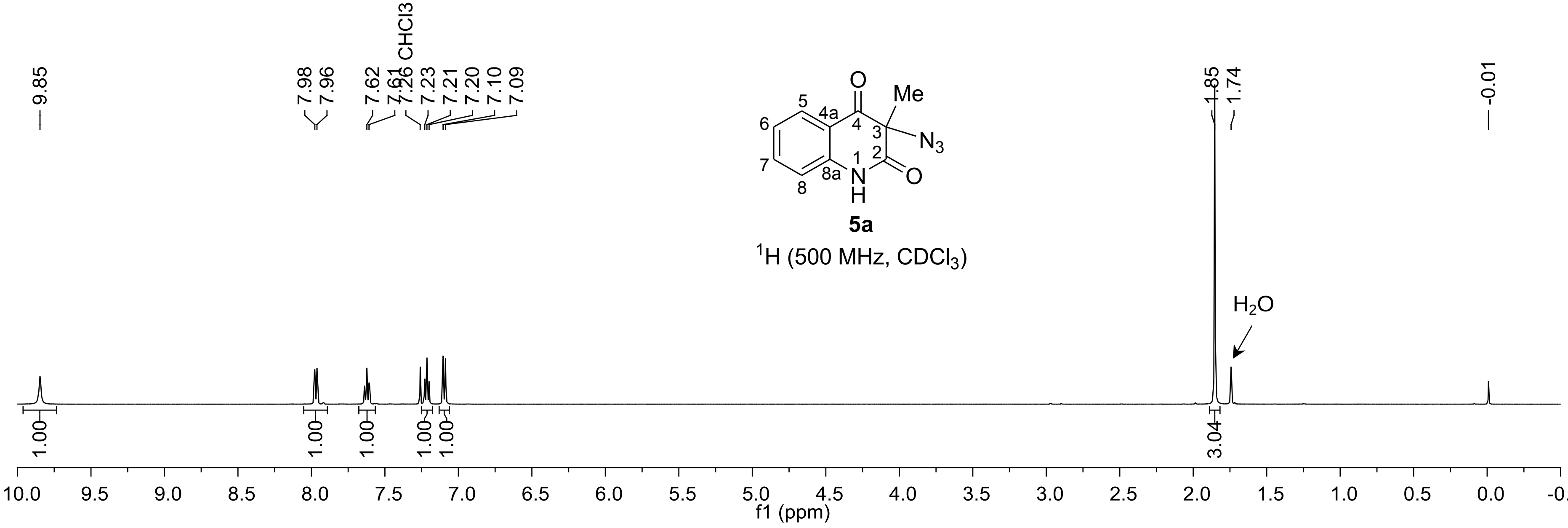

9.85
7.98
7.96
7.62
7.61
7.26 CHCl3
7.23
7.21
7.20
7.10
7.09
1.85
1.74
-0.01
5a
1H (500 MHz, CDCl3)
H2O
1.00
1.00
1.00
1.00
1.00
3.04
f1 (ppm)

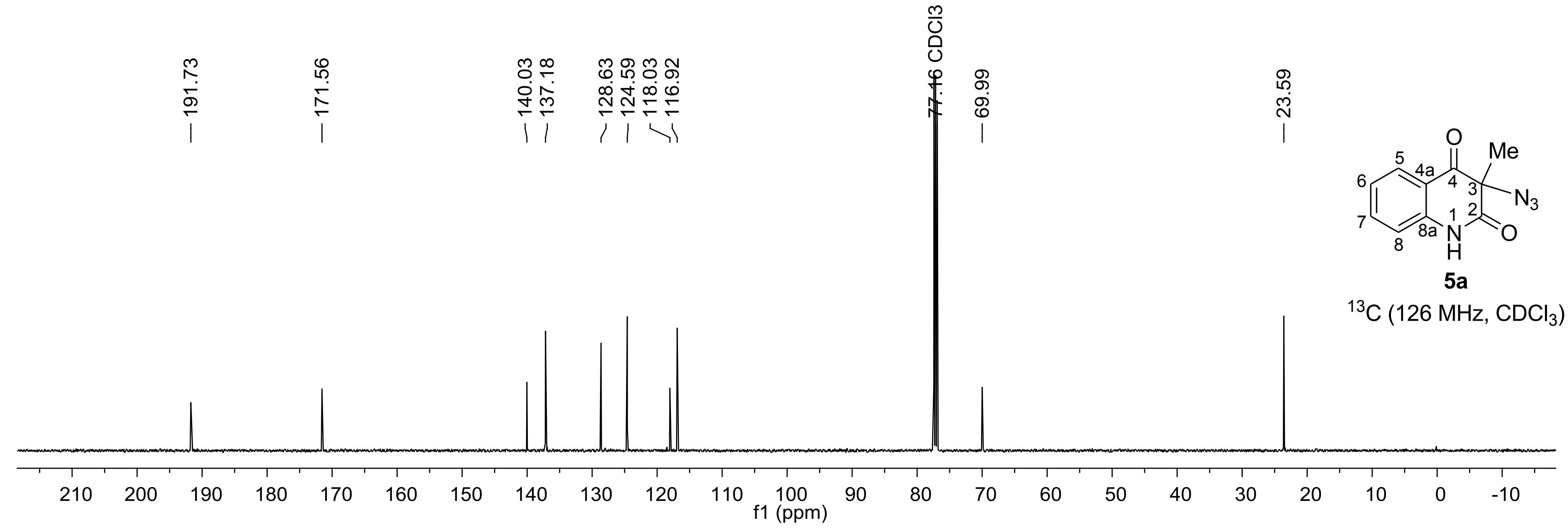

191.73
171.56
140.03
137.18
128.63
124.59
118.03
116.92
77.16 CDCl3
69.99
23.59
5a
13C (126 MHz, CDCl3)
f1 (ppm)

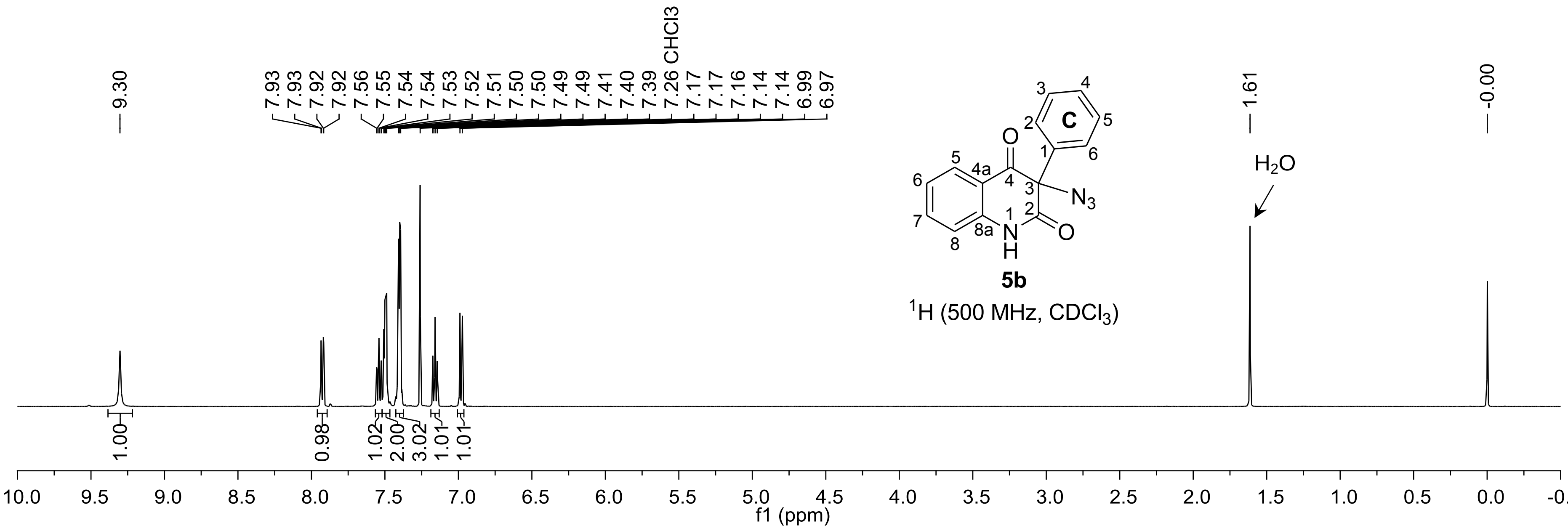
9.30
7.93
7.93
7.92
7.92
7.56
7.55
7.54
7.54
7.53
7.52
7.51
7.50
7.50
7.49
7.49
7.41
7.40
7.39
7.26 CHCl3
7.17
7.17
7.16
7.14
7.14
6.99
6.97
1.61
-0.00
H2O
5b
1H (500 MHz, CDCl3)
1.00
0.98
1.02
2.00
3.02
1.01
1.01
f1 (ppm)

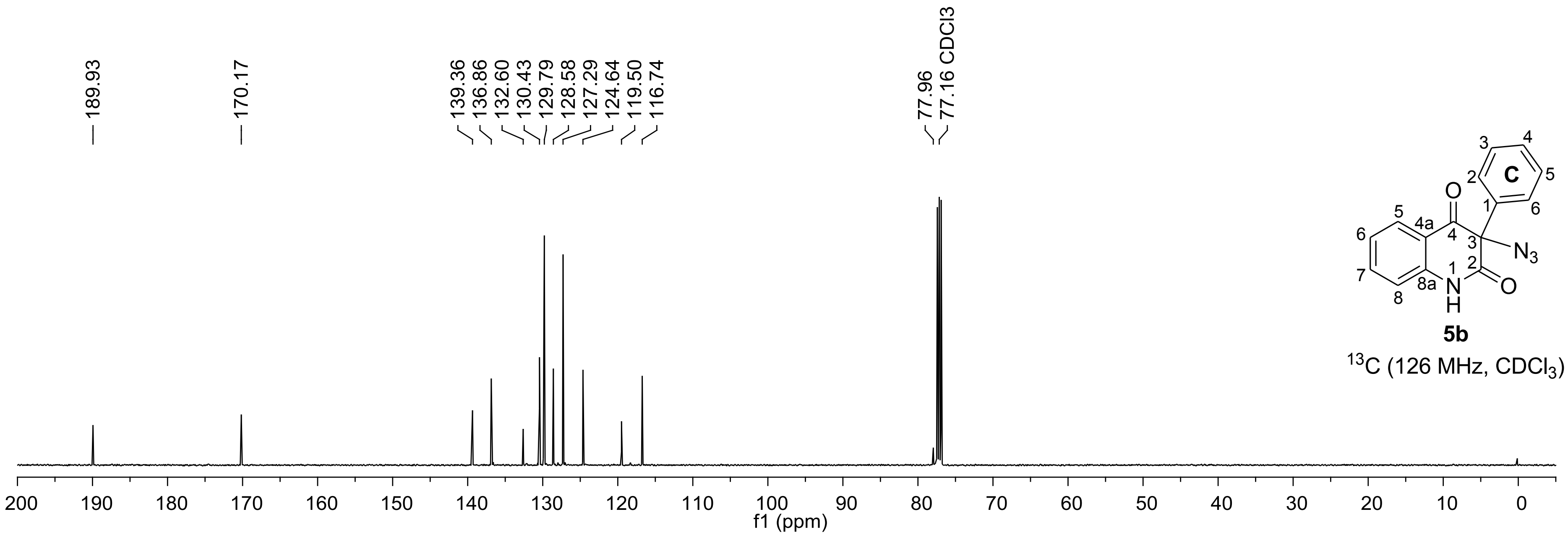
189.93
170.17
139.36
136.86
132.60
130.43
129.79
128.58
127.29
124.64
119.50
116.74
77.96
77.16 CDCl3
5b
13C (126 MHz, CDCl3)
f1 (ppm)

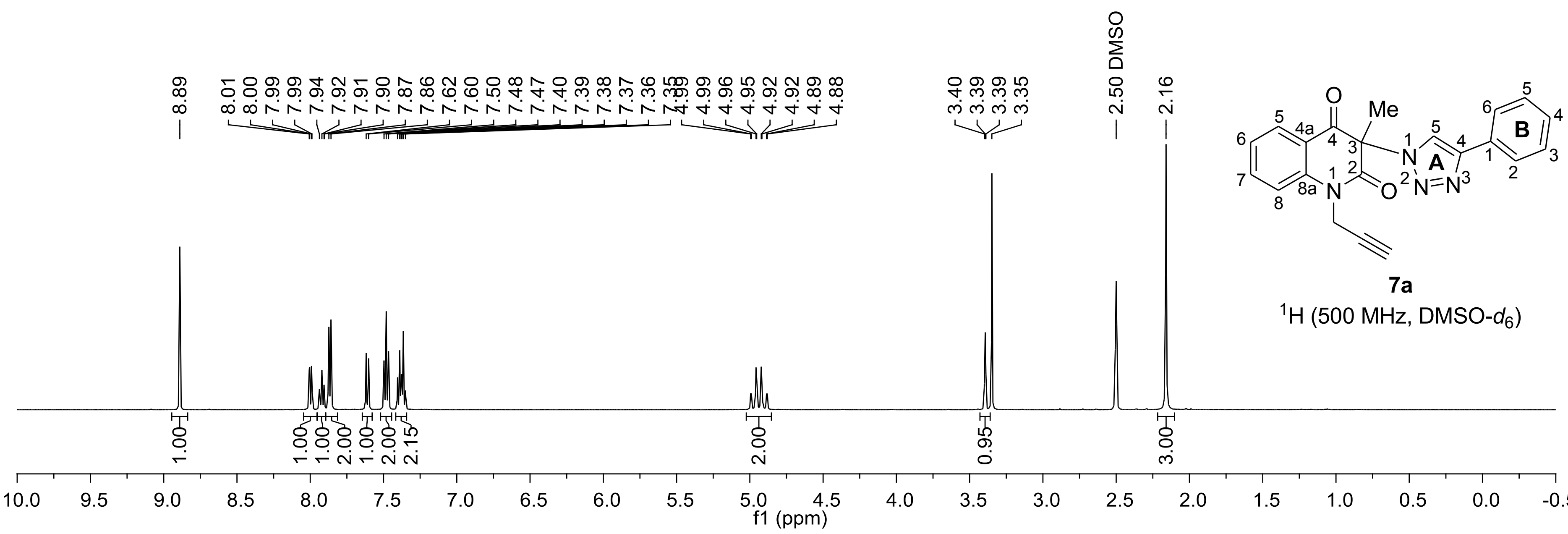
8.89
8.01 8.00 7.99 7.99 7.94 7.92 7.91 7.90 7.87 7.86 7.62 7.60 7.50 7.48 7.47 7.40 7.39 7.38 7.37 7.36 7.35 4.99 4.99 4.96 4.95 4.92 4.92 4.89 4.88
3.40 3.39 3.39 3.35
2.50 DMSO
2.16
1.00 1.00 1.00 2.00 1.00 2.00 2.15 2.00 0.95 3.00
f1 (ppm)
Me
B
A
7a
^{1}H (500 MHz, DMSO-d_6)

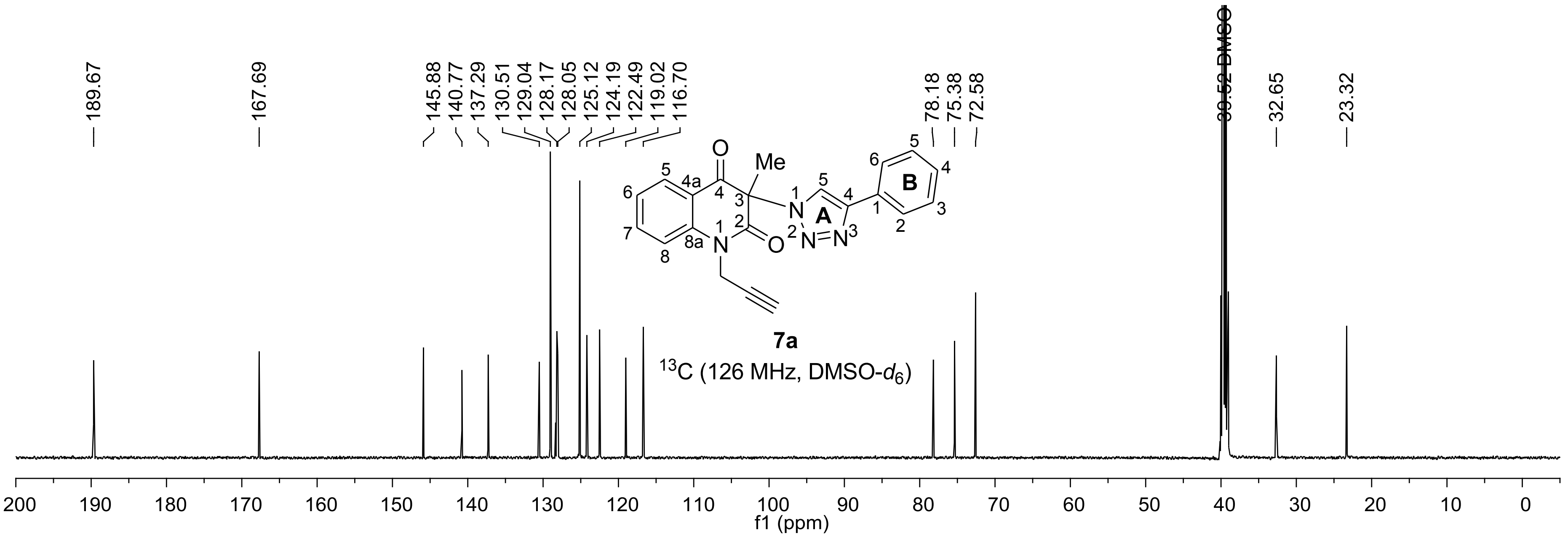
189.67
167.69
145.88 140.77 137.29 130.51 129.04 128.17 128.05 125.12 124.19 122.49 119.02 116.70
78.18 75.38 72.58
39.52 DMSO
32.65
23.32
f1 (ppm)
Me
B
A
7a
^{13}C (126 MHz, DMSO-d_6)

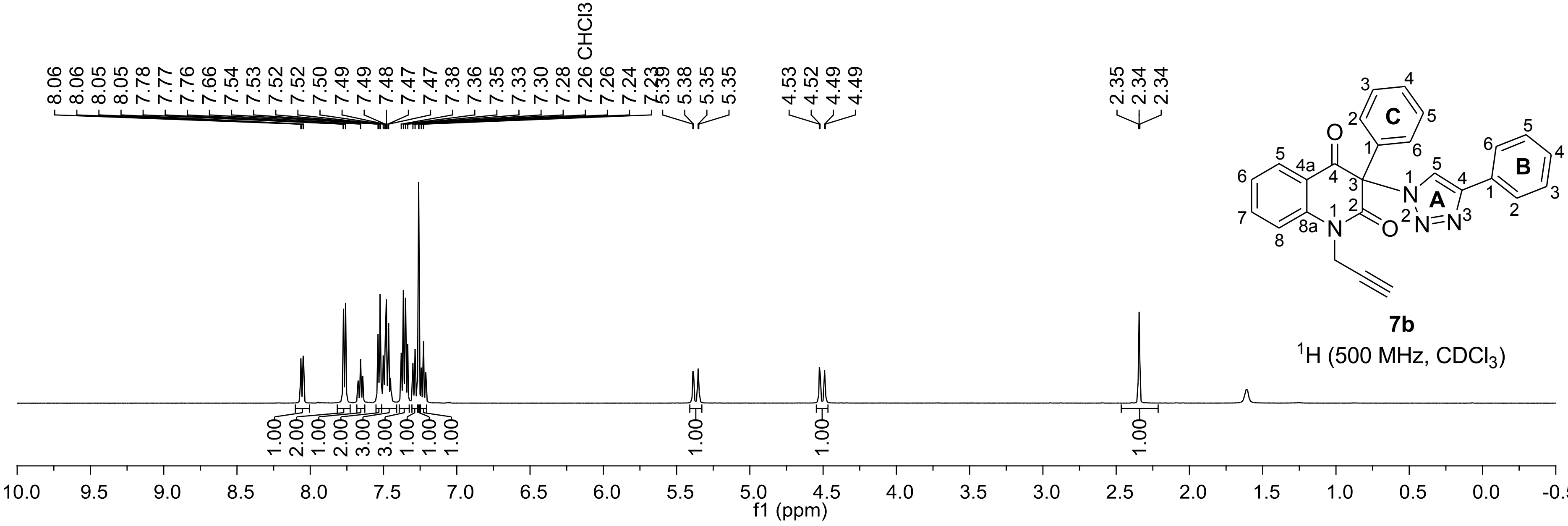
8.06 8.06 8.05 8.05 7.78 7.77 7.76 7.66 7.54 7.53 7.52 7.52 7.50 7.49 7.49 7.48 7.47 7.47 7.38 7.36 7.35 7.33 7.30 7.28 7.26 CHCl3 7.26 7.24 7.23 5.39 5.38 5.35 5.35
4.53 4.52 4.49 4.49
2.35 2.34 2.34
1.00 2.00 1.00 2.00 3.00 3.00 1.00 1.00 1.00
1.00
1.00
1.00
f1 (ppm)
7b
^{1}H (500 MHz, CDCl$_3$)

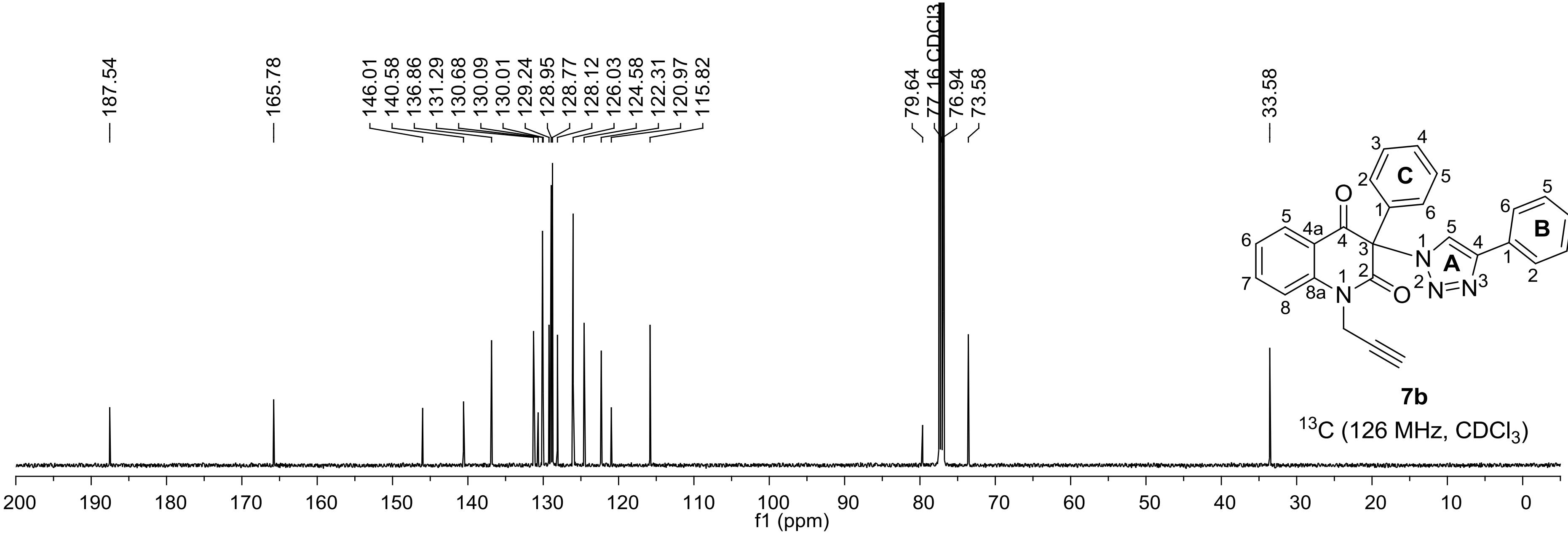
187.54
165.78
146.01 140.58 136.86 131.29 130.68 130.09 130.01 129.24 128.95 128.77 128.12 126.03 124.58 122.31 120.97 115.82
79.64 77.16 CDCl3 76.94 73.58
33.58
f1 (ppm)
7b
^{13}C (126 MHz, CDCl$_3$)

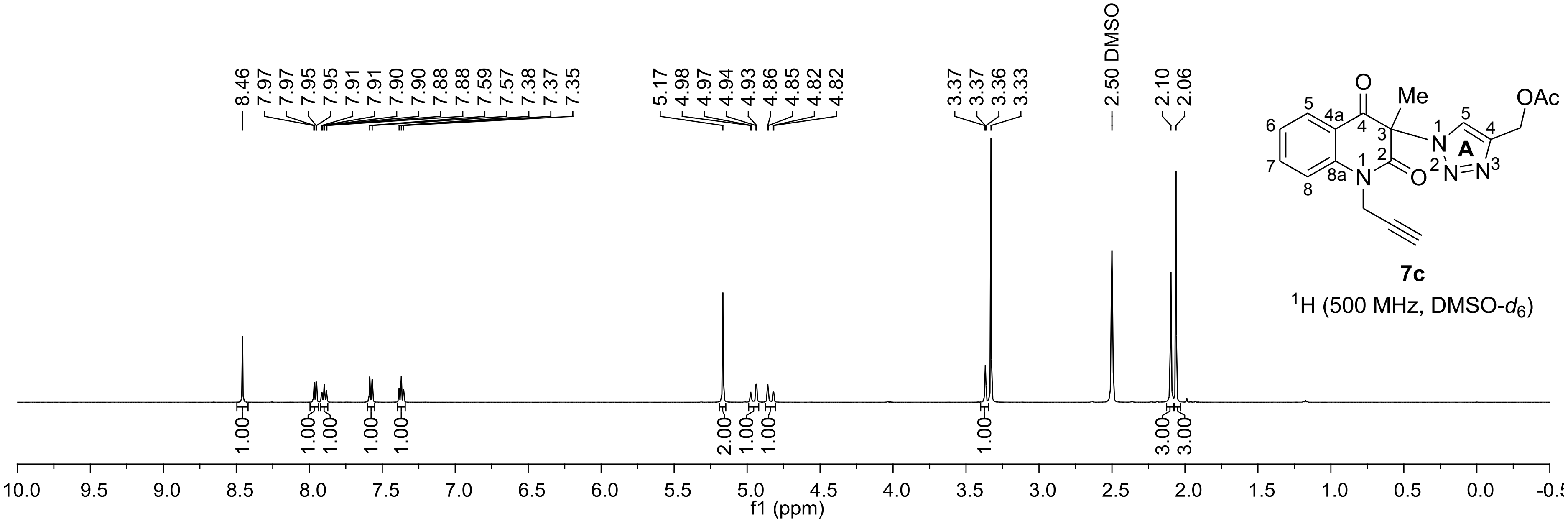

8.46
7.97
7.97
7.95
7.95
7.91
7.91
7.90
7.90
7.88
7.88
7.59
7.57
7.38
7.37
7.35
5.17
4.98
4.97
4.94
4.93
4.86
4.85
4.82
4.82
3.37
3.37
3.36
3.33
2.50 DMSO
2.10
2.06
OAc
Me
7c
^{1}H (500 MHz, DMSO-d_6)
1.00
1.00
1.00
1.00
1.00
2.00
1.00
1.00
1.00
3.00
3.00
f1 (ppm)

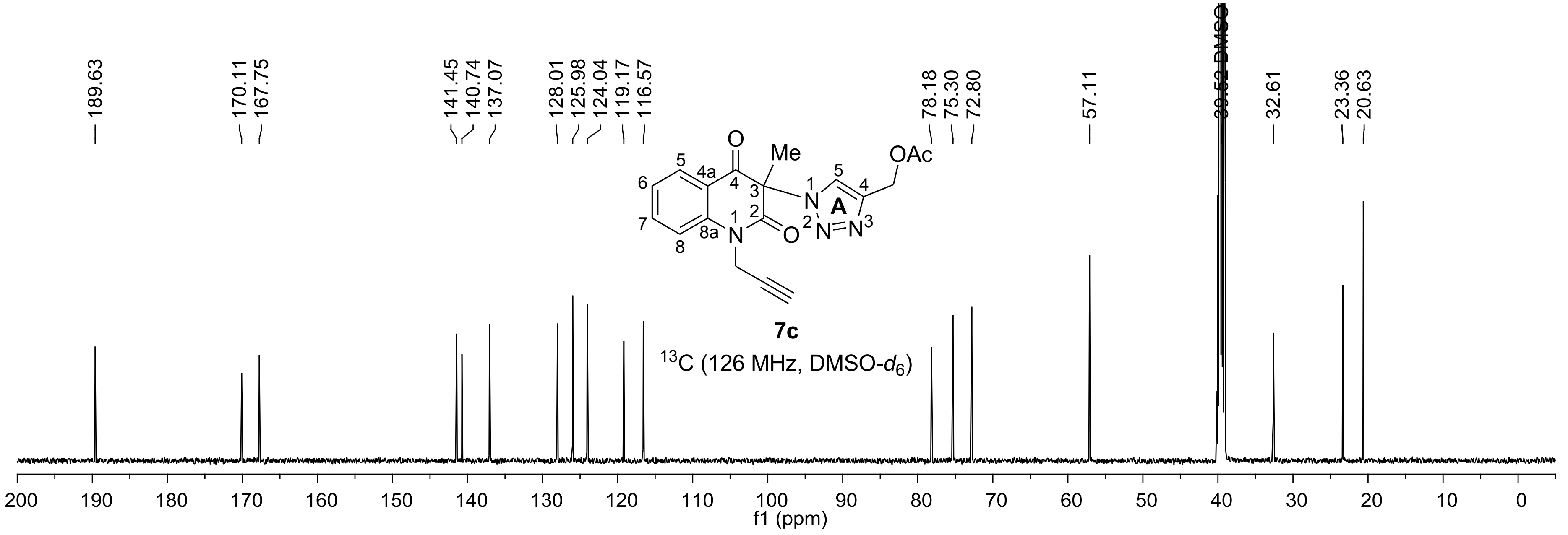

189.63
170.11
167.75
141.45
140.74
137.07
128.01
125.98
124.04
119.17
116.57
78.18
75.30
72.80
57.11
DMSO
32.61
23.36
20.63
OAc
Me
7c
^{13}C (126 MHz, DMSO-d_6)
f1 (ppm)

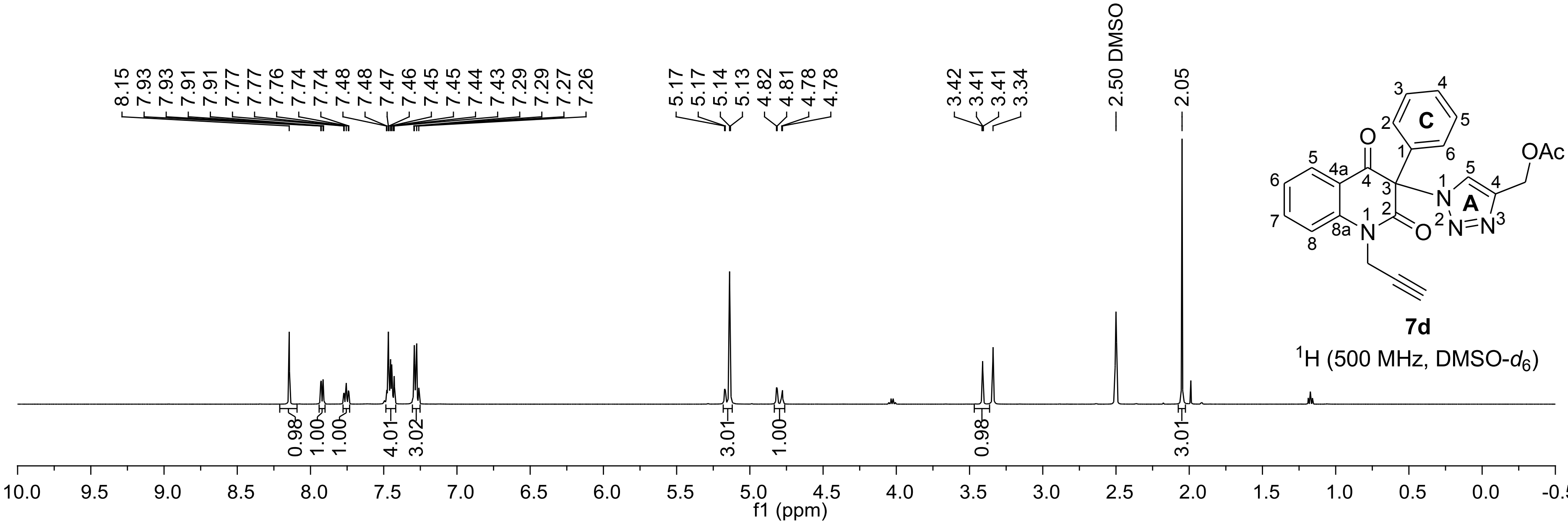
8.15 7.93 7.93 7.91 7.91 7.77 7.77 7.76 7.74 7.74 7.48 7.48 7.47 7.46 7.45 7.45 7.44 7.43 7.29 7.29 7.27 7.26
5.17 5.17 5.14 5.13 4.82 4.81 4.78 4.78
3.42 3.41 3.41 3.34
2.50 DMSO
2.05
0.98 1.00 1.00 4.01 3.02 3.01 1.00 0.98 3.01
10.0 9.5 9.0 8.5 8.0 7.5 7.0 6.5 6.0 5.5 5.0 4.5 4.0 3.5 3.0 2.5 2.0 1.5 1.0 0.5 0.0
f1 (ppm)
OAc
A
C
7d
1H (500 MHz, DMSO-d6)

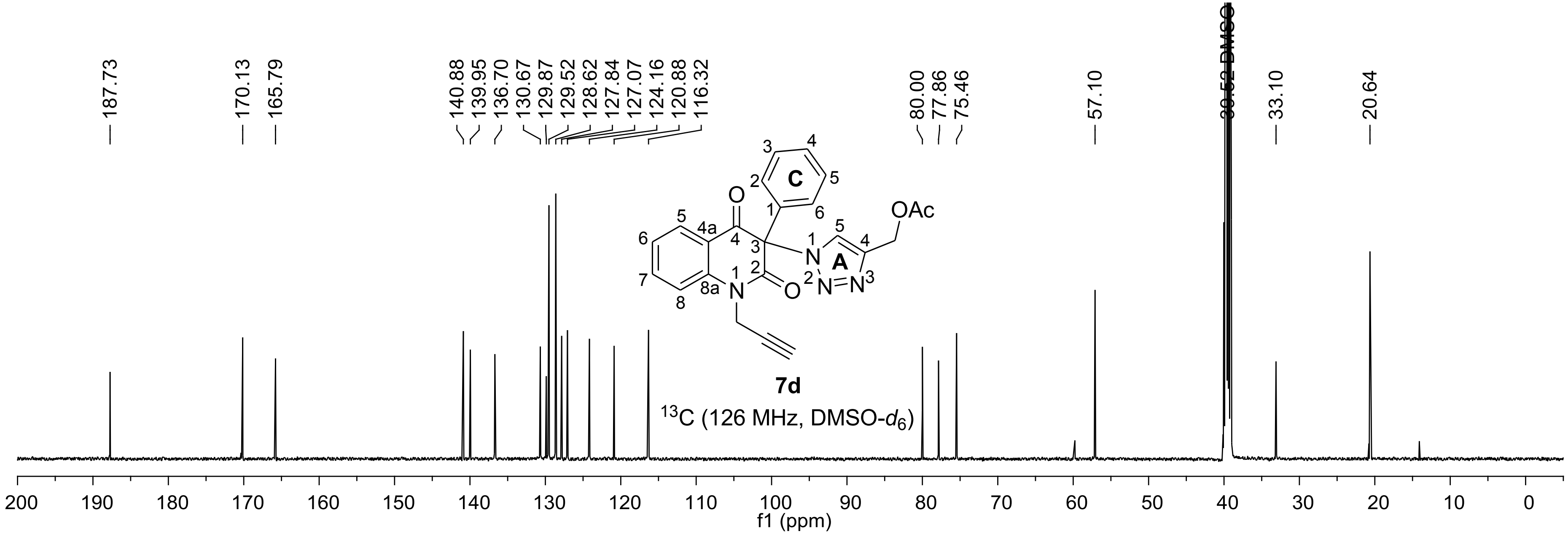
187.73
170.13 165.79
140.88 139.95 136.70 130.67 129.87 129.52 128.62 127.84 127.07 124.16 120.88 116.32
80.00 77.86 75.46
57.10
DMSO
33.10
20.64
200 190 180 170 160 150 140 130 120 110 100 90 80 70 60 50 40 30 20 10 0
f1 (ppm)
OAc
A
C
7d
13C (126 MHz, DMSO-d6)

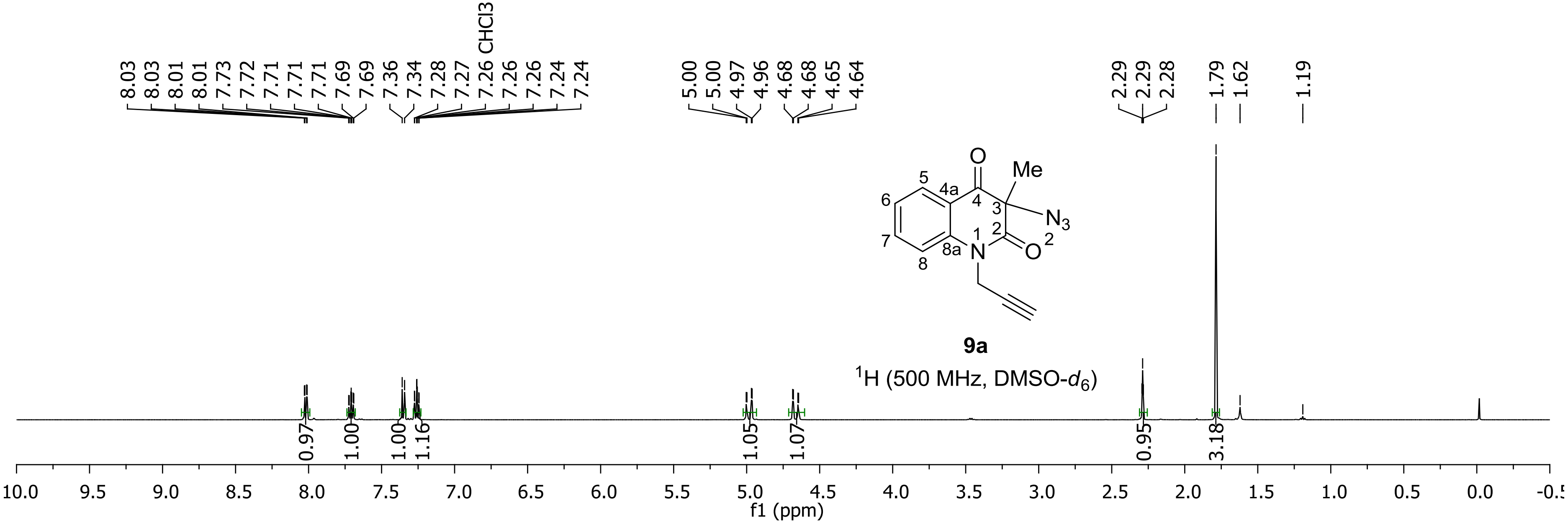
8.03
8.03
8.01
8.01
7.73
7.72
7.71
7.71
7.71
7.69
7.69
7.36
7.34
7.28
7.27
7.26 CHCl3
7.26
7.26
7.24
7.24
5.00
5.00
4.97
4.96
4.68
4.68
4.65
4.64
2.29
2.29
2.28
1.79
1.62
1.19
O
Me
5
4a
6
4
3
N3
2
1
7
8a
N
O
2
8
9a
1H (500 MHz, DMSO-d6)
0.97
1.00
1.00
1.16
1.05
1.07
0.95
3.18
10.0
9.5
9.0
8.5
8.0
7.5
7.0
6.5
6.0
5.5
5.0
4.5
4.0
3.5
3.0
2.5
2.0
1.5
1.0
0.5
0.0
f1 (ppm)

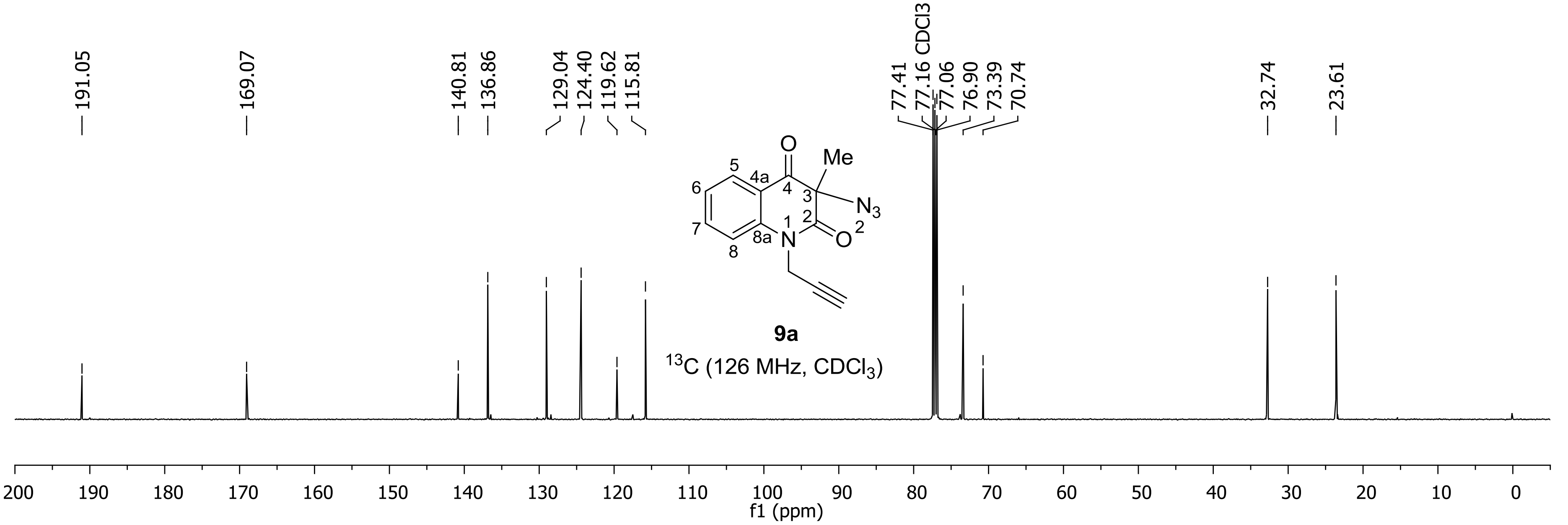
191.05
169.07
140.81
136.86
129.04
124.40
119.62
115.81
77.41
77.16 CDCl3
77.06
76.90
73.39
70.74
32.74
23.61
O
Me
5
4a
6
4
3
N3
2
1
7
8a
N
O
2
8
9a
13C (126 MHz, CDCl3)
200
190
180
170
160
150
140
130
120
110
100
90
80
70
60
50
40
30
20
10
0
f1 (ppm)

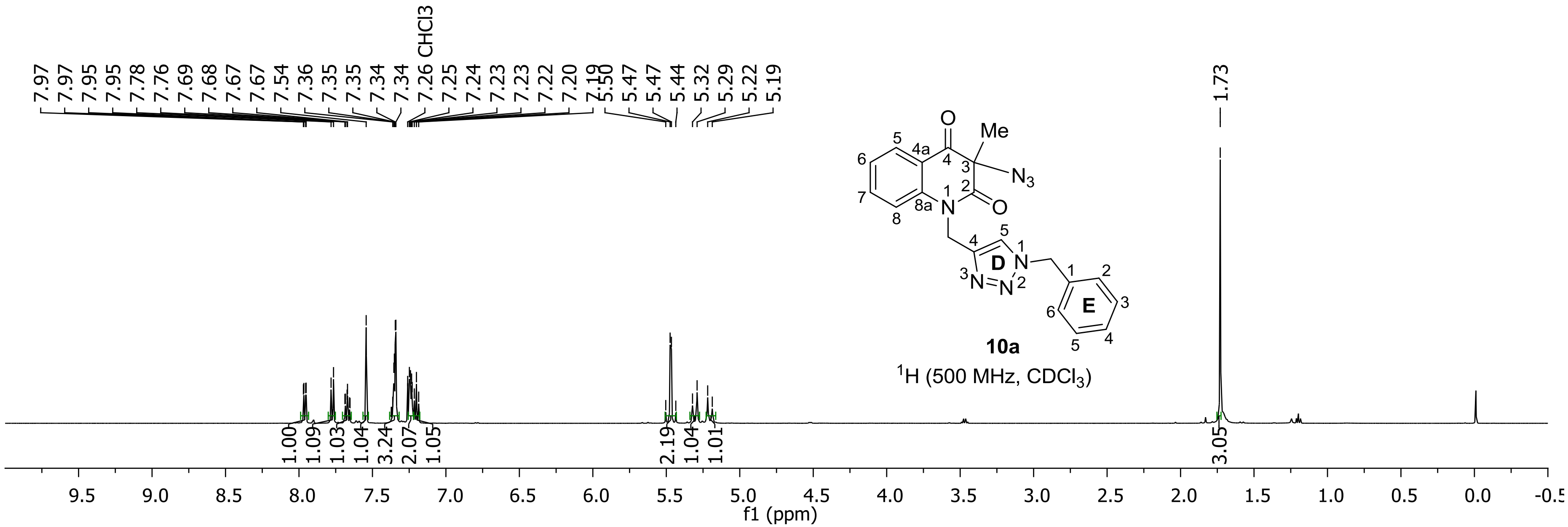

10a

^1H (500 MHz, $CDCl_3$)

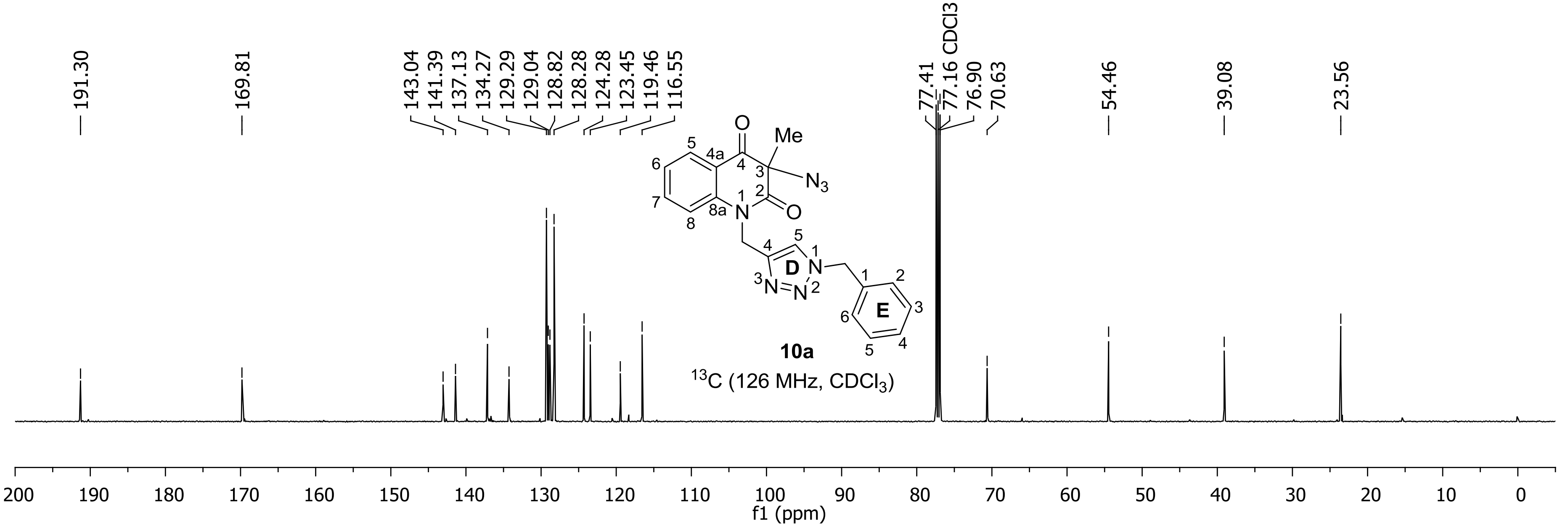

10a

^{13}C (126 MHz, $CDCl_3$)
